# Spectrum of immune checkpoint inhibitors-induced endocrinopathies in cancer patients: a scoping review of case reports

**DOI:** 10.1186/s40842-018-0073-4

**Published:** 2019-01-22

**Authors:** Meng H. Tan, Ravi Iyengar, Kara Mizokami-Stout, Sarah Yentz, Mark P. MacEachern, Li Yan Shen, Bruce Redman, Roma Gianchandani

**Affiliations:** 1 Division of Metabolism, Endocrinology & Diabetes, Department of Internal Medicine, University of Michigan, 24 Frank Lloyd Wright Drive, Ann Arbor, MI 48106 USA; 2 Present address: Endocrinology, Rush University Medical Center, 1725 West Harrison Street, Chicago, IL 60612 USA; 3 Division of Hematology/Oncology, Department of Internal Medicine, University of Michigan, 1500 E. Medical Center Drive, Ann Arbor, MI 48109 USA; 4 Taubman Health Sciences Library, University of Michigan, 1135 Catherine Street, Ann Arbor, MI 48109 USA; 5 Affiliated Hospital of QingDao University, QingDao, 16 Jiangsu Road, Sinan Qu, Qingdao, Shi, Shandong Sheng China

**Keywords:** Cancer immunotherapy, Immune checkpoint inhibitors (ICI), ICI-induced endocrinopathies, Endocrine immune-related AEs

## Abstract

**Background:**

Since 2011 six immune checkpoint inhibitors (ICI) have been approved to treat patients with many advanced solid tumor and hematological malignancies to improve their prognosis. Case reports of their endocrine immune-related adverse events [irAEs]) are increasingly published as more real-world patients with these malignancies are treated with these drugs. They alert physicians of a drug’s AEs (which may change during a drug’s life cycle) and contribute to post-marketing safety surveillance. Using a modified framework of Arksey and O’Malley, we conducted a scoping review of the spectrum and characteristics of ICI-induced endocrinopathies case reports before and after ICIs are marketed.

**Methods:**

In July 2017, we searched, without date and language restrictions, 4 citation databases for ICI-induced endocrinopathies. We also hand-searched articles’ references, contents of relevant journals, and ran supplemental searches to capture recent reports through January 2018. For this study, a case should have information on type of cancer, type of ICI, clinical presentation, biochemical tests, treatment plus temporal association of ICI initiation with endocrinopathies. Two endocrinologists independently extracted the data which were then summarized and categorized.

**Results:**

One hundred seventy nine articles reported 451 cases of ICI-induced endocrinopathies - 222 hypopituitarism, 152 thyroid disorders, 66 diabetes mellitus, 6 primary adrenal insufficiencies, 1 ACTH-dependent Cushing’s syndrome, 1 hypoparathyroidism and 3 diabetes insipidus cases. Their clinical presentations reflect hormone excess or deficiency. Some were asymptomatic and others life-threatening. One or more endocrine glands could be affected. Polyglandular endocrinopathies could present simultaneously or in sequence. Many occur within 5 months of therapy initiation; a few occurred after ICI was stopped. Mostly irreversible, they required long-term hormone replacement. High dose steroids were used when non-endocrine AEs coexisted or as therapy in adrenal insufficiency. There was variability of information in the case reports but all met the study criteria to make a diagnosis.

**Conclusions:**

The spectrum of ICI-induced endocrinopathies is wide (5 glands affected) and their presentation varied (12 endocrinopathies). Clinical reasoning integrating clinical, biochemical and treatment information is needed to properly diagnose and manage them. Physicians should be vigilant for their occurrence and be able to diagnose, investigate and manage them appropriately at onset and follow-up.

**Electronic supplementary material:**

The online version of this article (10.1186/s40842-018-0073-4) contains supplementary material, which is available to authorized users.

## Introduction

Advances in our understanding of the immune response to cancer and mechanisms of immune modulation have been translated to immunotherapy for the treatment of many advanced solid tumor and hematological malignancies. The two most promising advances in cancer immunotherapy are cell-based adoptive therapy modalities and immune regulatory modalities. The immune regulatory modalities currently have a wider therapeutic utility than cell-based therapies. They consist of monoclonal antibodies (mAbs) to proteins known as immune checkpoint regulators - cytotoxic T-lymphocyte-associated antigen-4 (CTLA-4), programmed cell death protein-1 (PD-1) and programmed death ligand 1 and 2 (PD-L1 and PD-L2) [1–6].

PD-1 and CTLA-4 are found on lymphocytes. PD-L1/L2 are found on many cells, including tumor cells. The typical function of these checkpoint regulator proteins is to diminish the immune response to antigen, acting as a brake on the immune system [1–6]. Monoclonal Abs to these checkpoint regulator proteins, known as immune checkpoint inhibitors (ICIs), release the brake that has been placed on the immune system, allowing the patients’ immune system to attack cancer cells and certain healthy tissues. There are currently 6 ICIs approved for the treatment of many advanced cancers (Table 1).

**Table 1:** Immune checkpoint inhibitors approved by the Food and Drug Administration in USA

	Ipilimumab CTLA-4	Nivolumab PD-1	Pembrolizumab PD-1	Atezolizumab PD-L1	Avelumab PD-L1	Durvalumab PD-L1
Malignancy						
Melanoma	√*(3/2011)	√*(12/2014)	√*(9/2014)			
Non-small cell lung cancer		√ (3/2015)	√ (10/2015)	√ (10/2016)		
Renal cell carcinoma		√ (11/2015)				
Hepatocellular carcinoma		√ (9/2017)				
Classical Hodgkin’ s lymphoma		√ (5/2016)	√ (3/2017)			
Head & neck squamous cell carcinoma4		√ (11/2017)	√ (8/2016)			
Urothelial Carcinoma		√ (2/2017)	√ (5/2017)	√*(5/2016)	√ (5/2017)	√*(5/2017)
Colorectal cancer with high msi/mrd		√ (8/2017)				
Gastric cancer			√ (9/2017)			
Solid tumor with high msi/mrd			√ (7/2017)			
Merkel-cell carcinoma					√* (3/2017)	
Bladder Cancer				√ (04/2017)		

*First indication approval date by FDA

Use of ICIs brings forth a new toxicity called immune-related Adverse Events (irAEs) whose mechanisms and manifestations are quite different from those of cytotoxic chemotherapy, radiation or molecularly targeted agents. These irAEs are inflammatory in nature with potential to affect multiple organ systems. While not occurring in all patients, they are believed to be an autoimmune response that results from the blocking of the normal immune regulatory pathways. Common ICI irAEs include colitis, hepatitis, pneumonitis, dermatitis and endocrinopathies.

While non-endocrine irAEs require cessation of immunotherapy and usually resolve with immunosuppressive therapy [1–6], endocrine irAEs, if managed appropriately, do not require cessation of ICIs and are, for the most part, irreversible and require long term management [7, 8]. Endocrinologists will often be consulted to co-manage these patients and should be familiar with these endocrine irAEs (called ICI-induced endocrinopathies in this paper).

A drug’s AE profile may change during its life cycle. Marketed drugs may have previously unreported AEs in real world patients because these patients do not have to meet study entry criteria of clinical trials. In addition, marketed drugs may have rare AEs which surface when many more patients are treated with them. In the United States (US), AEs of marketed drugs are reported by healthcare providers, patients and others directly or indirectly to the Food and Drug Administration (FDA). Healthcare providers may also publish their patient’s AEs as case reports in medical journals. Such publications contribute to post-marketing safety surveillance of drugs and alert clinicians to these possible drug AEs.

A 2016 systematic review of case reports of irAEs related to CTLA-4 and PD-1 blockade therapy [9] was based on a literature search done in August 2015, four years after the approval of anti-CTLA-4 mAB (ipilimumab) in 2011 and a few months after the approval of anti-PD-1 mAbs (nivolumab and pembrolizumab) in 2014. It included 84 cases of ICI-induced-endocrinopathies – 79 with ipilimumab, 2 with pembrolizumab and 3 with nivolumab.

Since that review [9], the profiles of ICI-induced endocrinopathies have changed for various reasons: (a) many more advanced cancer patients have been treated with ICIs; (b) the ICIs have been marketed longer; (c) more cancers have been approved for treatment with ICIs; (d) a new subclass of ICI drugs (anti PDL-1 mAbs) was approved in 2016/2017; and (e) rare cases of ICI-induced endocrinopathies have been reported as more patients are treated with these drugs.

In this paper, we report the findings of a scoping review of ICI-induced endocrinopathies case reports based on a literature search conducted in July 2017, 6 years after the approval of ipilimumab, 3 years after the approval of nivolumab and pembrolizumab, and about a year after the approval of atezolimumab, avelumab and durvalumab. We aim to uncover new knowledge of the changing profiles of ICI-induced endocrinopathies, before and after marketing, as more real-world patients with different advanced cancers are treated with these drugs.

## Method

We used the framework proposed by Arksey and O’Malley [10] and modified by Levac et al. [11] to conduct this scoping review to map out the ICI-induced endocrinopathies.

### Stage 1: Identifying the research questions – Purpose

What is the spectrum (extent, range and nature) of ICI-induced endocrinopathies? How and when did they present clinically? What investigations were conducted to support their diagnoses? How were they managed at onset and what were their outcomes?

### Stage 2: Identifying relevant studies- data sources and searches

In July 2017, we searched 4 citation databases for published articles and abstracts of cases of endocrinopathies associated with ICI therapy for cancer. The search was initially developed in 3 Ovid MEDLINE databases (MEDLINE, In-Process & Other Non-Indexed Citations, Epub Ahead of Print) and then optimized for Embase.com, Cochrane Central Register of Controlled Trials via Wiley Online Library, and Clarivate Analytics Web of Science. The searches were built with a combination of controlled terms (Medical Subject Headings and EMTREE when available), and title or abstracts keywords. No date or language restrictions were included in the searches, but animal studies, editorials, and comments were excluded from review.

Throughout the review process until January 31, 2018 the authors conducted hand-searches of reference lists in articles, reviewed the contents of relevant journals, and ran supplemental searches in Ovid MEDLINE, Embase, and the Cochrane Central Register to capture recently published reports. Duplicate citations were excluded in EndNote X6 (Clarivate Analytics). (All reproducible search strategies are available in Additional file 1: Appendix 1).

### Stage 3: Study selection

For eligibility screening, the citations were categorized by type of endocrinopathy. For this review, a case should have information on the type of cancer, type of ICI used, clinical presentation, biochemical tests’ results, imaging results (if done), and initial and follow-up treatment.

### Stage 4: Charting the data – Data extraction

The citations were distributed to teams of at least 2 endocrinologists to review (pituitary, adrenal and parathyroid, and diabetes insipidus: MHT and LYS; thyroid: RI and MHT; Diabetes mellitus: KMS, RG and MHT). Using a 2-step approach, each team member reviewed the title and abstract to identify potentially eligible relevant citation. Then, the full paper, if available, was retrieved, reviewed and data extracted. Non-English articles were translated with Google Translate.

The reviewers screened all assigned citations independently using a standardized data extraction form (Additional file 2: Appendix 2) to collect data on each case according to the guidelines for AEs publication as recommended by the International Society of Pharmaco-epidemiology and the International Society of Pharmacovigilance to evaluate the quality of case reports [12]. They then compared their decisions and resolved any differences by consensus.

### Stage 5: Collating, summarizing and reporting the results

For this review, a case should have information on the type of cancer, type of ICI used, clinical presentation, biochemical tests’ results, imaging results (if done), and initial and follow-up treatment. When all the above is not reported, the case should have information on 2 of the following: clinical, biochemical and treatment information. To imply association, there should be close temporal relationship between ICI therapy initiation and subsequent development of endocrinopathy. We summarized and categorized the identified ICI-induced endocrinopathies according to the endocrine gland affected, whether it was associated with hyper- or hypofunction of the gland, and whether it involved one or more endocrinopathies (polyglandular endocrinopathies).

## Results

**Fig. 1:**
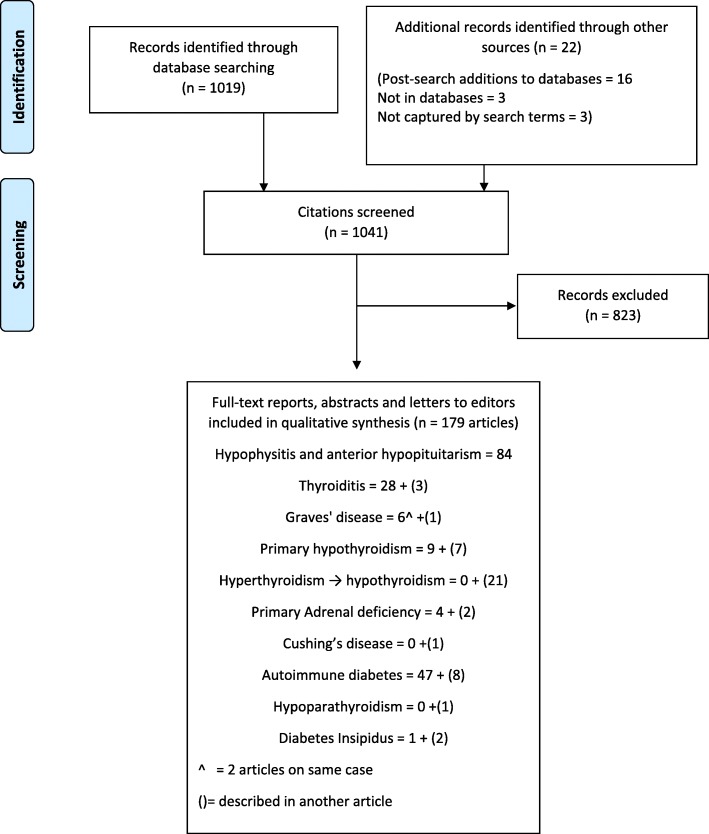
PRISMA flow diagram

Figure 1 shows the outcomes of the literature search. Of the 1041 citations identified, 218 had ICI-induced endocrinopathies citation with cases with sufficient information for this review. Some of these articles had information on two or more endocrinopathies. Eliminating these duplicates left 179 unique articles (116 papers, 43 abstracts and 20 letters to editor) which reported 451 cases of ICI-induced endocrinopathies that met study criteria for diagnosing the case.

Five endocrine glands are affected with 12 endocrinopathies:

- pituitary (hypopituitarism (multiple or isolated hormone deficiency[ies]), diabetes insipidus, and ACTH-dependent Cushing’s syndrome);
- thyroid (thyrotoxicosis [Graves’ disease and thyroiditis] and hypothyroidism [primary hypothyroidism and thyrotoxicosis progressing to hypothyroidism]),
- adrenal (primary adrenal insufficiency),
- pancreas (type 1 diabetes mellitus (T1DM) and type 2 diabetes mellitus (T2DM)), and
- parathyroid glands (primary hypoparathyroidism)

### Hypophysitis/anterior hypopituitarism

Table 2 summarizes the data from the 222 cases of hypophysitis/anterior hypopituitarism [13–96]. (Details of each case are in Additional file 3: Appendix 3). Of the 222 cases, 220 met the inclusion criteria; the 2 without biochemical tests’ results had clinical and treatment information.

**Table 2:** Cases of immune checkpoint inhibitors-induced hypophysitis and anterior pituitary insufficiency

Variable	Information
1. Number of Reports	84
2. Cases	222
3. Gender	Males = 145; Females = 62; Not reported = 15
4. Age (years)	Median = 61; Mean + SD = 60.4 + 11.4; Range = 31–85
5. Pertinent medical history	PHxEndoD: Reported = 12; Not reported = 210 FHxEndoD: Reported = 2; Not reported = 220 PHxAutoD: Reported = 4; Not reported = 218
6. Type of cancer	Melanoma = 193; Renal cell carcinoma = 6; Prostate carcinoma = 7; non-small cell lung cancer = 5, lung cancer = 5; papillary thyroid carcinoma =1; mesothelioma =1; not reported = 4
7. Check point inhibitor ICI Drug D/C?:	Ipilimumab = 188; Nivolumab = 13; Tremelilumab = 4; CTLA-4 = 8; Atezolizumab = 2; Ipilimumab + Nivolumab = 3; Nivolumab, then Ipilimumab = 1; Ipilimumab, then Pembrolizumab = 2; Ipilimumab, then Nivomumab = 1. Yes = 45 No = 17 Not reported = 160
8. Clinical presentation	Reflects the hormone(s) affected
9. Onset (weeks) after first dose	Median = 12; Mean = 13.8 + 10.3; Range 3–76
10. Biochemical tests	↓ACTH/cortisol = 183; ↓TSH/FT4/FT3 = 172; ↓LH/FSH/T/Estradiol = 137; ↓PRL = 22; ↓GH/IGF-1 = 22. ↑TSH, ↓FT4,/FT3 = 2
11. Diagnosis (patients with # hormone deficiencies[def])	5 deficiency (def) = 7; 4 def = 14; 3 def = 72; 2 def = 47; One (isolated) def = 36 (31 ACTH, 4 TSH, 1 LH, FSH def). 3 reports with case series account for remainder
12. Imaging Brain/Pituitary MRI/CT	Enlarged/Enhanced = 108; Normal 49; Not reported =11; Not done = 11; Sella abnormality = 3; atrophy = 3 3 reports with case series account for remainder
13. CTCAE grade reported	Yes = 5; Not reported in 217
14. Therapy at onset at diagnosis	High dose steroids = 144; steroids = 62; no steroids = 7; Not reported = 9
15. Outcome	219 recovered/discharged (replacement therapy 195 & 24 not reported); 3 deceased.

This cohort’s median age was 61 years. Most (65%) were male (in contrast to mostly females in non-ICI induced autoimmune hypophysitis). The majority (87%) had melanoma. CTLA-4 and PD-1/PD-L1 mAbs were used as monotherapy in 200 and 15 patients respectively with 7 on both. The median onset of clinical presentation was 12 weeks (range 3–76 weeks) after initiation of ICI. Symptoms reflected the associated hormone(s) deficiency(ies) with or without the mass-effect of pituitary enlargement (e.g. headache). In the 176 patients with detailed hormone information, 36 had one hormone deficiency, 47 had two, 72 had three, 14 with four and 7 with five. Secondary adrenal deficiency was present in 83%, secondary hypothyroidism in 77%, and secondary hypogonadism in 53% of patients. Of the 167 patients with pituitary magnetic resonance imaging (MRI)/computerized tomography (CT) scans, 108 showed enlarged/enhanced pituitary. Not all patients with enlarged pituitary presented with headaches and not all who presented with headaches (*n* = 126) had enlarged pituitary. High dose steroid was used as initial therapy in 69%, physiological dose steroid in 29% and no steroid in 2% of patients. When the cases were published, 220 were alive and 2 diseased (cause of death not stated). Of the 220, 184 were on replacement therapy, 32 had no information on discharge medications, and 4 on no replacement medications. During the preapproval and post-marketing periods there were 18 and 204 (166 in the first 4 years of marketing) respectively. There were 9 cases of polyglandular endocrinopathies involving anterior hypopituitarism - plus thyroiditis [31, 45, 89], plus primary hypothyroidism [60, 82, 90, 93], plus Graves’ disease and T1DM [76], and plus T1DM [95].

### Thyroid diseases

Table 3 summarizes the data from the 152 cases of ICI-induced thyroid disorders (Details of each case [31, 60, 76, 80, 82, 90, 97–138] are in Additional file 4: Appendix 4).

**Table 3:** Cases of immune checkpoint inhibitors-induced thyrotoxicosis and hypothyroidism

Variable	Thyrotoxicosis		Hypothyroidism	
	Graves’ disease	Thyroiditis	Primary hypothyroidism	Thyrotoxicosis → Hypothyroidism
Reports	7 (2 on same case)	31	16	21
Cases	6	67	29	50
Gender	Males = 4; Females = 2	Males = 27; Females = 27; Not reported =13	Males = 12; Females = 11; Not reported = 6	Males = 23; Females = 18; Not reported =9
Age (years)	Median = 54.5; Mean + SD = 57.3 + 7.3 Range = 51–67	Median = 61 Mean + SD = 62.1 + 12.8 Range = 24–88	Median = 64 Mean + SD = 64.1 + 7.9 Range = 46–85	Median = 63 Mean + SD = 64.1 + 10.4 Range = 42–86
Pertinent medical history	PHx + FHx of ThyD Reported = 2; Not reported = 4 PHx + FHx of AutoD Not reported = 6	PHx ThyD: Reported 4; Not reported 63. FHx ThyD: Reported 3. Not reported 64. HxAutoD: Reported 2; Not reported 65	PHx ThyD: Reported 5; Not Reported 24 FHx ThyD: Reported 0; Not reported 29 HxAutoD: Reported 1; Not reported 28	PHx ThyD: Reported 13; Not reported 37. FHx ThyD. Reported 2; Not reported 48. Hx AutoD: Reported = 1; Not reported 49.
Type of cancer	Melanoma = 6	Melanoma = 39, NSCLC =9; Breast Ca =1; Lung Ca = 5; RCC = 4; Multiple myeloma = 1; Esophageal Ca = 1; Not reported = 7	Melanoma = 18; Jaw Sq Ca = 1; Lung Sq Ca =6; RCC = 2; Bladder Ca = 1; Multiple myeloma = 1.	Melanoma = 29; NSCLC = 8; RCC = 4; Multiple myeloma = 1; breast CA = 1; Lung Ca = 3; Not reported =3.
Check point inhibitor(s)	Ipi = 4; Nivo + Ipi = 1, Tremeli = 1.	Ipi + bevacizumab = 2; Nivo = 23; Pembro =14; Anti-PD-1 = 6; Ipili = 6; Nivo + Ipili = 7; Ipili+Pembro =1; Ipili, then Pembro = 8.	Ipi = 2; PDL-1 = 1; Nivo = 15; Ipi, then Pembro = 9; Nivo+Ipi =2.	Nivo = 17; Pembro = 13; Anti-PD-1 = 6; Ipili = 2; Nivo+Ipili = 7; Ipili then, Pembro = 5.
ICI Drug D/C?	Yes = 3; Not reported =3	Yes = 12; No = 15; Not reported = 40	Yes =8; No = 5; Not reported = 16	Yes = 8; No = 14; Not reported = 28
Clinical presentation	2 with Graves’ Eye disease (GED) 4 with thyrotoxicosis	Hyperthyroidism symptoms = 43; Asymptomatic = 11; Not reported =13	Hypothyroid symptoms = 14; Asymptomatic = 3; Not reported =12. DKA = 2; cardiac = 2; adrenal insufficiency = 2	Many cases do not describe symptoms of hypothyroidism in contrast to symptoms of hyperthyroidism.
Onset after first dose (weeks)	Median = 7 Mean + SD = 75.8 + 166.8 Range 6–416	Median = 6 Mean + SD = 7.2 + 5.6 Range 3–28	Median = 12 Mena + SD = 14.2 + 7.1 Range 7–36	Median = 6 Mean + SD = 7.2 + 3.4 Range 3–15
Biochemical tests	All have +ve TRAb/TSI; 4 have +ve TPO/TG Ab 2 have NL TSH, FT4 (Graves’ Eye Disease) 4 ↓TSH,↑FT4, ↑FT3	All had ↓TSH, ↑FT4 &/FT3. 47 had reported TPO/TG Ab; 20 + ve. 22 pts. had reported TRAb/TSI; all –ve.	All had ↑TSH, ↓FT4. TPO/TG Ab reported in 14: all +ve. 4 had ↓ACTH & cortisol. 2 had ↑Glu, +ve GAD65 Ab, & ↓ C-peptide, pH. & ↑A1c	All had ↓TSH, 46↑FT4 /FT3 initially, 3 NL FT4 19 cases had ↑TSH &↓FT4/ FT3. TPO/TG Ab reported in 28: 19 + ve; TRAb/TSI in 21:1 + ve.33 cases stated just as hypothyroid.
Diagnosis	2 Graves’ eye disease 4 Graves’ disease	3 cases described as “thyroid storm”. 44 had symptoms of hyperthyroidism. 9 were asymptomatic. 11 symptoms not reported	Primary hypothyroidism (biochemically) in all. 14 had symptoms of hypothyroidism.	19 cases had primary hypothyroidism with ↑TSH & ↓FT4/FT3. Others stated to be hypothyroid with no tests results.
Imaging MRI, RAI uptake	2 enlarged extraocular muscles; 1 ↑thyroid perfusion,1 ↑RAI uptake. 1 MRI pituitary NL; 1 Not reported.	11 had ↓uptake on thyroid scan; 2 had ↑uptake on PET scan; 14 had thyroid US various findings; 31 not reported.	Thyroid US in 7–1 swollen gland, 6 heterogeneous echo-structure. PET scan in 5 (↑uptake in 3). Not reported in 16.	Not reported during hypothyroid state.
CTCAE grade reported	Not reported in all;.	Grade 2 = 1 and not reported. = 66	Not reported in all 29.	Grade 2 in one; Not reported in 48.
Therapy at onset at diagnosis	3 high dose steroids; 4 had anti-thyroid drugs(1 had LT4)	36 no treatment for hyperthyroidism; 8 on steroid (1 colitis; 3 adrenal insufficiency; 4 not reported); 16 on β-blockers; 8 had anti-thyroid drugs; 3 had iodine solutions (thyroid storm); 3 not reported.	LT4 = 26; No LT4 = 1; LT4 Not reported in 2. Steroids = 3; Insuli*n* = 2.	39 on LT4. 11 not reported.
Outcome	All improved; 2 on LT4 (1 had thyroidectomy; 1 became hypothyroid).	42 required LT4; 17 no LT4; 8 not reported.	23 0n LT4; 3 subclinical hypothyroid; 2 not reported; 1 deceased.	44 on LT4. LT4 not reported in 6.

### Thyrotoxicosis

Of the 73 thyrotoxicosis cases, 6 had Graves’ disease and 67 had thyroiditis. All had decreased thyroid stimulating hormone (TSH) and elevated free thyroxine (FT4)/free triiodothyronine (FT3) in addition to the clinical and treatment information. Those with Graves’ disease also had positive thyrotropin receptor Ab (TRAb) or Thyroid Stimulating Immunoglobulin (TSI). Both groups may or may not have positive thyroid peroxidase (TPO) and/or thyroglobulin (TG) Ab. For those who had radioisotope thyroid scans, Graves’ disease patients had increased uptake and thyroiditis patients had decreased uptake.

#### Graves’ disease

This cohort’s median age was 54∙5 years. Four of the 6 patients were male. All had metastatic melanoma. Ipilimumab was used in 5/6 patients, 4 as monotherapy and 1 with Nivolumab. The median onset of clinical presentation was 7 weeks after initiation of ICI (range = 6–416). Symptoms ranged from Graves’ eye disease [97, 98] to hyperthyroidism. High dose steroid was used as initial therapy in 3 cases and anti-thyroid drugs in 4 cases. All improved and 2 were on replacement thyroxine (1 had thyroidectomy and the other became hypothyroid). Two cases were reported before ipilimumab approval and 3 post-marketing. One case used tremelimumab which has not been approved yet. One case of polyglandular endocrinopathy had Graves’ disease followed by T1DM and anterior hypopituitarism [76].

#### Thyroiditis

This cohort’s median age was 61 years. In those with identified gender, 53% were female. The majority (61%) had metastatic melanoma. Anti-PD-1 mAbs were used in 60/67 patients, monotherapy in 44 and with/after Ipilimumab in 8 and 8 respectively. The median onset of symptoms was 6 weeks after initiation of ICI (range = 3–28). Clinical presentation ranged from no symptoms to thyroid storm [105, 106, 110]. High dose steroid was used as initial therapy in 6 patients (2 of these cases had concomitant adrenal insufficiency), anti-thyroid drugs in 6, β-blockers in 15 and iodide solution in 3. Of the 67 patients with follow-up information, 46 needed thyroxine replacement. During the pre-approval and post-marketing periods there were 2 and 65 reported respectively. There were 7 cases of polyglandular endocrinopathies involving thyroiditis - plus anterior hypopituitarism [31, 123, 126]; plus primary adrenal insufficiency [126]; plus pituitary adrenocorticotrophic hormone (ACTH)-dependent Cushing’s syndrome and anterior hypopituitarism [127]; plus hypoparathyroidism [122]; and plus type 1 diabetes mellitus [128, 130].

### Hypothyroidism

Of the 79 cases of hypothyroidism, 29 had primary hypothyroidism and 50 had hypothyroidism preceded by transient hyperthyroidism. Primary hypothyroidism cases had elevated TSH and decreased FT4/FT3. In those with hyperthyroidism progressing to hypothyroidism, TSH (when reported) was high in 19 cases. The remaining 31 were stated to have hypothyroidism with clinical and/or treatment information.

#### Primary hypothyroidism

This cohort’s median age was 64 years. In those with identified gender, 52% were male. Sixty-two percent had metastatic melanoma. As monotherapy anti-PD-1/PDL-1 mAbs were used in 16; with ipilimumab 11 (in sequence in 9 and combined 2) and ipilimumab monotherapy 2. The median onset of symptoms was 12 weeks after ICI initiation (range = 7–36). Symptoms ranged from mild to severe myxedema coma [133]. Levothyroxine (LT4) was started in 26, no LT4 in 1 and 2 not reported. Steroids were used as initial therapy in 3 because of concomitant adrenal insufficiency and insulin was used in 2 to treat diabetes mellitus. In follow-up, 23 needed LT4 replacement, 5 not reported, and I died (cause unknown). During the pre-approval and post-marketing periods 1 and 28 cases were reported respectively. There were 6 cases of polyglandular endocrinopathies involving primary hypothyroidism - plus anterior hypopituitarism [60, 82, 90, 93] and plus diabetes mellitus [132, 134].

#### Thyrotoxicosis progressing to hypothyroidism

This cohort’s median age was 63 years. In those with identified gender, 56% were male. The majority (59%) had melanoma. Anti-PD-1/ PDL-1 mAbs were used in 48 patients - monotherapy in 36, after ipilimumab in 5 and in combination with ipilimumab in 7. The median onset of symptoms was 6 weeks after hyperthyroidism (range 3–15). Many patients were stated to be hypothyroid without clinical information on symptoms. At onset of symptoms 39 patients were prescribed LT4. In follow-up, 44 needed LT4 replacement, and LT4 replacement was not reported in 6 patients. All 50 cases were reported in post-marketing period. There were 7 cases of polyglandular endocrinopathies involving hyperthyroidism progressing to hypothyroidism - plus anterior hypopituitarism [76, 123];plus primary adrenal insufficiency (PAI) [126]; plus pituitary ACTH-dependent Cushing’s syndrome [127]; plus hypoparathyroidism [122]; and plus T1DM [76, 128].

### Diabetes mellitus

Table 4 summarizes the data from the 66 cases of ICI-induced diabetes mellitus. (Details of each case [76, 127, 128, 130, 139–185] are in Additional file 5: Appendix 5). All met the inclusion criteria.

**Table 4:** Cases of Immune Checkpoint Inhibitors Induced Diabetes Mellitus

Variable	Information
Reports	55
Cases	66
Gender	Males = 42; Females = 24
Age (years)	Median = 63.5; Mean ± SD = 62 ± 13.2; Range = 28–84
Pertinent medical history	PHx diabetes: Reported = 36; Not reported = 30 FHx diabetes: Reported 27; Not reported = 39 Hx AutoD: Reported = 15; Not reported = 51
Type of cancer (all metastatic)	Melanoma = 30; Non-small cell lung cancer = 10; Renal cell carcinoma = 7; Lung cancer = 11; Urothelial carcinoma = 2, Small cell jaw cancer = 1, Small cell tonsillar cancer = 1; Small cell maxillary sinus cancer = 1; Hodgkin’s lymphoma = 1, Cholangiocarcinoma = 1, Not reported = 1
Check point inhibitor Drug D/C?:	Pembro = 13; Nivo = 32; Nivo + Ipi = 6; Pembro + Ipi = 1; Ipi, then Pembro = 5; Ipi, then Nivo = 3; Avelu + Utomi = 1; Atezo = 2; Unnamed PDL-1 = 2; Ipi = 1 Yes = 22, No = 15, Ipi stopped/Pembro continued = 1, Not reported = 28
Clinical presentation	DKA = 43; Hyperglycemia = 20; Not reported = 3
Onset (weeks) after first dose	Median = 7, Mean ± SD = 11.7 ± 11.7 Range 1–52
Biochemical tests	↑Glucose = 57; ↑A1c = 49; ↑β-OHB = 10 ↓pH = 35; ↓C-pep = 48
Antibody evaluation	Any antibodies (GAD65, IA2, ZnT8, IAA) positive = 34 (51.5%) Antibodies negative = 27 (41%) Antibodies not reported = 5 (7.5%)
Pancreatic imaging	CT with ↓ volume or atrophy = 3, CT with pancreatitis = 1; MRI with diffuse inflammatio*n* = 1
CTCAE severity grade reported	Yes = 4; Not reported = 62
Therapy at onset at diagnosis in addition to insulin	Metformin =1, Glimepiride = 1,glyburide = 1; Diet = 2, High dose steroids* = 10
Outcome	Remained insulin-dependent = 52, Recovered = 1, Not reported = 13

*Treatment dose steroids as part of chemotherapy regimen (n = 2), treatment of other autoimmune manifestation (*n* = 3), to reverse autoimmune diabetes (n = 2), to decrease insulin resistance (n = 1); for adrenal insufficiency (n = 2)

Diagnosis of T1DM was based on clinical (onset, clinical symptoms, immediate and subsequent insulin therapy) and biochemical (glucose, ketone bodies, islet antibodies, acidotic state) data. Any case with positive islet antibody(ies) is diagnosed as T1DM based on the presumed underlying autoimmune nature. In cases where islet antibodies were negative or not reported, we diagnosed them to have T1DM based on their presentation with diabetic ketoacidosis (DKA) in a previously normoglycemic patient, new onset severe hyperglycemia with a low C-peptide in a patient without known diabetes, new insulin requirement in a patient with known T2DM; and anyone with “fulminant” T1DM.

This cohort’s median age was 63 years. Most (64%) were male and the majority (45%) had metastatic melanoma. The ICIs used were anti-PD-I mAbs (*n* = 45); anti-PD-L1 mAbs (*n* = 5); anti- PD-1 mAbs + anti-CTLA-4 mAb (*n* = 7); anti-CTLA-4 mAb then Anti-PD-1 mAbs (*n* = 8); anti CTLA-4 mAb (*n* = 1). The median onset of clinical presentation was 7.5 weeks after initiation of ICI (range = 1–52 weeks). Sixty-five had T1DM based on our diagnostic criteria (see above) and one probably had T2DM (had duodenopancreatectomy and did not require insulin to treat mild hyperglycemia) [178]. There were 4 cases with T2DM before ICI therapy and who developed T1DM based on a new insulin requirement when on ICI [132, 149, 171].

In the T1DM cohort, 43 presented in DKA and 20 with marked hyperglycemia. In the DKA group 12, mostly from Japan, were described to have fulminant T1DM [130, 139, 147, 148, 152–157, 165, 168–170]. Islet-related Abs (Glutamic acid decarboxylase 65(GAD 65), Islet Antigen 2 (IA2), Insulin auto-antibody (IAA) or Zinc Transporter 8 (ZnT8) Ab were positive in 51.5% (*n* = 34), negative in 41% (*n* = 27) and not reported in 7.5% (*n* = 5). At onset of clinical presentation, high dose steroid was used in 10, cases, insulin infusion in 60, oral glucose-lowering medications initially continued in 4, with 2 no information on treatment. All 66 patients recovered from their hyperglycemia with treatment - 53 remained insulin dependent and the outcomes of 13 were not reported. One patient, initially requiring insulin, discontinued insulin on day 81 after pembrolizumab was discontinued [142]. All the cases using anti-PD-1 mAbs were reported post-marketing, most 2–3 years after their approval in 2014. There were 6 cases of polyglandular endocrinopathies involving T1DM - plus anterior hypopituitarism [75, 95], plus anterior hypopituitarism + Graves’ disease [76], plus, thyroiditis [129, 130], plus primary hypothyroidism [132], and plus hyperthyroidism progressing to hypothyroidism [76, 130].

### Primary adrenal insufficiency, ACTH-dependent Cushing’s syndrome, hypoparathyroidism, and diabetes insipidus

Table 5 summarizes the data from 6 cases of PAI, 1 of pituitary ACTH-dependent Cushing’s syndrome, 1 of primary hypoparathyroidism and 3 of diabetes insipidus following ICI therapy. (Details of cases [16, 19, 34, 41, 122, 126, 127, 186–189] are in Additional file 6: Appendix 6). All met the criteria for inclusion in this review.

**Table 5:** Cases of Immune Checkpoint Inhibitors Induced Primary Adrenal Insufficiency, Cushing’s Disease, Hypoparathyroidism and Diabetes Insipidus

Variable	Primary adrenal insufficiency	ACTH-dependent Cushing’s syndrome	Hypoparathyroidism	Diabetes Insipidus
Reports No.	6	1	1	3
Cases No.	6	1	1	3
Gender	Male = 3; Female = 2; Not reported =1	Female = 1	Male = 1	Male = 3
Age (years)	Median = 52; Mean + SD = 51.2 + 4.7 Range 43–56	53	73	Median = 62; Mean + SD = 61.7 + 11.5 Range = 50–73
Pertinent medical history	PHx EndoD: NR = 6 FHx EndoD: NR = 6 HX AutoD: NR = 6	PHx EndoD: NR FHx EndoD: NR Hx AutoD: NR	PHx EndoD: NR FHx EndoD: NR Hx AutoD: No	PHx EndoD: NR = 3 FHx EndoD: NR = 3 Hx AutoD: NR = 3
Type of cancer (all metastatic)	Melanoma = 3; RCC = 1; Lung adenoCa = 1; NSCLC = 1	Melanoma	Melanoma	Prostate Ca = 1; Melanoma = 1; Merkel cell Ca = 1.
Check point inhibitor(s)	Ipilimumab =2; Nivolumab =3; Pembrolizumab =1	Ipilumumab + Nivolumab	Nivolumab +Ipilimumab	C1: Ipilimumab. C2: Ipilimumab C3: Avelumab
ICI Drug D/C?	Yes = 1; Not reported = 5	Yes = 1 at 12 wks.	Not reported =1	
Clinical presentation	Fatigue, weight loss, anorexia, nausea, vomiting, headache, etc	Anorexia, weakness	Paresthesia, weakness, etc	C1: Polydipsia, polyuria C2: Polydipsia, polyuria C3: Polydipsia, polyuria.
Onset (weeks) after 1st dose	Median = 10 Mean + SD =14.92 + 14 Range = 1.5–36	14 wks	6 wks	Median = 12; Mean + SD = 3 + 16; Range = 3–16
Biochemical tests	↑ACTH in 5; ↓cortisol in 4; Synactin test +ve & + ve adrenal Ab in 1 without cortisol. In 1 cosyntropin stimulation test was negative and pt. had enlarged adrenal glands preceded by secondary adrenal \insufficiency.	9wks: slight ↑ in cortisol; 12 wks: ↑ACTH, ↑cortisol, ↑[cortisol]_u._ Abnormal low dose dexamethasone suppression test. 16 wks: ↓ACTH, cortisol. 6wks: ↓TSH, ↑FT4, FT3. Then ↓TSH, FT4, FT3. ↓LH, estradiol. Normal PRL.	↓Calcium, undetectable PTH, ↑ phosphate_4_, ↓vitamin D, magnesium 7wks: ↓TSH, ↑FT4, FT3, then ↓TSH, FT4, FT3.	C1: ↓ACTH, cortisol, TSH, FT4, FT3, LH & FSH. Normal glucose. C2: ↓ACTH, cortisol, TSH, FT4, FT3, LH & FSH. Normal glucose. Water deprivation test: partial diabetes insipidus C3: ↑[sodium],[osmolality]_s_, ↓[osmolality]_u_. Normal glucose
Diagnosis	Primary adrenal insufficiency	Cushing’s disease, then 2^o^ adrenal insufficiency. Thyroiditis, then hypothyroidism. 2^o^ hypogonadism.	Primary hypoparathyroidism	Diabetes insipidus
Imaging MRI/CT	MRI brain: 2 normal & 2 enlarged pituitary. Abd CT scan: atrophied adrenals; enlarged in 1	MRI pituitary at 12 wks: enlarged	EKG: prolonged QT interval	MRI pituitary: Normal in all
CTCAE grade reported	Severity not reported in all:	Not reported	Not reported;	Not reported in all.
Therapy at onset at diagnosis	iv steroids in 4. Oral steroids in 2. Florinef in 2	Wk5: LT4 Wk16: HCT & LT4.	iv calcium gluconate, then oral vitamin D & Ca carbonate. LT4.	C1: High dose steroidsC2: HCT, LT4 & desmopressin. C3: Desmopressin
Outcome	5 discharged on steroids. 1 NR.	HCT & LT4.	Calcium carbonate + calcitriol LT4.	C1: NR. C2: HCT, LT4 & desmopressin; C3: Desmopressin D/C

#### Primary adrenal insufficiency [16, 34, 126, 186–188]

This cohort’s median age was 52 years. In those with identified gender, 60% were male. Of the 6 patients, 3 had metastatic melanoma. Anti-PD-1 mAbs were used in 4/6 patients as monotherapy. Ipilimumab was used in 2 patients. The median onset of symptoms was 10 weeks after ICI initiation (range = 1.5–36). High dose steroid was used as initial therapy in 4 and physiological steroids in 2 cases. All recovered clinically with treatment and were on replacement oral hydrocortisone with two [126, 186] also on fludrocortisone. The patient reported in [188] received iv prednisolone which contains some mineralocorticoid effect but at discharge fludrocortisones was not mentioned. One case [34] initially had secondary adrenal insufficiency (SAI) but was found to have enlarged adrenals at week 16 after ipilimumab initiation. When a cosyntropin stimulation test done then showed no cortisol response the authors concluded the patient also had PAI. A negative CST occurs in PAI but can also occur with SAI of significant duration.

#### Pituitary ACTH-dependent Cushing’s syndrome [127]

Onset of the hypercortisolism symptoms was 12 weeks after initiation of ipilimumab plus nivolumab for melanoma. Four weeks later, secondary adrenal insufficiency developed. Both these were preceded by transient hyperthyroidism in week 6 that progressed to secondary hypothyroidism in week 9 after the first dose of ICI.

#### Primary hypoparathyroidism [122]

This melanoma patient, treated with ipilimumab plus nivolumab, developed symptoms of acute hypocalcemia 6 weeks after the first dose of ICIs. On the 3rd day in hospital, he developed transient thyroiditis which progressed to hypothyroidism.

#### Diabetes insipidus (DI) [19, 41, 189]

Two patients were treated with ipilimumab and 1 with avelumab (the first case of DI following anti-PD-L1 therapy). The median onset of symptoms (polydipsia and polyuria with euglycemia) was 12 weeks after initiation of ICI. Two [41, 189] reported biochemical data in keeping with DI and these 2 were discharged on desmopressin.

### Reported endocrinopathies not supported by data

Two cases of hyponatremia were attributed to syndrome of inappropriate ADH secretion [22, 186]. However, both had secondary adrenal insufficiency that could have caused the hyponatremia.

The only case of hypercalcemia was associated with increased PTH-related peptide, not PTH; hence, it was not primary hyperparathyroidism [189].

## Discussion

### Clinical presentations

**Fig. 2:**
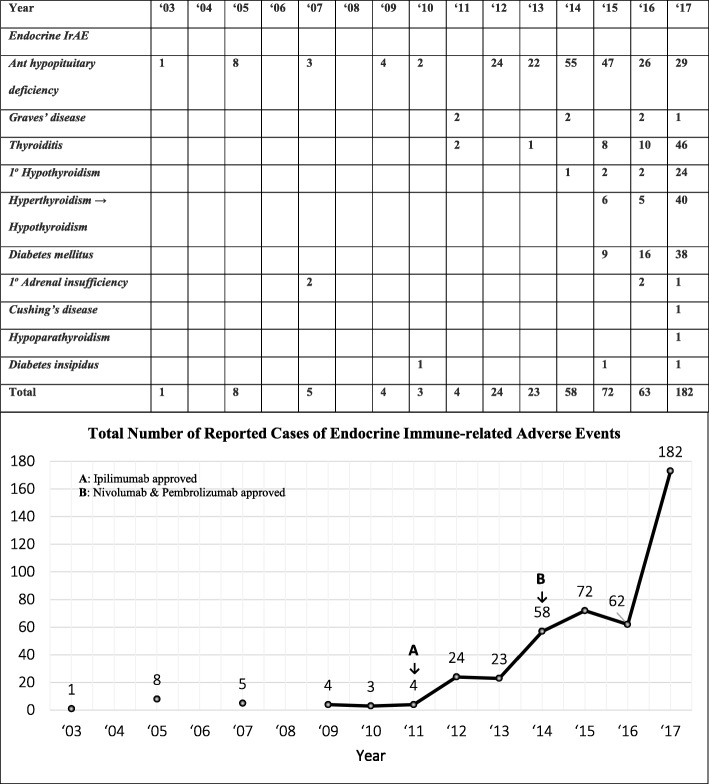
Reported cases of immune checkpoint inhibitor-induced endocrinopathies are increasing

Compared with the 2016 systematic review of case reports of irAEs, which included 84 cases of endocrine irAEs, in ICI treated cancer patients [9], our review shows a five-fold increase in case reports of endocrine irAEs (from 84 to 451). This increase most likely reflects wider use of ICI therapy in more cancer patients - (a) more melanoma patients (the original indication) are now treated with 2 classes of ICIs; (b) as new indications are approved for other cancers, more patients are now treated with ICIs (Table 1); and (c) more cases of anti-PD-1-induced endocrinopathies were reported in the past 3 years after their approval in 2014 (Fig. 2). During the 3–4-years period after approval of each class of ICI (ipilimumab in 2011 and nivolumab and pembrolizumab in 2014) a striking increase in cases of ICI-induced endocrinopathies were published with their use in the first 2–3 years post-marketing period (Fig. 2). With the introduction of anti-PD-L1 drugs in 2016 and 2017 for cancer patients, more cases ICI-induced endocrinopathies [190] will likely be reported in the coming 3–4 years.

**Fig. 3:**
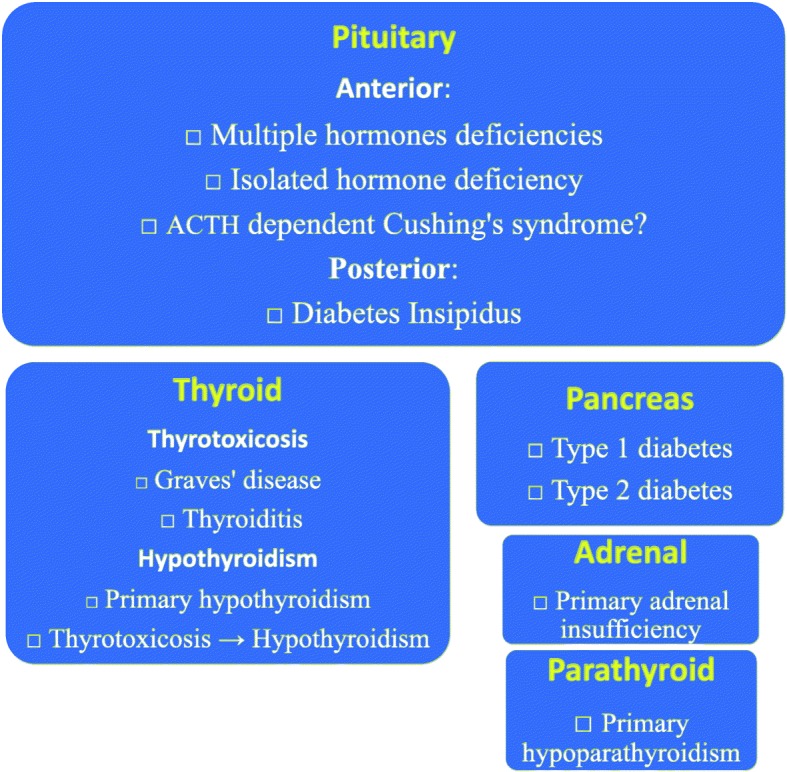
Spectrum of immune checkpoint inhibitor-induced endocrinopathies

In the 2016 review [9] ipilimumab was associated with 68 cases of hypophysitis, 4 cases of thyrotoxicosis, 4 cases of hypothyroidism, 1 case of syndrome of inappropriate secretion of antidiuretic hormone, 1 case of central adrenal insufficiency, 1 case of primary adrenal insufficiency; pembrolizumab was associated with 1 case of hypothyroidism and 1 case of diabetes mellitus; and nivolumab was associated with 2 cases of hypothyroidism. Our scoping study uncovers a wider spectrum of ICI-induced endocrinopathies - 222 hypopituitarism, 152 thyroid disorders, 66 diabetes mellitus, 6 primary adrenal insufficiency, 1 pituitary ACTH-dependent Cushing’s syndrome, 1 hypoparathyroidism and 3 diabetes insipidus cases (Fig. 3).

Clinically, endocrinopathies can present with symptoms of hormone deficiency, hormone excess or both (in the same or different glands). The spectrum of clinical presentation ranges from no symptoms [93, 111, 112, 116, 118, 135] (diagnosis made by biochemical tests) to severe, life-threatening symptoms - thyroid storm [105], myxedema coma [133] and diabetic ketoacidosis [144]. When symptomatic, the presentations of these ICI-induced endocrinopathies reflect the perturbations of hormone(s) produced by the affected gland(s).

Single hormone deficiency - ACTH in isolated ACTH deficiency [34, 45, 70, 86] or insulin in T1DM [132, 139, 140, 142]) are more commonly reported than single hormone excess - thyroxine in Graves’ disease [102] and ACTH in Cushing’s syndrome [127]. Occasionally, the endocrinopathy may present with symptoms of one hormone excess followed by symptoms that reflect the same hormone’s deficiency - hyperthyroidism progressing to hypothyroidism [116].

Multiple glands may be affected presenting as polyglandular endocrinopathies. The patient can present with symptoms and biochemical changes of two endocrine glands in sequence - thyroiditis and type 1 diabetes mellitus [130], hypoparathyroidism and thyroiditis [122], thyroiditis and ACTH-dependent Cushing’s syndrome [127], and primary hypothyroidism and isolated ACTH deficiency [93].

When the pituitary gland, which secretes 7 hormones, is affected, multiple hormonal deficiencies can occur simultaneously; then, the patient presents with symptoms of multiple pituitary hormones deficiencies [14]. In our review, various combinations of pituitary hormone deficiencies occurred, with 3 hormone deficiencies being the commonest. Finally, patients with hypophysitis are sick and their thyroid/gonadal axis may be suppressed in the acute phase of the disease.

Onset of symptoms can occur as early as 1 week [140] or as late as 416 weeks [102] with most in the first 20 weeks.

The pituitary ACTH-dependent Cushing’s syndrome [127] is the first case reported and needs to be confirmed. The etiology of the increased ACTH secretion may be due to ectopic Corticotropin-Releasing Hormone (CRH) production as expression of CRH has been reported in advanced melanoma cells [191] and Cushing’s syndrome due to ectopic CRH secretion has been reported [192]. As this case report’s authors did not investigate this, we cannot comment further but raised this as a possible etiology and point out that this patient’s Cushing’s syndrome and hypophysitis are unlikely to be related. Whether and how the ICIs stimulate the secretion of CRH remains to be reported.

High-dose systemic glucocorticoids are given as immunosuppressive to treat concomitant non-endocrine irABs (colitis, hepatitis, etc). For endocrine irABs, high-dose systemic glucocorticoids are given to treat adrenal crisis, hypophysitis and in selected cases of thyroiditis. Such treatment did not improve the outcome of ipilimumab-related hypophysitis in melanoma patients [8]. Recently, high-dose glucocorticoids treatment for ipilimumab-related hypophysitis in melanoma patients was associated with reduced survival [193]. High dose systemic glucocorticoids when given to diabetes patients (see Table 4) can worsen their hyperglycemia.

### Subclass of ICI and ICI-induced endocrinopathies

We found anti-CTLA-4 mAb to be the ICI most frequently associated with hypophysitis and anterior pituitary hormone(s) deficiencies (*n* = 188 as monotherapy) compared with Anti-PD-1 mAbs (*n* = 13). The differences observed above may reflect longer period of use as ipilimumab was approved in 2011, 3 years before nivolumab and pembrolizumab. Other reasons, including biology of endocrine glands, may explain this difference (see below).

We found anti-PD-1 mAbs are the ICIs more often associated (when compared with CTLA-4 Ab) with autoimmune thyroiditis (37 vs 6 as monotherapy), primary hypothyroidism (15 vs 2 as monotherapy), hyperthyroidism progressing to hypothyroidism (30 vs 2 as monotherapy) and type 1 diabetes mellitus (45 vs 1 as monotherapy). Of note, in our review, all 6 patients with Graves’ disease were treated with anti-CTLA-4 mAb (5 as monotherapy). A meta-analysis reported polymorphism of CTLA-4 can increase the risk for Graves’ disease [194] but how this interacts with ICI is unclear.

It is improper to calculate the frequency of each ICI-induced endocrinopathies in this review as we do not have the total number of cases that have been reported other than those that have been published. Cukier et al. reported frequencies of endocrine irAEs observed with immunotherapies [195]. Relevant to this review, the frequencies for endocrine irAEs for ipilimumab, nivolumab and pembrolizumab respectively are: hypophysitis 1.5–17, 0.6–1.5 and 0.6–1%; hypothyroidism 1.5–6.8, 9–10.8 and 7–9.1%; hyperthyroidism 4, 2.7 and 3.4–7.8%; primary adrenal insufficiency 0.8–1.6, 1% and not reported; Type 1 diabetes not reported, 0.9, and 0.2%. There are similarities in the frequencies of endocrinopathies associated with different ICIs reported by Cukier et al. [195] and those reported in this review.

### Pathogenesis

**Fig. 4:**
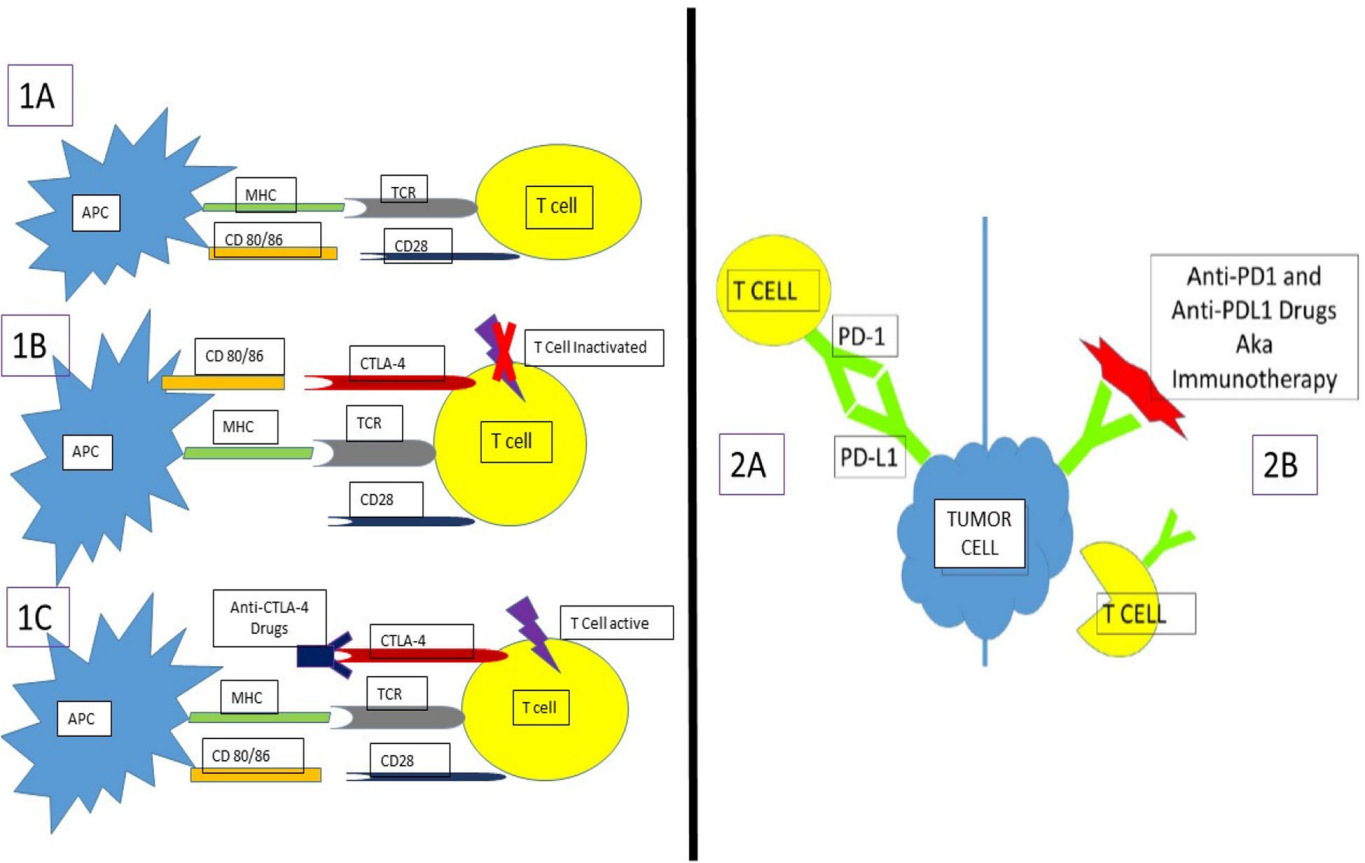
Mechanisms of Action of Anti-PD(L)1 and CTLA-4 Drugs. **1a** Normal activation state of T cells after interaction with Antigen presenting cell (APC). TCR = T-Cell receptor. **1b** Inhibition of T cell activation due to CTLA-4 out-competing CD28 for interaction with CD 80/86. **1c** Anti-CTLA-4 drug inhibits CTLA-4 thus CD28 is able to interact with CD80/86 and activate T cell. **2a** PD-L1 on tumor cell prevents T cell activation. **2b** PD-L1 is blocked by anti-PD(L)1 drugs, T cell activation against tumor is possible

How ICIs damage endocrine glands are unclear; however, several hypotheses to explain this have emerged [196]. All endocrinopathies associated with ICI therapy are hypothesized to be autoimmune in etiology. Checkpoint regulator proteins diminish the immune response to antigen and hence act as a brake on the immune system [1–6]. Blockade of these checkpoint regulator proteins releases this brake and allows the patient’s immune system to attack cancer cells as well as damage certain healthy tissues by autoimmune mechanisms (Fig. 4).

For hypophysitis, a mouse model of anti-CLTA-4-induced hypophysitis demonstrated that CTLA-4 is expressed in pituitary endocrine cells and, when blocked by administration of anti-CTLA-4 mAb, leads to site-specific deposition of complement components, pituitary infiltration and pituitary Ab formation. This study also evaluated patients with prostate cancer and melanoma treated with ipilimumab and showed that those who developed hypophysitis developed pituitary Abs that were not seen in the patients who did not develop hypophysitis [47]. CTLA-4 antigen expressed by pituitary endocrine glands in cancer patients treated with CTLA-4 blockade was also reported by Caturegli et al. [63].

Hypophysitis is less common among patients treated with anti-PD-1/PD-L1 drugs. Some have hypothesized this is because mAbs to nivolumab and pembrolizumab are in the immunoglobulin (IgG) 4 class [197], as opposed to ipilimumab, a CTLA-4 antibody of the IgG1 class, which can activate the classical complement pathway [47].

The mechanism behind anti-PD-1 induced thyroid dysfunction is unclear. However, the development of anti-thyroid antibodies after the initiation of anti-PD-1 therapy suggests these drugs may be modulating the autoimmune equilibrium and unmasking latent autoimmunity [119]. As many cases of ICI-induced hypothyroidism are preceded by a period of transient hyperthyroidism, the mechanism may be destructive thyroiditis with release of thyroid antigen and consequent secondary antibody production [111]. In our study’s thyroiditis cohort, of the 49 patients tested for TPO/TG Ab, only 18 had positive titers. In addition, in one patient the hypothyroidism was preceded by Graves’s disease [76].

In our study, in those autoimmune diabetes patients who were tested for islet-related Abs (GAD65, IA2, ZnT8, IAA) 51.5% had positive titers, 41% had negative titers and 7.5% not reported. Insulin deficiency with low C-peptide was noted in over 70% patients. Several case reports [130, 147, 148, 152, 176, 177] of autoimmune diabetes following ICI therapy reported the HLA type of their patients increased their risk for developing type 1 diabetes.

Many cases of diabetes following ICI therapy were reported in 2016 and 2017, after the anti-PD-1 drugs were approved in 2014. Prior to this, T1DM following ICI therapy was reported to be rare – (a) diabetes was not reported in a 2016 meta-analysis [198] (b) reported as 0.2–0.9% after anti-PD-1 and 0.1–0.3% after anti-PD-L1 therapy in 2017 [195], and (c) only 13 cases (0.2%) in the 2018 systematic review and meta-analysis [199]. Our study identified 65 case reports of T1DM, many more than identified in meta-analysis of clinical trials [199]. In our study, 12 T1DM cases were fulminant T1DM - acute, recent onset of severe hyperglycemia with ketoacidosis with negative islet-related Abs in general [200]. Recently Stamatouli et al. reported that insulin-dependent diabetes induced with checkpoint inhibitors have similarities and differences when compared with classic T1DM [201].

### Case documentation

Very few case reports in our review specified the severity of the endocrine irABs considered “required” when reporting adverse events [12]. However, based on the information in the case reports, most were Grade 2 or 3 with a few Grade 4 cases. Grading the severity of endocrinopathies can be helpful as some endocrine irABs are emergencies – DKA, adrenal crisis, thyroid storm and myxedema coma – and can be life threatening requiring urgent care. Similarly, the information on the personal and family history of the specific endocrine disorder or autoimmune disorder is sparse except for the DM cases. Finally, only 91/445 cases reported stopping the ICI drugs when the irAE occurred with insufficient information on whether they were transient or permanent. Of great interest is 1 patient with T1DM did not need insulin 81 days after pembrolizumab was discontinued [144].

### Limitations and strengths

This scoping review has limitations. The first is the variability of information in published case reports. Some did not have all the data identified as “required” or “highly desirable” [12] but had enough to meet the study criteria. There was variability in the diagnostic tests done, including their timing. In the hypophysitis cases the pituitary imaging may be delayed and not all had pre-treatment imaging for comparison. Secondly, although almost all had close temporal relationship, there were a few irAEs which occurred much later [45, 56, 94, 102, 144, 147]. Thirdly, although many cases reported were in full papers, 43 were abstracts as their full reports could not be located at the time of writing. Fourthly, our scoping study covered only published case reports mainly in post-marketing. Fifthly, our scoping review included case reports up to January 31, 2018. Since then other case reports have been published [193]. Finally, as pointed out in a systematic review [202], some reports on irAEs in clinical trials, as in our review, do not have all required information.

Our review has strengths: (a) It is comprehensive with data collected in a standardized way from before and after marketing published case reports and can uncover important irAEs not previously addressed. A 2018 meta-analysis [199] addressed only hypothyroidism, hyperthyroidism, hypophysitis, primary adrenal insufficiency and diabetes mellitus. (b) Using clinical reasoning, diagnoses were based on integrated clinical, biochemical and management documentation complemented by a temporal relationship between initiation of ICI and development of endocrinopathies [203]; (c) our study uncovered many more cases of ICI-induced T1DM than in a 2016 systematic review of irAEs case reports [9] and a 2018 meta-analysis of clinical trials data [199]; and (d) we report unique or first of a kind endocrinopathy [122, 127] not mentioned in prior systematic reviews and meta-analysis [199].

## Conclusion

In closing, this scoping review mapped the changing landscapes of ICI-induced endocrinopathies before and after marketing of 1 anti-CTL4–1 mAb (approved 2011), 2 anti-PD-1 mAbs (approved 2014) and 3 anti-PDL-1 mAbs (approved 2016/2017). In mostly real-world patients, we uncovered a spectrum of endocrinopathies – in 5 glands with 12 endocrinopathies that manifest with symptoms of hormone excess or deficiency.

Case reports can alert physicians of a drug’s AEs [12]. With increasing use of ICIs, physicians in many medical specialties (oncology, endocrinology, emergency medicine and primary care) should be vigilant for these ICI-induced endocrinopathies’ occurrence and be able to diagnose and treat them. “Cross talk” for collaborative care between oncologists and endocrinologists has been proposed [204]. It is beyond the scope of this paper to cover algorithms and guidelines for the diagnosis and management of ICI-induced endocrinopathies in cancer patients. Some oncology centers [205, 206], National Oncology Societies [207, 208] and Society of Endocrinology Emergency Guidance document [209] have published algorithms and clinical practice guidelines. The reporting of endocrine irAEs can be improved [12, 202]. Our findings can help physicians manage their patients and professional societies develop their clinical practice guidelines.

## Additional files

Additional file 1: **Appendix 1.** Literature Search. (DOCX 16 kb)

Additional file 2: **Appendix 2.** Standardized Case Form for Data Collection. (DOCX 13 kb)

Additional file 3: **Appendix 3.** Cases of Immune Checkpoint Inhibitor-Induced Endocrinopathies –Hypophysitis and Anterior Hypopituitarism. (DOCX 51 kb)

Additional file 4: **Appendix 4.** Cases of Immune Checkpoint Inhibitors-Induced Endocrinopathies – Thyroid Disorders: (a) Thyrotoxicosis (b) Hypothyroidism. (DOCX 48 kb)

Additional file 5: **Appendix 5.** Cases of Immune Checkpoint Inhibitors-Induced Endocrinopathies –Diabetes Mellitus. (DOCX 41 kb)

Additional file 6: **Appendix 6.** Cases of Immune Checkpoint Inhibitor-Induced Endocrinopathies – Primary Adrenal Insufficiency, Cushing’s Syndrome, Hypoparathyroidism, Diabetes Insipidus. (DOCX 22 kb)

## Abbreviations

A1c: Glycated hemoglobin
ACTH: Adrenocorticotropic hormone
ADH: Anti-diuretic hormone
Anti-CTLA-4: Anti-Cytotoxic T-lymphocyte-associated antigen-4
Anti-PD-1: Anti-programmed cell death protein-1
Anti-PDL-1: Anti-Programmed Death Ligand-1
Atezo: Atezolumab
AutoD: Autoimmune disease
Avelu: Avelumab
Ca: Carcinoma
C-pep: C-peptide
CRH: Corticotropin releasing hormone
CST: Cosyntropin stimulation test
CT: Computerized tomography
CTCAE: Common terminology criteria for adverse events
D/C: Discontinued
DI: Diabetes insipidus
DKA: Diabetic ketoacidosis
EKG: Electrocardiogram
EndoD: Endocrine disease
FHx: Family history
FSH: Follicle stimulating hormone
FT3: Free triiodothyronine
FT4: Free thyroxine
GAD 65: Glutamic acid decarboxylase 65
GH: Growth hormone
HCT: Hydrocortisone
HD steroids: High dose steroids
IA-2: Islet antigen 2
IAA: Insulin auto-antibody
IGF1: Insulin growth factor-1
IgG: Immunoglobulin
Ipi: Ipilimumab
LH: Luteinizing hormone
LT4: Levothyroxine
MRI: Magnetic resonance imaging
Nivo: Nivolumab
NL: Normal
NR: Not reported
NSCLC: Non-small cell lung cancer
PAI: Primary adrenal insufficiency
Pembro: Pembrolizumab
PET: Positron emission tomography
PHx: Personal history
PRL: Prolactin
PTH: Parahormone
RAI: Radioactive iodine
RCC: Renal cell carcinoma
Rx: Treatment
SAI: Secondary adrenal insufficiency
SD: Standard deviation
T: Testosterone
T1DM: Type 1 diabetes mellitus
T2DM: Type 2 diabetes mellitus
TCR: T-cell receptor
TG: Thyroglobulin Ab
ThyD: Thyroid disease
TPO: Thyroperoxidase antibodies (Ab)
TRAb: Thyroreceptor Ab
Treme: Tremelimumab
TSH: Thyroid stimulating hormone
TSI: Thyroid stimulating immunoglobulin
US: Ultrasound
Utomi: Utomilumab
ZnT8: Zinc Transporter 8
βOB: β-OH butyrate
