# Supplementary material for: Spectrum of immune checkpoint inhibitors-induced endocrinopathies in cancer patients: a scoping review of case reports
Source: Clin Diabetes Endocrinol. 2019 Jan 22;5:1. doi: 10.1186/s40842-018-0073-4 (PMC6343255; doi:10.1186/s40842-018-0073-4)
Supplement: Supplementary file 1 — Appendix 1. Literature Search. (DOCX 16 kb) [file 40842_2018_73_MOESM1_ESM.docx]

**Appendix 1 Literature Search**

**Ovid MEDLINE search strategy**

| Search # | Search Terms |
| --- | --- |
| 1 | exp endocrine system diseases/ or (acth or adrenocortic* or dmt1 or dm t1 or diabet* or endocrin* or grave* or hyperthyroid* or hypoadrenal* or hypophysit* or hypothyroid* or neuroendocrin* or parathyroid* or polyendocrino* or t1 dm or t1dm or thyroid* or thyrotoxic* or (adrenal adj2 (deficienc* or insufficienc*))).tw |
| 2 | (checkpoint* adj4 (block* or immun* or inhibit* or therap*)).tw. or (checkpoint*.tw. and (exp immunotherapy/ or exp *cell death/)) or (anti-pd-1 or anti-pd-L1 or atezolizumab or avelumab or bavencio or durvalumab or duvalumab or imfinzi or ipilimumab or keytruda* or lambrolizumab or nivolumab or opdivo or pembrolizumab or pidilizumab or strentarga or tecentriq or tremelimumab or yervoy).tw. or (checkpoint* and ((program* adj4 death*) or necrocytosis)).tw. or ((block* or inhibit*) adj3 (ctla-4 or ctla4 or pd1 or pd 1 or pdcd1* or pdL1 or pd-L1 or pd-L-1)).tw. or (checkpoint* and (ctla-4 or ctla4)).tw. |
| 3 | and/1-2 not (exp animals/ not exp humans/) not (editorial or comment).pt |

**Ovid MEDLINE; Ovid MEDLINE In-Process & Other Non-Indexed Citations; Ovid MEDLINE Epub Ahead of Print**

1. exp endocrine system diseases/ or (acth or adrenocortic* or dmt1 or dm t1 or diabet* or endocrin* or grave* or hyperthyroid* or hypoadrenal* or hypophysit* or hypothyroid* or neuroendocrin* or parathyroid* or polyendocrino* or t1 dm or t1dm or thyroid* or thyrotoxic* or (adrenal adj2 (deficienc* or insufficienc*))).tw

2. (checkpoint* adj4 (block* or immun* or inhibit* or therap*)).tw. or (checkpoint*.tw. and (exp immunotherapy/ or exp *cell death/)) or (anti-pd-1 or anti-pd-L1 or atezolizumab or avelumab or bavencio or durvalumab or duvalumab or imfinzi or ipilimumab or keytruda* or lambrolizumab or nivolumab or opdivo or pembrolizumab or pidilizumab or strentarga or tecentriq or tremelimumab or yervoy).tw. or (checkpoint* and ((program* adj4 death*) or necrocytosis)).tw. or ((block* or inhibit*) adj3 (ctla-4 or ctla4 or pd1 or pd 1 or pdcd1* or pdL1 or pd-L1 or pd-L-1)).tw. or (checkpoint* and (ctla-4 or ctla4)).tw.

3. and/1-2 not (exp animals/ not exp humans/) not (editorial or comment).pt

**Embase.com** (no conference abstracts)

#1 'endocrine disease'/exp/mj OR acth:ab,ti OR adrenocortic*:ab,ti OR dmt1:ab,ti OR 'dm t1':ab,ti OR diabet*:ab,ti OR endocrin*:ab,ti OR grave*:ab,ti OR hyperthyroid*:ab,ti OR hypoadrenal*:ab,ti OR hypophysit*:ab,ti OR hypothyroid*:ab,ti OR neuroendocrin*:ab,ti OR parathyroid*:ab,ti OR polyendocrino*:ab,ti OR 't1 dm':ab,ti OR t1dm:ab,ti OR thyroid*:ab,ti OR thyrotoxic*:ab,ti OR (adrenal NEAR/2 (deficienc* OR insufficienc*)):ab,ti

#2 'checkpoint kinase inhibitor'/exp OR (checkpoint* NEAR/4 (block* OR immun* OR inhibit* OR therap*)):ti,ab OR 'anti-pd-1':ti,ab OR 'anti-pd-l1':ti,ab OR atezolizumab:ti,ab OR avelumab:ti,ab OR bavencio:ti,ab OR durvalumab:ti,ab OR duvalumab:ti,ab OR imfinzi:ti,ab OR ipilimumab:ti,ab OR keytruda:ti,ab OR lambrolizumab:ti,ab OR nivolumab:ti,ab OR opdivo:ti,ab OR pembrolizumab:ti,ab OR pidilizumab:ti,ab OR strentarga:ti,ab OR tecentriq:ti,ab OR tremelimumab:ti,ab OR yervoy:ti,ab OR (checkpoint*:ti,ab AND ((program* NEAR/4 death*):ti,ab OR necrocytosis:ti,ab)) OR ((block* OR inhibit*) NEAR/2 (‘ctla-4’ OR ctla4 OR pd1 OR 'pd 1' OR pdcd1* OR pdl1 OR 'pd-l1' OR 'pd-l-1')):ab,ti OR (checkpoint* AND (‘ctla-4’ OR ctla4)):ti,ab

#3 #1 AND #2 NOT (note:it OR editorial:it OR ‘conference paper’:it OR ‘conference abstract’:it) NOT ([animals]/lim NOT [humans]/lim)

**Embase.com** (conference abstracts)

('endocrine disease'/exp/mj OR acth:ti OR adrenocortic*:ti OR dmt1:ti OR 'dm t1':ti OR diabet*:ti OR endocrin*:ti OR grave*:ti OR hyperthyroid*:ti OR hypoadrenal*:ti OR hypophysit*:ti OR hypothyroid*:ti OR neuroendocrin*:ti OR parathyroid*:ti OR polyendocrino*:ti OR 't1 dm':ti OR t1dm:ti OR thyroid*:ti OR thyrotoxic*:ti OR ((adrenal NEAR/1 (deficienc* OR insufficienc*)):ti)) AND ('checkpoint kinase inhibitor'/exp/mj OR ((checkpoint* NEAR/3 (block* OR immun* OR inhibit* OR therap*)):ti) OR atezolizumab:ti OR avelumab:ti OR bavencio:ti OR durvalumab:ti OR duvalumab:ti OR imfinzi:ti OR ipilimumab:ti OR keytruda:ti OR lambrolizumab:ti OR nivolumab:ti OR opdivo:ti OR pembrolizumab:ti OR pidilizumab:ti OR strentarga:ti OR tecentriq:ti OR tremelimumab:ti OR yervoy:ti) AND ('conference abstract':it OR 'conference paper':it)

**Cochrane Central Register of Controlled Trials**

1. [mh "endocrine system disease"] or (acth or adrenocortic* or dmt1 or "dm t1" or diabet* or endocrin* or grave* or hyperthyroid* or hypoadrenal* or hypophysit* or hypothyroid* or neuroendocrin* or parathyroid* or polyendocrino* or "t1 dm" or t1dm or thyroid* or thyrotoxic* or (adrenal NEAR/2 (deficienc* or insufficienc*))):ab,ti

2. (checkpoint* NEAR/4 (block* or immun* or inhibit* or therap*)):ab,ti or (checkpoint*:ab,ti and ([mh immunotherapy] or [mh "cell death"])) or ("anti-pd-1" or "anti-pd-L1" or atezolizumab or avelumab or bavencio or durvalumab or duvalumab or imfinzi or ipilimumab or keytruda* or lambrolizumab or nivolumab or opdivo or pembrolizumab or pidilizumab or strentarga or tecentriq or tremelimumab or yervoy):ab,ti or (checkpoint* and ((program* NEAR/4 death*) or necrocytosis)):ab,ti or ((block* or inhibit*) AND ("ctla-4" or ctla4 or pd1 or "pd 1" or pdcd1* or pdL1 or "pd-L1" or "pd-L-1")):ab,ti or (checkpoint* and ("ctla-4" or ctla4)):ti,ab

3. #1 and #2

**Web of Science**

1. TS=((checkpoint* NEAR/3 (block* OR inhibit* OR immun* OR therap*)) OR "anti-pd-1" OR atezolizumab OR avelumab OR bavencio OR durvalumab OR duvalumab OR imfinzi OR ipilimumab OR keytruda* OR lambrolizumab OR nivolumab OR opdivo OR pembrolizumab OR pidilizumab OR strentarga OR tecentriq OR tremelimumab OR yervoy OR (checkpoint* AND ((program* NEAR/4 death*) OR necrocytosis)) OR ((block* OR inhibit*) NEAR/3 (pd1 OR "pd 1" OR pdcd1* OR pdL1 OR "pd-L1" OR "pd-L-1")))

2. TS=((acth OR adrenocortic* OR dmt1 OR "dm t1" OR diabet* OR endocrin* OR grave* OR hyperthyroid* OR hypoadrenal* OR hypophysit* OR hypothyroid* OR neuroendocrin* OR parathyroid* OR polyendocrino* OR "t1 dm" OR t1dm OR thyroid* OR thyrotoxic* OR (adrenal NEAR/2 (deficienc* OR insufficienc*))))

3. #1 AND #2
