# Supplementary material for: Spectrum of immune checkpoint inhibitors-induced endocrinopathies in cancer patients: a scoping review of case reports
Source: Clin Diabetes Endocrinol. 2019 Jan 22;5:1. doi: 10.1186/s40842-018-0073-4 (PMC6343255; doi:10.1186/s40842-018-0073-4)
Supplement: Supplementary file 2 — Appendix 2. Standardized Case Form for Data Collection. (DOCX 13 kb) [file 40842_2018_73_MOESM2_ESM.docx]

**Appendix 2: Standardized Case Form for Data Collection**

**Case #** **Reviewer Initials:**

**Paper:** (Authors/Title/year/vol/pages)

**Titles+Abstract Number:**

**Pt Initials**: **Age:** **Gender:**

**Type of cancer:**

**Time of onset after beginning ChPt Rx:** weeks months

**Which ChPt Rx?** Please give dose if available

**PD-1 inhibitors:** Pembrolizumab (keytruda) Nivolumab (Opdivo)

**PD-L1 inhibitors:** Atezolizumab (Tecentriq) Avelumab(bavencio) Durvalumab(Imfinzi)

**CTLA-4** Ipilimumab(Yervoy)

**Endocrine diagnosis:** **Dx correct?** Yes No Maybe

**Clinical presentation:**

**CTCAE Grade**: 1/ 2/ 3/ 4/ 5:

**Pt’s other autoimmune diseases:**

**Fam Hx of autoimmune disease:**

**Fam Hx of endocrine disorders:**

**Pt’s other endocrine diseases:**

**Pt’s medications:**

**Investigations:**

**Lab:**

**Immunology:**

**Imaging:**

**Treatment initiated:**

**Pt’s Course:**

**Pt’s Outcomes:**

**Was drug stopped?** Yes No

**What happened when drug stopped?** Better No change Worse
