# Supplementary material for: Spectrum of immune checkpoint inhibitors-induced endocrinopathies in cancer patients: a scoping review of case reports
Source: Clin Diabetes Endocrinol. 2019 Jan 22;5:1. doi: 10.1186/s40842-018-0073-4 (PMC6343255; doi:10.1186/s40842-018-0073-4)
Supplement: Supplementary file 3 — Appendix 3. Cases of Immune Checkpoint Inhibitor-Induced Endocrinopathies –Hypophysitis and Anterior Hypopituitarism. (DOCX 51 kb) [file 40842_2018_73_MOESM3_ESM.docx]

**Appendix 3: Cases of Immune Checkpoint Inhibitor-Induced Endocrinopathies –**

**Hypophysitis and Anterior Hypopituitarism**

| **Authors (year) (reference)** | **Cancer/**  **(# of patients)** | **Age/gender**  **Pertinent Hx** | | **Drug/dose** | | **Clinical Sx/Onset after 1^st^ dose**  **Drug D/C?** | | **Laboratory tests** | | **Imaging**  **MRI Pituitary** | **CTCAEGrade** | | **Therapy** | | **Outcome** | |
| --- | --- | --- | --- | --- | --- | --- | --- | --- | --- | --- | --- | --- | --- | --- | --- | --- |
| Phan GQ et al (2003) (13) | Melanoma  (n=1) | 54/M  PHx, FHx of EndoD & AutoD NR | | Chemo, Surgery, then Ipi 3m/kg q 3wks. | | Personality change /15 wks  Drug D/C?: NR | | ↓ACTH, cortisol,  ↓TSH, FT4, FT3,  ↓T,PRL,GH | | Upper limits of normal | NR | | HCT, LT4, T, | | Recovered  HCT, LT4, T | |
| Blansfield JA et al (2005) (14) | Melanoma  (n=6) C1-2,5-8  Renal Cell Carcinoma  (n=2) C3-4 | C1:31/M; C2:56/M  C3:47/M; C4:56/M  C5 61/M; C6:43/M  C7:51/M; C8:48/M  PHx, FHx of EndoD & AutoD NR | | CTLA-4 3-9mg/kg q3wks | | Headache, fatigue, memory loss,↓libido, etc/ C1:12wks; C2:12wks; C3:9wks;  C4:12wks; C5:15wks;  C6:18; C7:18wks;  C8:24wks  Drug D/C?: Yes in all. | | In C1-8:  ↓ACTH, cortisol  ↓TSH, FT4  ↓T | | C:1,2,5-8: enlarged pit + suprasellar extension.  C3: enlarged  C4: empty sella | NR | | C1,2 &4: replacement hormones  C3,5-8: HD steroids followed by replacement hormones | | All had HCT, LT4, & T initially.  C4: LT4 & T D/C & HCT lowered  C5 & & HCT weaned off.  C5-8 had LT4 & T D/C. | |
| Shaw S et al (2007) (15) | Melanoma  (n=1) | 44/M  PHx, FHx of EndoD & AutoD NR | | Tremeli dose 15 mg/kg  Single dose | | Headache, fatigue ↓libido/  12 wks  Dose D/C?: Yes | | NL ACTH cortisol &.GH  ↓TSH, FT4,  ↓T, LH & PRL | | Enlarged pituitary | NR | | LT4 & T | | 12 m after event no replacement therapy | |
| Yang JC et al (2007) (16) | Renal Cell Ca  (n=2) | Age/gender NR  PHx, FHx of EndoD & AutoD NR | | Ipi 3mg/kg x3 doses | | Symptoms NR/ 12-15 wks  Drug D/C? : NR | | In both, ↓ACTH, cortisol  ↓TSH, T4  ↓T | | Enlarged in 1  NL in 1 | NR | | HCT  LT4  T | | Required HCT & LT4 but T stopped. | |
| Kaehler KC et al (2009) (17) | Melanoma  (n=1) | 60/M  PHx, FHx of EndoD & AutoD NR | | Ipi 10mg/kg q3w x4 doses | | Headache, ataxia, required HCT vertigo etc./ 21wks  Drug D/C?: NR | | ↓cortisol,  ↓TSH, FT4, FT3  ↓ T | | Enlarged pituitary | NR | | Iv HD steroids; LT4 initially . | | Discharged on HCT. LT4 D/C. | |
| Carpenter KJ et al (2009) (18) | Melanoma  (n=3) | C1: 70/M  C2: 44/M  C3: 46/F  PHx, FHx of EndoD & AutoD NR | | C1: Ipi 10mg/kg q2w  x3 doses  C2-3: Ipi 10mg/kg q3wks | | C1-3: Dizziness, anorexia & fatigue/C1: 6 wks; C2-3 9 wks  Drug D/C?: in C1 & 2: Yes;  C3: NR | | C1: ↓ACTH, cortisol  ↓TSH, TT4,  ↓T, LH, FSH  C2: NL ACTH, ↓cortisol,  ↓TSH and ↓T  C3: ↓cortisol, TSH, NL FSH | | C1-3  enlarged pituitary. | NR | | C1-2: HD steroid +LT4  C3: HD steroid taper | | Still on steroids + LT4 | |
| Dillard T et al (2010) (19) | Prostate Ca  (n=2) | C1: 67/M  C2: 50/M  PHx, FHx of EndoD & AutoD NR | | Chemo, interferon,  then Ipi 10mg/kg q4w x4 | | C1: Headache, anorexia, asthenia/C1: 4 wks, C2:12 wks  C 2 also had Diabetes insipidus ..  Drug D/C?: No in both | | In both, ↓ACTH + cortisol  ↓TSH + FT4  ↓LH + FSH  Low PRL + IGF1  C 2 also had abnormal ACTH stimulation test | | C 1:  Pituitary Enhanced Case C2:NL | NR | | HD steroids in both cases. | | NR | |
| Min L et al  (2012) (20) | Melanoma  (n=7) | C1:64/M  C2:44/M  C3:57/M  C4:55/M  C5:70/f  C6: 55/M  C7: 64/M  PHx of ThyD in 4pts (C1,2,5,6F)  Hx of EndoD & AutoD NR | | Ipi 10mg/kg q3wks | | Malaise, anorexic, lethargy / C1:6 wks  C2;12 wks  C3: 9 wks  C4: 8 wks  C5: 12 wks  C6: 12 wks  C7: 10 wks  Drug D/C?: No in C1&7; No in C2-6. | | C1: ↓ACTH, cortisol, TSH, IGF-1  C2: ↓ACTH, cortisol. ↓TSH, FT4, T, LH  C3: ↓ACTH, cortisol, TSH, FT4, T, IGF-1  C4: ↓ACTH, cortisol, TSH, FT4, T, LH, IGF-1.  C5: ↓ACTH, cortisol, TSH  C6: ↓ACTH, cortisol, TSH, T  C7: ↓ACTH, cortisol, | | MRI pituitary  C1: NL  C2: Nl  C3: NL  C4: Enlarged. NL after steroids in 1 m  C5: Enlarged. NL after steroids in 1 m  C6: NL  C7: NL | NR | | Steroids and if indicated LT4 & T | | Replacement steroids; LT4 & T when indicated  C5: diseased 1 month later. | |
| Andrew S et al (2012) (21) | Melanoma  (n=1) | 50/M  PHx, FHx of EndoD & AutoD NR | | Chemo, then  Ipi 10mg/kg q3wk  x4 doses, then q3m | | Fatigue, headaches, etc./9 wks  Drug D/C?: Yes | | NL TSH, ↓FT4 & FT3  NL ACTH & Cortisol initially. Two wks later ↓Cortisol  ↓T | | NR | NR | | LT4, T  HD steroids | | All 3 hormones replacement | |
| Barnard ZR et al (2012) (22) | Melanoma  (n=1) | 75/F  PHx, FHx of EndoD & AutoD NR | | Surgery, Chemo then  Ipi 3mg/kg  x3 doses q3-4 wks | | Headaches  Onset NR  Drug D/C?: NR | | ↓ACTH, cortisol  ↓TSH, low NL FT4.  ↓{Na+] | | Pituitary enlarged | NR | | iv HD steroids and then tapered off over a month | | Recovered. | |
| Juszczak A et al (2012) (23) | Melanoma  (n=1) | 54/M  PHx, FHx of EndoD & AutoD NR | | Surg & Chemo,.Then Ipi dose NR | | Headache, lethargy/9 wks  Drug D/C?: NR | | ↓cortisol,  ↓TSH, FT4,  ↓LH, FSH,T, PRL. NL IGF-1. | | Pituitary enlarged | NR | | PO HD steroids +  L-T4 | | Replacement therapy | |
| Carra T et al (2012) * (24) | Melanoma  (n=12) | Age/Gender NR  PHx, FHx of EndoD & AutoD NR | | Ipi dose 0.3/3/10 mg/kg . | | Headaches (n=11), asthenia (n=7) &↓libido (n=2) / 3 wks (n=2), 9 wks (n=6), 12 wks (n=4)  Drug D/C?: NR | | Adrenal axis 6 affected  Thyroidal axis 9 affected  Gonadal axis 7 affected | | Swelling in 5  Enhancement in 4 | NR | | Steroids 11 pts  LT4 4 pts | | Recovered in 1 wk in 8 pts. | |
| Erankl VG (2012)* (25) | Melanoma (n=1) | 76/M  PHx, FHx of EndoD & AutoD | | Surgery, radiation, chemo. Then, ipi 3mg/kg q3wks x4 doses. | | Headache, fatigue/ 20 wks.  Drug D/C?:NR | | ↓ACTH, cortisol and abnormal cosyntropin test.  ↓TSH, FT4 | | MRI pituitary: Enhanced | NR | | Physiological steroids  LT4 | | HCT & LT4 | |
| Kwun S et al (2012) * (26) | Melanoma (n=1) | 48/F  PHx, FHx of EndoD & AutoD | | Ipi dose NR | | Headache/4 wks  Drug D/C?: No | | ↓ACTH, cortisol, TSH, FT4, PRL, IGF-1 | | MRI pituitary:  Enlarged | NR | | HD steroids & LT4 | | HCT & LT4 | |
| Lammert A et al (2013) (27) | Melanoma  (n=5) C1,3-5,7  Prostate Ca  (n=2) C2& 6 | C1: 55/F  C2: 73/M  C3: 31/M  C4: 73/M  C5: 74/M  C6: 63/M  C7: 77/M  PHx, FHx of EndoD & AutoD NR | | Ipi  C1-6:3 mg/kg q3wks  C7: 10 mg/kg q3 wks | | C1: fever, headache/9 wks;  C2: nausea, malaise/6 wks;  C3: exhaustion/12 wks  C4: Sx NR/12 wks  C5: Headaches, dizzy/5 wks  C6: Suspected to have hypophysitis/12 wks  C7: Hypoglycemia /32 wks  Drug D/C?: NR C1-4,6.  C5 & 7:Yes | | C1: ↓TSH,LH, FSH,PRL  C2: ↓ACTH, PRL  C3:↓ACTH, TSH, LH, FSH, T  C4: ↓TSH, FT4, ACTH, cortisol, LH, FSH & T. Abn ACTH Stim.  C5: :↓ACTH, TSH, LH, FSH, T  C6: Low NL TSH, FT4, FT3. Other pituitary hormones normal.  C7: ↓ACTH, cortisol, LH, GH | | C1,2: NR  C3: Enlarged  C4: NL  C5: Enlarged  C6: Not done  C7: NR | NR | | C1-3:Iv HD steroids  C3: LT4 & T  C4: Fluocortolone + LT4  C5: HD steroids, LT4 &T.  C6:HD steroids + LT4.  C7:HCT + LT4. | | 3 stable disease  3 progressive disease  1 partial response | |
| Anderson L et al (2013) (28) | Melanoma  (n=1) | 55/F  PHx, FHx of EndoD & AutoD NR | | Ipi dose NR | | Fatigue /16 wks  Drug D/C?: NR | | ↓TSH, FT4  ↓cortisol,Abn ACTH stimulation test. NL PRL, IGF-1 | | Normal | NR | | HCT, LT4 | | Recovered | |
| Leonard D et al (2013) (29) | Melanoma (n=1) | 76/F  PHx, FHx of EndoD & AutoD NR | | Ipi 3mg/kg q3 wks | | Headache, fatigue/10 wks  Drug D/C?: NR | | ↓ACTH, cortisol, TSH, FT4, LH/FSH, [Na+] | | MRI pituitary:  Enlargement | NR | | Steroids & LT4. | | Steroids & LT4 | |
| Sarvaideo JL et al (2013)*  (30) | Melanoma  (n=1) | 63/F  PH of hypo- thyroidism., FHx of EndoD & AutoD NR | | Ipi dose NR x 4 doses | | Headaches, weakness/ 13 wks  Drug D/C ?: NR | | ↓ACTH, cortisol, TSH, FT4, LH/FSH | | MRI pituitary:  Enlargement | NR | | HD Steroids | | NR | |
| Thompson BM (2013)*  (31) | Melanoma (n=1) | 52/M  PHx, FHx of EndoD & AutoD NR | | Ipi dose NR x3 doses | | Headache, fatigue, anorexia/12 wks  Drug D/C?: No | | ↓TSH, ↑FT4. –ve TSI & TPO Ab.  ↓cortisol.-ve 21-hydroxylase Ab. ↓LH, FSH low NL T | | Brain MRI: No metastasis | NR | | Steroids & methimazole | | Steroids  THyroiditis resolved. | |
| Ahmed MK et al (2013)  (32) | Melanoma (n=3) | C1:60/F  C2: 71/M  C3: 74/M  PHx, FHx of EndoD & AutoD NR | | C1: Ipi dose NR x 4doses  C2:Ipi dose NR x4 doses  C3: Ipi dose NR x4 doses | | C1: Symptoms NR/20 wks  C2: Muscle weakness, fatigue/10 wks  C3: Headaches, fatigue /12 wks  Drug D/C? : NR | | C1: central adrenal insufficiency  Other hormones NL  C2::↓ACTH, TSH, LH, FSH  C3: ↓ACTH, TSH, LH, FSH | | MRI pituitary:  C1: NL  C2&3: Hypophysitis | NR | | C1: HCT  C2:HCT, LT4 & T  C3: HCT, LT4, T | | C1: HCT  C2: HCT, LT4, T  C3: HCT, LT4, T | |
| Gil SM et al (2013)* (33) | Prostate cancer (n=1) | 52/M  PHx, FHx of EndoD & AutoD NR | | Chemo. Then, ipi dose NR x 2doses | | Lethargy, weakness / 6 wks  Drug D/C?: NR | | ↓TSH, FT4, LH,FSH, T, PRL, IGF1 | | MRI Pituitary: NL | NR | | HD steroids, LT4, LT3 | | Patient died | |
| Min L et al  (2013)* (34) | Melanoma  (n=1) | 56/F  PHx, FHx of EndoD & AutoD NR | | Ipi dose NR | | Fatigue & headache/12 wks  Drug D/C: NR | | ↓ACTH cortisol,aldosterone  Abn ACTH Stimulation test | | MRI pituitary: enlarged  Abd CT: enlarged adrenal glands | NR | | HCT | | Discharged | |
| Assi H et al (2013) (35) | Melanoma  (n=1) | 54/M  PHx, FHx of EndoD & AutoD NR | | Ipi dose NR | | Headache/9 wks  Dose D/C? : Yes | | Diagnosed to have hypopituitarism by Endocrinology. | | Hypophysitis | NR | | HD Prednisone  LT4 | | Recovered.  Prednisone D/C 5.5 years later. | |
| De Hollanda A et al (2013) (36) | Melanoma (n=3) | C1: 41/M  C2:51/M  C3:65/M  PHx, FHx of EndoD & AutoD NR | | Ipi Dose NR | | C1: Headache & ↓libido/9wks  C2: Asthenia/9 wks.  C3: Asthenia/9 wks  Drug D/C?: NR | | C1: NL ACTH, ↓cortisol,  ↓TSH, FT4, LH, T, IGF1.  C2: ↓cortisol, ACTH, ↓FT4, NL TSH LH, FSH & Estradiol  C3: ↓ACTH, cortisol, TSH, FT4,LH, FSH &T | | C1: Enlarged  C2: Normal  C3: Normal | NR | | iv HD steroids + LT4 & T in C1 & C3. | | Permanent replacement therapy. | |
| Hermes I et al (2013)* (37) | Melanoma  (1) | 37/M  PHx, FHx of EndoD & AutoD NR | | Ipi double-blind clinical trial 3mg/kg vs 10 mg/kg | | Fatigue, ↓libido/6 wks  Drug D/C: NR | | ↓Thyroid & T | | NR | NR | | HD steroids, LT4 & T | | Steroids, LT4 & T | |
| Burgess D * et al (2013) (38) | Melanoma  (n=1) | 72/M  PHx, FHx of EndoD & AutoD NR | | Ipi dose NR | | Headache, fatigue + diplopia/ 9 wks.  Drug D/C?:NR | | ↓ACTH, cortisol, LH, PRL, Na+  Subclinical hypothyroid | | Enlarged | NR | | HD steroids | | Recovered; stayed on steroids | |
| Chodakiewitz Y et al (2014) (39) | Melanoma  (n=3) | C1:45/F  C2:65/F  C3:63/F  No Hx AutoD  PHx, FHx of EndoD NR | | Ipi 3mg/kg q3w x4 doses | | C1: Headaches / 8 wks.  Drug D/C: Yes  C2: Blurry + fatigue/11 wks. Drug D/C Yes  C3: Fatigue/12 wks  Drug D/C NR | | C1: ↓FT3  C2: ↓TSH, FT4, TT3  ↓cortisol, [Na+]  C3: ↓TSH, FT4, TT3  ↓cortisol | | C1-3: Enlarged  After Rx, Pituitary normalized | NR | | C1: HD steroids + LT4  C2: HD steroids + LT4  C3: HD steroids + LT4 | | C1-3: Recovered  HCT & LT4 | |
| Rodriguez BT et al (2014) (40) | Melanoma  (n=2) | C1:46/M  C2:65/F  PHx, FHx of EndoD & AutoD NR | | Ipi 3mg/kg q3wks x4 doses | | C1: Headaches, malaise, fatigue/9wks  C2: Fatigue, wt gain / 9wks  Drug D/C?: NR in both. | | C1&2: ↓TSH, FT4  Low NL ACTH, cortisol  ↓LH, FSH | | C1: slightly enlarged  C-2: Enlarged. Returned to NL after Rx. | NR | | C1,2: HD steroids  +LT4 | | Both recovered  HCT + LT4 | |
| Nallapaneni N et al (2014) (41) | Melanoma  (n=1) | 62/M  PHx, FHx of EndoD & AutoD NR | | Ipi 3m/kg q3w x4 doses | | Nausea, rash, vomiting, /3 wks. DI:- Polyuria, polydipsia.  Drug D/C?: NR | | ↓TSH, FT4, ACTH, Cortisol, LH, FSH, PRL, IGF-1  Water deprivation = partial DI | | NL | NR | | HD HCT, LT4  Desmopressin | | Discharged on these 3 meds | |
| Faje A et al (2014) (42) | Melanoma  (n=17) | C1:73/M;C2:59/M  C3:65/M,C4:60/M  C5:73/M;C6:75/M  C7:66/F; C8:80/M  C9:82/M;C10:40/M;C11:71/M;C12:78/M; C13:75/F; C14:70/M; C15:70/M; C16: 59/M; C17:65/M  PHx, FHx of EndoD & AutoD NR | | C1,2,5,7,9,11-17: Ipi 3mg/kg q3w  C3,4,5,8,9: Ipi 10mg/kg Q2-4 wks | | Headache, fatigues, anorexia,  Onset:C1,2,6,11-14,17::9wks; C3,4,1016: 6 wks;  C5, 7-9,15: 12 w  Drug D/C?: NR | | 3 pts ↓Na+  17 pts ↓TSH, FT4  7 pts ↓cortisol, abn ACTH stim, 6 pts↓ ACTH.  14 pts ↓T, 11 pts ↓LH +FSH | | All enlarged.  15 pts had pituitary glands that normalized | NR | | 15 pts iv HD steroids  1 supraphysiological dose steroids  17 pts on LT4  14 pts on T | | Discharged. 2 pts recovered gonadal,1 adrenal and 1 thyroidal function. | |
| Buddhdev K et al (2014)* (43) | Melanoma  (n=1) | 69/M  PHx, FHx of EndoD & AutoD NR | | Ipi 10mg/kg q3 wkx | | Headache, nausea, vomiting/ 9wks  Drug D/C?: Yes | | ↓ACTH, cortisol  ↓TSH, NL FT4  ↓Na+ | | Enlarged | NR | | HD steroids  LT4 | | Required long-term steroids | |
| Marlier J et al (2014) (44) | Melanoma  (n=4) | C1:56/F  C2:31/M  C3:81/M  C4:78/M  PHx, FHx of EndoD & AutoD NR | | C1,2:Ipi 10mg/kg q3w  C3,4:Ipi 3mg/kg q3w | | C1: Headache,↓libido/6 wks. Drug D/C: Yes  C2: Fatigue, ↓BP /12 wks. Drug D/C: Yes  C3:Lethargy/9 wks  Drug D/C: Yes  C4: Headache /6 wks.  Drug D/C: Yes | | C1: ↓ACTH, cortisol, DHEAS, ↓TSH, GH,↓LH,FSH  C2: ↓ACTH, cortisol, low DHEAS  ↓TSH, GH, ↓FSH,LH  C3: ↓cortisol, ↓TSH,  ↓LH , FSH  C4: ↓cortisol, ↓TSH,  ↓LH ,FSH | | C1: Enlarged  C2: Enlarged  C3: NL  C4: NL | NR | | C1: HCT, LT4, DHEA  C2: HCT  C3: HCT & LT4  C4: HCT + LT4. | | C1: Recovered, on HCT  C2: Recovered on HCT  C3: Recovered on LT4  C4: Recovered on steroid & LT4. | |
| Ryder M et al (2014) (45) | Melanoma  (n=19) | C1:54/M  C2:71/M  C3:69/M  C4:69/M  C5:61/M  C6:75/F  C7:38/F  C8:78/M  C9:71/M;  C10:58/F  C11:74/M  C12: 69/F  C13: 71/F  C14:77/F  C15:78 /M  C16: 47/F  C17: 73/F  C18:52/F  C19:74/M  PHx, FHx of EndoD & AutoD NR | | Ipi C2,4,5,6-8,16-19: 3mg/kg  C1,3,9- 15:  10mg/kg | | C1:Fatigue/13 wks  C2: Fatigue, sleepy/20 wks  C3: Nausea, emesis/24 wks  C4: Headache,↓BP/14 wks  C5: Headaches/12 wks  C6: Fever, sweats/ 10 wks  C7: No Sx/ 60 wks  C8: Nausea, anorexia/13 wks  C9: Fatigue, anorexia/8 wks  C10: Headache/9 wks  C11: Fatigue/18 wks  C12: Fatigue,anorexia/15 wks  C13: Fatigue/35 wks  C14: Headaches/11 wks  C15: Headaches/15 wks  C16: Fever/ 17 wks  C17: Headache/ 17 wks  C18: Fatigue/23 wks  C19: Fatigue/ 76 wks  Drug D/C: NR | | C1:↓ACTH, cortisol, TSH, FT4, FT3, LH+FSH and T  C2:↓ACTH,Cortisol,TSH,FT4  C3:↓cortisol, ACTH  C4: :↓ACTH, cortisol, LH+FSH  C5:↓TSH, ↑FT4, ↓ACTH, cortisol, T  C6: ↓PRL, TSH & NL FT4  C7: ↓TSH, FT4, FT3  C8: ↓cortisol, ACTH, FT4,T  C9: ↓ACTH, TSH  C10: ↓cortisol, TSH, PRL  C11: ↓ACTH, cortisol  C12: ↓ACTH, cortisol  C13: ↓cortisol, low ACTH  C14: ↓cortisol, TSH  C15: ↓ACTH, cortisol, TSH, &T  C16: ↓ACTH, cortisol  C17: ↓ACTH, cortisol  C17: ↓ACTH, cortisol  C18: ↓ACTH, cortisol  C19: ↓ACTH, cortisol | | C1: ↑sella  C2: Not done  C3: empty sella  C4: enhanced  C5: Enlarged  C6: Enlarged  C7: Not done  C8: Not done  C9: Prominence  C10: Enlarged  C11: NL  C12: Not done  C13: NL  C14: Not done  C15: Enhanced  C16: Not done  C17: Enlarged  C18: Not done  C19: NL | NR | | All received HD steroids (except C7)  LT4, T replacement NR | | C1: Recovered  C2: No recovery  C3: No recovery  C4: No recovery  C5: No -adrenal, partial – gonads  C6: Yes  C7: Not assessed  C8: Partial- gonads  C9: Not assessed  C10: Yes  C11: No recovery  C12: No recovery  C13: No recovery  C14: No recovery  C15: No recovery  C16: No recovery  C17: Not assessed  C18: Not assessed  C19: Not assessed | |
| Alkhaddo JB et al (2014)* (46) | Melanoma (n=1) | 53/F  PHx, FHx of EndoD & AutoD NR | | Interleukin & radiation. Then  Ipi 3mg/kg q3w x 4 doses. | | Headaches, cold intolerance & dry skin/ 19wks  Drug D/C: NR | | ↓cortisol ↓Na+  ↓FT4, NL TSH  ↓LH + FSH | | NL | NR | | HD steroids, then prednisone & LT4. | | Pit-Thyroid axis recovered, but not pit-adrenal function. | |
| Iwana S et al (2014) (47) | Melanoma (n=6) C1-6  Prostate Ca (n=1) C7 | C1: 53/M  C2:68/M  C3:59/M  C4:34/F  C5:58/F  C6:72/M  C7:65/M  PHx, FHx of EndoD & AutoD NR | | Ipi  C2,3: 3mg/kg  C1, 4-7: 10mg/kg | | C1: Fatigue/9 wks  C2: Headache/11wks  C3: Headache/6 wks  C4: Fatigue/8 wks  C5: Headache/6 wks  C6: Fatigue/36 wks  C7: Fatigue/12 wks  Drug D/C: NR | | C1: ↓TSH, LH,FSH,ACTH  C2: ↓TSH, LH,FSH,ACTH  C3: ↓TSH, LH,FSH,ACTH  C4: ↓TSH, FT4  C5: ↓TSH, FT4, IGF-1  C6: ↓TSH,FT4, ACTH, cortisol  C7: ↓TSH, FT4, IGF-1 | | C1-3 :Hypophysitis  C4: NL  C5-7: Hypophysitis | NR | | NR | | NR | |
| Tiu C et al (2015) (48) | Melanoma (n=2) | C1: 58/M  C2: 48/M  PHx, FHx of EndoD & AutoD NR | | Surgery, Chemo, then Ipi 3mg/Kg q3wk x4 doses | | C!: Clinical Sx NR / 18 wks.  C2: ↓libido, headaches, /12 wks.  Drug D/C: NR in both cases. | | Both cases: ↓TSH, FT4.  ↓ACTH, cortisol  ↓FSH, LH, T | | Hypophysitis in both | NR | | HD steroids , LT4 & T | | Replacement hormone meds. | |
| Kotwal A et al (2015)* (49) | Melanoma (n=1) | 68/M  PHx ThyD on LT4. FHx of Endo D and AutoD NR | | Ipi 3mg/kg | | Headache, anorexia, diplopia/9 wks  Drug D/C? :No | | ↓T, ↓TSH. NL FT4.(on LT4).  ↓ACTH, cortisol. Annormal ACTH stimulation test | | MRI pituitary:  enhancement |  | | HD steroids & T | | HCT & T | |
| Denman D et al (2015)* (50) | Melanoma (n=2) | C1: 52/M  C2: 56/F  PHx, FHx of EndoD & AutoD NR | | C1: Ipi 10mg/kg q3wks x4doses  C2: Ipi 3mg/kg q3wks x3doses | | C1: Headaches, fatigue/12 wks.  C2: Headache, fatigue/9wks  Drug D/C: Yes in both cases. | | C1: ↓TSH, FT4, cortisol, T  C2: ↓TSH, FT4, cortisol | | Enlarged in both cases. A month later NL. | NR | | iv high dose steroids, then HCT, LT4+ T(C1 only) | | Discharged on replacement hormone meds. | |
| Heaney AP et al  (2015) (51) | Cancer NR  (n=4) | C1: 69/M  C2: 49/F  C3: 55/M  C4: 37/M  PHx, FHx of EndoD & AutoD NR | | Ipi (n=2)  Tremeli (n=2) | | Fatigue, syncope /mean 15 wks of IPILI or TREMELI.  Drug D/C: NR | | In all ↓ACTH, cortisol, TSH  IN C2:↓ FSH +LH | | Abnormality in all | NR | | Glucocorticoids n=4  LT4 n=3  Androgens n=2 | | NR | |
| Majchel D et al (2015) (52) | Melanoma (n=1) | 31/F  PHx, FHx of EndoD & AutoD NR | | Surgery, then Ipi 10mg/kg q3wk | | Headaches/9wks.  Drug D/C: NR | | NL ACTH & low cortisol, ↓TSH & NL FT4  ↓ LH NL FSH & PRL | | Enlarged | NR | | iv HD steroids | | NR | |
| Lam T et al (2015) (53) | Melanoma  (n=10) | C1:60/F  C2:53/M  C3:69/M  C4:61/M  C5:50/M  C6:70/M  C7:46/M  C8:53/M  C9:65/M  C10:63/M  PHx, FHx of EndoD & AutoD NR | | 9 pts (C1-4,6-10) Ipi 3mg/kg q3w x3doses  1 pt (C5) Ipi 10mg/kg q3w x4 | | Headaches, fatigue, dyspnea, etc/ C1:11wks; C2: 12 wks; C3:10 wks; C4:6 wks; C5:24 wks; C6: 7 wks; C7:13 wks; C8: 12 wks; C9:7 wks; C10:7 wks.  Drug D/C?: Yes in 2 & held in 5; No in 3; | | C1: ↓cortisol, TSH  C2: ↓cortisol  C3: ↓ACTH, cortisol, FT4,T, LH,  C4: ↓cortisol  C5: ↓cortisol  C6: ↓cortisol, TSH, FT4,T  C7: ↓ACTH,cortisol,FT4,T, IGF-1  C8: ↓ACTH, cortisol, GH  C9: ↓ACTH, cortisol, TSH, FT4, T  C10: Inappropriately low NL ACTH for cortisol, ↓TSH, T | | 4 pts (C2,6,9,10) enlarged  6 pts (C1,3,4,5, 7,8) NL | NR | | C2,3,5,6: physiological dose steroids.  C1,4,7 & 8: iv HD steroids  C9,10: Intermediate high dose steroids  All: LT4 | | All recovered on hormonal replacement. Ipili restarted in some. | |
| DeSousa SMC et al (2015)† (54) | Melanoma (n=2) | 2 pts in 60s/M  PHx, FHx of EndoD & AutoD NR | | Ipi dose NR | | C1: Headache, fatigue, dizziness/9 wks  C2: Headaches, emesis/9 wks.  Drug D/C: C1: NR: C2: No | | C1: NL ACTH, ↓cortisol, TSH, FT4, T. Inappropriately NL FSH,LH NL, PRL &IGF1  C2: ↓TSH, FT4 , FT3; &T, In appropriately NL LH< FSH;  NL ACTH & cortisol | | C1: Enlarged  normalized after Rx  C2: Normal | NR | | Both HD steroids initially and later  LT4 & T added. | | Both recovered but still on steroid & LT4 | |
| Hanseree P et al (2015)* (55) | Melanoma  (n=1) | 53/F  PHx, FHx of EndoD & AutoD NR | | ipi dose NR | | Headache, fatigue, vomiting/14 wks  Drug D/C: NR. | | ↓TSH, FT4, FT3  ↓cortisol, inappropriately NL ACTH | | Enlarged | NR | | iv HD steroids  LT4 | | Discharged on steroids & LT4 | |
| Albarel F et al (2015) (56) | Melanoma  (n=15) | C1:46/M  C2:61/M  C3: 80/M  C4: 44/M  C5:48/F  C6:54/M  C7:62/M  C8:47/F  C9: 52/M  C10:40/M  C11: 68/M  C12: 51/F  C13: 59/F  C14: 67/M  C15:61/F  PHx, FHx of EndoD & AutoD NR | | C1,2,13,15: Ipi 3mg/kg  C3-12,14: Ipi 10mg/kg | | C1: Headaches (H)+Asthenia (A)/9 wks  C2: H+A/7 wks  C3: H+A/9 wks  C4:H+A/3 wks  C5: H/9 wks  C6:H+A+↓libido (L)/6 wks  C7: H+A+↓L/28 wks  C8: H/14 wks  C9: H+A+↓L/67wks  C10: H/9 wks  C11: H+A+↓L/9 wks  C12: A/10 wks  C13: H/7 wks  C14: A/6 wks  C15: H+A/10 wks  Drug D/C?: NR | | C1: ↓ACTH, TSH, LH, FSH  C2: ↓LH, FSH  C3: ↓ACTH, TSH, LH, FSH  C4: No hormone deficiency  C5: ↓ACTH, TSH  C6: ↓ACTH, TSH, LH, FSH  C7: ↓ACTH, TSH, LH, FSH  C8: ↓ACTH, TSH, LH, FSH  C9: ↓TSH, LH, FSH  C10: ↓ACTH, TSH, LH, FSH  C11: ↓TSH, LH, FSH  C12: ↓ACTH, TSH  C13: ↓ACTH, TSH, LH, FSH  C14: ↓ACTH, TSH, LH, FSH  C15: ↓ACTH,TSH, LH, FSH | | C1.5: Enhanced pituitary (EP)  C2,4,6-8: Enhanced  Contrast (EC)  C9,11,13: EP+EC:  C10,12: EP+EC +Enhanced pituitary stalk (EPS)  C15: EC+EPS  C3: ND | NR | | !! cases: C1-7,9-11,14: HD steroids  4 cases: C8,12,13,15: steroids  5 cases: C3,5,7,9,13: LT4: | | HCT (n=13)  L-T4 (n=2)  T: (n=2) | |
| Araujo PB et al (2015) (57) | Melanoma (n=1) | 60/M  PHx, FHx of EndoD & AutoD NR | | Ipi dose NR | | Headache, fatigue &dizziness/13 wks  Drug D/C: NR | | ↓cortisol, low ACTH;  ↓LH, FSH, T, PRL, IGF-1  Low TSH & FT4 | | Enlarged | NR | | HD steroid  LT4 | | Continued on replacement | |
| Carl D et al (2015) (58) | Prostate Ca (n=1) | 64/M  PHx, FHx of EndoD & AutoD NR | | Ipi 10mg/ kg q3w, then at 3 m intervals | | Encephalopathy, hypophysitis symptoms/12 wks  Drug D/C: No | | ↓TSH, T4 and abnormal TRH test; +ve TPO & TG Abs.  ↓cortisol, T | | NR | NR | | HD steroids  LT4 | | Recovered | |
| Mahzari M et al (2015) (59) | Melanoma  (n=6) | C1: 80/M  C2: 66/F  C3: 54/M  C4: 54/M  C5:56/F  C6: 64/M  PHx, FHx of EndoD & AutoD NR | | Ipi  C1-3,5,6: 3mg/kg q3wk  C4: 10mg/kg q3wk | | C1-5 had Headache & C6 was asymptomatic/  C1: 12 wks; C2:9 wks; C3:11 wks; C4: 15 wks; C5: 9 wks C6: 8 wks.  Drug D/C: NR in all | | C1: ↓ACTH, TSH, LH,FSH, NL PRL  C2: ↓ACTH, LH, NL PRL +FSH  C3: ↓ACTH,T SH, LH,FSH, low  PRL  C4: ↓ACTH, LH,FSH, low PRL  C5: ↓ACTH, T SH, LH, NL PRL+FSH  C6: ↓ACTH,T SH, LH, FSH,low  PRL | | C1,3: Enlarged & enhanced.  C2,4: Enlarged, enhanced and thick stalk  C4,6: NL | NR | | HD prednisone | | Recovery  Adrenal axis 0  thyroid axis 2  Gonadal axis 4 | |
| Yun S et al  (2015) (60) | Melanoma (n=1) | 59/M  No Hx of AutoD.  PHx, FHx of Thy D NR. | | Ipi 3 mg/kg q3wk x4 doses | | Chest pain, dyspnea/24  wks.  Drug D/C: NR | | ↑TSH ↓FT4 (slight)  ↓cortisol, ↓ACTH | | NR | NR | | HD steroids  No LT4 | | Recovered. No LT4.  Subclinical hypothyroidism | |
| Wilson M et al (2016) (61) | Melanoma (n=1) | 53/M  PHx, FHx of EndoD & AutoD NR | | Ipi3mg/kg q3w x3 doses | | Headache/ 9 wks .  Drug D/C: Yes | | ↓Cortisol, TSH,T Inappropriately Nl LH, FSH | | Enlarged | NR | | HD Steroids, LT4,T | | Replacement  Hormone meds | |
| Ohnuma T et al (2016)† (62) | Melanoma  (n=1) | 57/F  PHx, FHx of EndoD & AutoD NR | | Surgery, then Nivo 2mg/kg q3wk x 6 doses., then Ipi 3mg/ kg q3w x2 doses. | | Fever, fatigue, weakness, etc/ 6 wks  Drug D/C: Yes. | | Hypoadrenocorticism | | Hypophysitis  1 m later NL | NR | | iv HD HCT. Then- oral HCT | | Discharged on replacement HCT | |
| Grenier M et al (2016) (63) | Non-small cell lung Carcinoma  (n=1) | 51/M  PHx, FHx of EndoD & AutoD NR | | Nivo dose NR | | Nausea, disorientation, hypotension,  Onset NR  Drug D/C?: Yes | | ACTH & cortisol NR.  Abn ACTH stimulation test  Normal TSH, LH, FSH. ↓Na+ | | NR | NR | | Steroids | | Discharged. MedicationsS NR | |
| Caturegli P et al (2016) (64) | Meso-thelioma  {n=1) | 79/F  PHx, FHx of EndoD & AutoD NR | | Tremeli 10 mg/kg q4w  X3 doses | | Vomiting, diarrhea & fatigue/9 wk.  Drug D/C?: NR | | ↓cortisol, inappropriately NL low ACTH  ↓TSH + FT3  ↓Na” | | Enlarged | NR | | Iv HD steroids  and LT4. | | Patient died | |
| Marques P et al (2016) (65) | Melanoma (n=1) | 67/F  No PHx of EndoD and AutoD. | | Surgery, then Ipi 3mg/kg q3 x4 doses | | Headache, fatigue, nausea/ 9 wks.  Drug D/C?:No. | | ↓cortisol, abn ACTH stim. ↓TSH, NL FT4 (on thyroxine), ↓LH, FSH, & NL IGF-1, PRL | | Hypophysitis | NR | | Oral HCT &LT4 | | Discharged on HCT + LT4 | |
| Koessler T et al (2016)† (66) | Melanoma  (n=1) | 46/F  PHx, FHx of EndoD & AutoD NR | | Surgery, Proton bean, then Ipi 3mg/kg q3w x3 doses | | Symptoms of hypophysitis pan-hypopituitarism / 6 wks.  Drug D/C?: No | | No actual lab values cited but diagnosed to have pan-hypopituitarism | | Hypophysitis | NR | | Iv HD steroids followed by HCT & LT4 | | Continued on replacement HCT + LT4 | |
| Okano Y et al (2016) (67) | Melanoma (n=1) | 50/M  No PHx, FHx of EndoD & AutoD | | Nivo 2mg/kg q3wk | | Anorexia & fatigue/  19wks.  Drug D/C?: NR | | ↓ACTH, cortisol, DHEAS, GH, IGF-1,  Low TSH, FT4, FT3, T  Abn CRH, TRH, GnRH & GHRH tests | | Mild enlargement | NR | | iv HD steroids | | Recovered but remained on steroids | |
| Miller AH et al (2016) † (68) | Melanoma (n=3) C1-3  Pap thy Ca  (n=1) C4  Renal Cell Ca (n=1) C5 | C1:65/M  C2:52/M  C3:60/F  C4:60/F  C5: 72/M  PHx, FHx of EndoD & AutoD NR | | C1-3 Ipi dose NR x4 doses  C4: Ipi dose NR x3 doses  C5: Ipi dose NR + Nivo dose NR, x4 doses | | C1: headache,fatigue/13 wks  C2:Headache,fatigue/14 wks  C3:Confusion/ 19 wks  C4: Headache, fatigue/14wks  C5:confusion,fatigue/15 wks  Drug D/C?: NR | | C1; ↓ACTH,TSH,LH,FSH &GH  C2: ↓ACTH,TSH,LH,FSH  C3: ↓ACTH,TSH,  C4: ↓ACTH,LH,FSH  C5: ↓ACTH,TSH,LH,FSH | | Enlargement in C1-4.  C5: NL | NR | | Iv HD steroids | | Recovered on steroid replacement | |
| Todd A et al (2016)* (69) | Melanoma  (n=7) | Mean 62.1/ 4 M+3F  PHx, FHx of EndoD & AutoD NR | | Ipi dose NR | | Lethargy, headache. adrenal crisis/ 9-12 wks  Drug D/C: NR | | All had ↓ACTH, Cortisol, ↓TSH, T4  5/7 ↓LH & FSH  2/7 ↓PRL | | 1/7 enlargement | NR | | 1^st^ 4 pts high dose steroid, LT4 & T  Next 3 physiological dose steroids. | | 1^st^4 pts replacement meds  Next 3 no replacement | |
| Freeeman-Kellar M et al (2016) (70) | Melanoma  (n=1) | Age/Gender NR  PHx, FHx of EndoD & AutoD NR | | Novi dose NR | | Fatigue & headaches/20 wks.  Drug D/C?: NR | | ↓ACTH, cortisol | | NR | 2 | | HCT | | HCT | |
| Vancieri G et al (2016) (71) | Melanoma (n=1) | 72/M  PHx, FHx of EndoD & AutoD NR | | Ipi dose NR x4 doses. | | Fatigue & presyncopal episodes/ 38 wks.  Drug D/C?: NR | | ↓TSH,FT4,ACTH,cortisol, LH, T, PRL, IGF1. | | Pituitary trophy | NR | | HCT, LT4 & T | | HCT, LT4 & T | |
| Chon D et al (2016)* (72) | Lung Carcinoma (n=1) | 60/M  PHx, FHx of EndoD & AutoD NR | | Ipi dose NR x4 doses | | Fatigue, anorexia, weight loss/12 wks  Drug D/C?: NR | | ↓ACTH, cortisol. Abnormal synacten test. | | MRI pituitary Enlarged | NR | | HD steroids | | HCT | |
| Gill PM et al (2016)* (73) | Melanoma (n=1) | 59/M  PHx of hypothyroidism., FHx of EndoD & AutoD NR | | Chemo. Then, Ipi dose NR. | | Headache, fatigue/10 wks  Drug D/C?: NR | | Central hypothyroidism hypogonadism and adrenal insuffiiciency | | MRI pituitary:  Enlarged | NR | | HD steroids  LT4 & T | | NR | |
| Telford R et al (2016)* (74) | Melanoma (n=1) | 73/F  PHx, FHx of EndoD & AutoD NR | | Ipi dose NR | | Headache, clumsiness of leg /9 wks  Drug D/C? : NR | | ↓TSH, FT4, [Na+] | | MRI pituitary:  Enhancement & enlargement | NR | | HD steroids & LT4 | | HCT, LT4 | |
| Humayun MA (2016)† (75) | Melanoma (n=1) | 55/M  No PHx & FHx of DM and AutoD. | | 1^st^ RX: dacarbazine. 2^nd^ Rx: Ipil Dose NR 4 doses over 2 wks  3^rd^ line Rx: Pembro Dose NR x 10 cycles | | After Ipi: Fatigue & blurry vision  After Pembro: Fatigue, polydipsia, polyuria/27 wks (9 cycles)  Drug D/C? Yes | | After Ipi: Had ↓TSH, FT4, Cortisol, LH, FSH, T  After 9 cycles of Pembro  ↑A1c, ↑Glu, ↓pH, ↑ β-OHB , ↓ C-peptide  -ve antiGAD 65 Ab | | MRI of pelvis: Edema & degenerative changes | NR | | After Ipi: HD steroids for hypopituitarism.  After Pembro: Fluids, insulin for DKA. | | Insulin, HCT | |
| Lowe JR et al (2016) (76) | Melanoma  (n=1) | 54/M  PHx, FHx of ThyD & AutoD NR. | | Nivo 1mg/kg q3wks+Ipi 3mg/kg/ (Doses of  Check-Mate 069 protocol. | | Tachycardia + hot flashes./2weeks after 1^st^ dose  No hypothyroid Sx/ 6 wks later 2^nd^ dose.  Weakness, nausea, vomiting/ 2wks after 3^rd^ dose, (DKA Sx/16 weeks)  Drug D/C ?: Yes (also had hepatitis & colitis) | | 2 wk s post 1^st^ dose, ↓TSH, ↑FT4,FT3., +ve TRAb.  +ve anti-microsomal Ab  Then ↑TSH, ↓FT4 +FT3  2 wks post 3^rd^ dose,  Glu, A1c NR. β-OHB↑, ↓ C-pep., +ve GAD65 Ab.  ↓ACTH, cortisol, T  ACTH stimulation test abn | | MRI pituitary: NL | NR | | HD steroids + β blocker. When ↑TSH, Lt4 started  Insulin for DKA.  HD steroids for hypopituitarism | | Insulin-dependent  LT4 NR | |
| Bot I et al (2017) (77) | Melanoma (n=1) | 66/M  PHx, FHx of EndoD & AutoD NR | | Ipi dose NR | | Headache/9 wks.  Drug D/C?: Yes | | ↓Na+,  ↓ TSH, FT4,  ↓LH & T | | Enlarged | NR | | HD prednisone, LT4 and T PO | | A year later needed prednisone + T | |
| Ishikawa M et al (2017) † (78) | Melanoma  (n=1) | 55/M  PHx, FHx of EndoD & AutoD NR | | Chemo, then Nivo 2mg/Kg q3wk | | Infusion reaction /12 wks. Malaise, myalgia,/13 wks  Drug D/C: NR | | ↓ACTH, cortisol  NL Thyroid hormones  After 8^th^ dose, became hypothyroid. | | NL | NR | | HD steroids | | Discharged.  Medications NR | |
| Mansoor S et al (2017)* (79) | Melanoma (n=1) | 46/F  PHx, FHx of EndoD & AutoD NR | | Pembro D/C, then Ipi+Nivo dose NR | | Headache, hypotension/ onset NR.  Drug D/C?: NR | | ↓ACTH, cortisol, Na+  ↓TSH, FT4, FT3,  ↓PRL, Na+ | | Enlarged | NR | | Iv HD steroid  LT4 | | Recovered. Remained on steroids and LT4 | |
| Konda B et al (2017) (80) | Melanoma  (n=1) | 70/M  PHx, FHx of EndoD & AutoD NR | | Ipi 3mg/kg q3wks x4 doses | | Headaches, fatigue, weakness /13 wks.  Drug D/C?: NR | | ↓cortisol, ACTH  Abn ACTH Stim test  ↓FT4, NL TSH | | mild enlargement | NR | | NR | | NR | |
| Fujimura T et al (2017)† (81) | Melanoma  (n=1) | 68/M  PHx, FHx of EndoD & AutoD NR | | Nivo 2mg/kg q3wks  Followed by irradiation | | Clinical Sx NR/28 wks (4 wks after irradiation)  Drug D/C: NR | | ↓↓ ACTH & cortisol. Abn CRH test. NL TSH, FT4 FT3  NL GHRH, LHRH & TRH stimulation tests | | MRI – Pituitary  NL | NR | | NR | | NR | |
| Oda T et al  (2017)† (82) | Melanoma  (n=1) | 85/M  PHx, FHx of EndoD & AutoD NR | | Nivo 2mg/kg q3 wks | | AV block after 12 wks.  At 20 wks, fatigue, hypotension.  Drug D/C?: NR | | At 20 wks ↓FT4, ↑TSH.  ↓ACTH & cortisol. CRF test abnormal.  Other pituitary hormones NL | | NR | NR | | Oral HCT | | Oral HCT | |
| Neril R et al  (2017) (82) | Non-small cell lung Carcinoma  (n=1) | 84/M  No PHx of EndoD & AutoD.  FHx of EndoD NR. | | Nivo dose NR. | | Malaise, confusion, hypotension etc./after 18 wks  Drug D/C?: NR | | ↓ ACTH, ↓↓cortisol  Abn ACTH stimulation test  NL TSH, FT4, LH, FSH, PRL, IGF1, T | | MRI-Pituitary  NL | NR | | Steroids | | Discharged on replacement steroids | |
| Narahira A et al (2017)† (84) | Melanoma  (n=1) | 76/F  PHx, FHx of EndoD & AutoD NR | | Nivo 2mg/kg q3wks | | Anorexic, bradykinesia/27 wks.  Drug D/C: Yes | | ↓ACTH, ↓↓ cortisol. Abn CRF stimulation test.  NL TSH, FSH, LH, PRL,GH | | NR | NR; | | Iv steroids | | HCT | |
| Kitajima K et al (2017) (85) | Melanoma  (n=2) | C1: 39/M  C2: 50/F  PHx, FHx of EndoD & AutoD NR | C1: Chemotherapy,  Nivo 2mg/kg q3w  Case 2: After 13 doses of Nivo, started ipi dose NR | | C1: General malaise/39 wks  C 2: Fever, severe fatigue, dizziness/45 wks after ipili  Drug D/C?: NR | | ↓ACTH ↓cortisol, DHEAS,  Abn ACTH Stimulation test  Abn CRF stimulation test  NL TSH, FSH, LH, T, PRL, IGF-1.  NL GHRH, LHRH & TRH tests in both. C1:↓T; C2:↓Estradiol | | MRI – Pituitary  NL | | C1: 3  C2: 4 | C1: Oral HCT  C 2: iv HCT then PO  LT4 | | Discharged Medications NR | |  |
| Brilli L et al (2017) (86) | Melanoma  Prostate Ca  (n=9) | C1:48/M  C2:72/F  C3:76/F  C4:61/F  C5: 52/M  C6: 66/M  C7: 67/M  C8: 62/F  C9:73/F  PHx, FHx of EndoD & AutoD NR | | Ipi C1,3,5-9:  3 mg/kg q3w x 4 doses  C2,4:  10 mg/kg q3wks x 4 doses | | C1:Fatigue,↓weight/14 wks  C2:Headache, nausea/7 wks  C3: Headache/6 wks  C4: Headache/5 wks  C5: Fatigue/19 wks  C6: Fatigue,headache/40 wks  C7: Headache, nausea/7 wks  C8: Headache/14 wks  C9: Headache/8 wks  Drug D/C?: NR | | C1:↓TSH,ACTH,FSH/LH,PRL  C2: ↓TSH, ACTH, FSH/LH  C3: ↓TSH, ACTH, FSH/LH,IGF-1  C4: ↓TSH, ACTH, FSH/LH,PRL  C5: ↓ACTH  C6: ↓ACTH  C7: ↓ACTH, FSH/LH,PRL  C8: ↓TSH, ACTH,FSH/LH  C9: ↓TSH,ACTH,FSH/LH,PRL | | C1:Contraction  C2:Enlarged  C3:Enlarged  C4:Enlarged  C5:NL  C6:Enlarged  C7:NL  C8,9: Not done | NR | | All placed on physiological steroid replacement. | | Survived 4-51 m after IPILI.  Recovered  Adrenal function.0  Thyroid  function 6  Gonadal  function 4 | |
| Singh D et al (2017)† (87) | Melanoma  (n=1) | 47/M  PHx, FHx of EndoD & AutoD NR | | Surgery, Irradiation, then Ipi 3mg/kg q3w x4 doses | | Headache, nausea/ 8 wks  Drug D/C: Yes | | ↓T, TSH, FT4, cortisol  Inappropriately Low ACTH, NL PRL, LH/FSH | | Enlarged | Nr | | HD steroids  LT4  T | | Discharged on these medications | |
| Otsubo K et al (2017) (88) | Lung adeno carcinoma (n=2) | C1: 68/F  C2: 66/M  PHx, FHx of EndoD & AutoD NR | | C1: Chemo. Then, Nivo dose NR x2 doses then D/C.  C2: Chemo.Then, Nivo dose NR x11 doses | | C1: Fatigue,anorexia/18 wks  4 months after last dose  C2: Fatigue, diarrhea/ 46 wks  6 months after last dose  Drug D/C?: Yes in both | | C1: 18wks:↓ACTH, cortisol, eosinophils.  C2: 18wks:↓ACTH, cortisol, eosinophils | | MRI pituitary C1:NL.  C2: NL | NR | | C1: HD steroids followed by HCT  C2: HD steroids followed by HCT | | HCT in both. | |
| Anderson C et al (2017) *  (89) | Melanoma  (n=1) | 47/M  PHx, FHx of EndoD & AutoD NR | | Surgery, chemo..Then ipili dose NR x4 doses. Then,pembro dose NR x3 doses | | Symptoms NR/( wks  Headache/10 days after 1^st^ episode  Drug D/C? : NR | | ↓TSH,↑FT4. –ve TPO & TR Ab.  NL Synacten test. TSH normal 5 days later.  .2^nd^ episode: ↓TSH, LH/FSH | | ThyUS & scan NL.  MRI pituitary NL | 2 then 3 | | HD steroids  LT4, testosterone | | Full recovery | |
| Bhalla S et al (2017)* (90) | Melanoma  (n=1) | 70/F  PHx, FHx of EndoD & AutoD NR | | Nivo + Ipi dose NR x6 doses.followed by nivo dose NR. | | Fatigue, nausea, vomiting/14 wks.↓BP, syncope/18 wks  Drug D/C? : NR | | 17wks: ↑TSH, ↓FT4  18 wks:↓ACTH, cortisol,LH,FSH  [Na+] | | MRI pituitasry: Enhancement |  | | 17 wks:LT4  18wks: HD steroids | | NR | |
| Mian N et al (2017)* (91) | Melanoma (n=1) | 72/M  PHx, FHx of EndoD & AutoD NR | | Ipi dose NR x3 doses | | Malaise, anorexia, headache/11wks  Drug D/C?: NR | | ↓ACTH, cortisol, TSH & Glu | | MRI pituitary: Enlarged, enhanced | NR | | Glucose  HD steroids  LT4 | | NR | |
| Okiro J et al (2017)* (92) | Melanoma (n=1) | 70/F  PHx, FHx of EndoD & AutoD NR | | Ipi dose NR x4 doses | | Anorexia, malaise, confusion/14 wks  Drug D/C? : NR | | ↓cortisol, ACTH, LH, FSH,estradiol, TSH  Synactin test abnormal | | MRI pituitary  NL | NR | | HD steroids | | NR | |
| Zeng MF et al (2017) (93) | Renal cell carcinoma  (n=1) | 54/M  Has T2DM  FHx of EndoD & AutoD NR | | Chemotherapy and Radiotherapy. Then,  Nivo 2mg/kg q2wk | | No hypothyroid symptoms/  8 wks  Hypoglycemia/ 24 wks  Drug D/C?: Yes | | 8 wks:↑TSH, ↓FT4, ↓FT3,  ↑TPO, ↑TG Ab  24 wks: ↓cortisol, low ACTH, ↓DHEAS.  NL LH, FSH, T & PRL | | Not done | NR | | LT4, HCT | | LT4, HCT | |
| Takaya K et al (2017) (94) | Lung adeno carcinoma  (n=1) | 75/M  PHx, FHx of EndoD & AutoD NR | | Chemo and radiation. Then, Nivo 3m/kg q2wks | | Fatigue, anorexia/24 wks  Drug D/C? : NR | | ↓ACTH, cortisol, DHEAS  NL TSH, FT4,  CRF abn. LHRH,GNRH &TRH stimulation tests all NL. | | MRI Pituitary NL | NR | | HD steroids | | HCT | |
| Marchand L et al (2017)† (95) | Non-small cell lung cancer: leiomorphic lung carcinoma (n=1) | 55/M  PHx & FHx of DM and AutoD NR. | | Chemo; then nivo dose NR x9 doses | | DKA Sx / 19 wks.  Asthenia, nausea, ↓BP/32 wks  Drug D/C?: Yes | | 19 wks:↑A1c,↑Glu,↓C-pep. NL GAD65, IA-2 & ZnT18 Ab.  32 wks: ↓ACTH, cortisol, [Na+]. ↑PRL. Other pituitary hormones NL. | | MRI NL pituitary @ 36 wks. | NR | | 28 wks: Insulin & fluids  32 wks: HCT | | Insulin & HCT | |
| Kanie K et al (2018) (96) | Non-small cell lung cancer (n=2) | C1: 61/M  C2: 68/M  PHx, FHx of EndoD & AutoD NR in both. | | C1: Chemotherapy, radiosurgery, chemotherapy, then Atezo 1200 mg q3wks x19 doses  C2: Atezo 1200 mg q3 wks x18 doses | | C1: Anorexia, malaise, diarrhea/56 wks  Drug D/C?: NR  C2: Anorexia, malaise/52 wks  Drug D/C?: NR | | C1: ↓ACTH ↓cortisol.  Abn Insulin tolerance test  NL GH, PRL, TSH response tests & basal FSH, LH, T.  C2: Low NL am ACTH, cortisol. ↓DHEAS, Blunted insulin tolerance test. NL GH, PRL, TSH response tests | | MRI pituitary:  C1: atrophy.  C3: NL | NR | | C1: HCT  C2: HCT | | C1: HCT  C2: HCT | |

C: Case; *: Abstract; †: Letter to the editor; M: Male; F: Female; NR: Not reported; ND: Not done; NL: Normal; BP: Blood pressure;

PHx: Personal history; FHx: Family history; EndoD: Endocrine disease; AutoD: Autoimmune disease;

ipi: Ipilimumab, Nivo: Nivolumab; Pembro: Pembrolizumab; Tremeli: Tremelimumab; Atezo: Atezolizumab; CTLA-4: Cytotoxic T-Lymphocyte Antigen-4;

Sx: Symptoms; D/C: Discontinued; q:every; Wks: weeks; NR: Not reported; ↓: Decreased; ↑: Increased; abn: abnormal;

ACTH: Adrenocorticotropic hormone;CRH: Corticotropin- releasing hormone; DHEAS: Dehydroepiandrosterone sulfate; TSH: Thyroid stimulating hormone; FT4: Free thyroxine; FT3: Free triiodothyronine; LH: Luteinizing hormone; FSH: Follicle stimulating hormone;T: Testosterone; PRL: Prolactin; GH: Growth hormone; IGF1: Insulin growth factor-1; Na: Sodium; DI: Diabetes insipidus;

CTCAE: Common terminology criteria for adverse events; MRI: magnetic resonance imaging; CT: Computerized tomography;

Rx: Treatment; iv: Intravenous; HCT: Hydrocortisone; LT4: Levothyroxine; Chemo: Chemotherapy;
