# Supplementary material for: Spectrum of immune checkpoint inhibitors-induced endocrinopathies in cancer patients: a scoping review of case reports
Source: Clin Diabetes Endocrinol. 2019 Jan 22;5:1. doi: 10.1186/s40842-018-0073-4 (PMC6343255; doi:10.1186/s40842-018-0073-4)
Supplement: Supplementary file 4 — Appendix 4. Cases of Immune Checkpoint Inhibitors-Induced Endocrinopathies – Thyroid Disorders: (a) Thyrotoxicosis (b) Hypothyroidism. (DOCX 48 kb) [file 40842_2018_73_MOESM4_ESM.docx]

**Appendix 4: Cases of Immune Checkpoint Inhibitors-Induced Endocrinopathies –**

**Thyroid Disorders: (a) Thyrotoxicosis**

| **Authors**  **(Year)**  **(Reference)** | **Cancer/# of patients** | **Age/Gender**  **Pertinent History** | **Drug /dose** | **Clinical Symptoms/ onset after 1^st^ dose**  **Drug D/C?:** | **Laboratory investigations** | **Imaging** | **CTCAE**  **Grade** | **Treatment** | **Outcome** |
| --- | --- | --- | --- | --- | --- | --- | --- | --- | --- |
| **Graves’ disease** |  |  |  |  |  |  |  |  |  |
| Min L et al (2011) (97) | Melanoma  (n=1) | 51/F  No PHx of ThyD. FHx of ThyD, AutoD NR | Ipi 10mg/kg x4 doses | Proptosis, eye pain, conjunctivitis/ 13 wks  Drug D/C?: NR | NL TSH & FT4. ↑TPO & TG Ab. +ve TSI ( 17 m later). | MRI: enlarged extraocular muscles | NR | Iv HD steroids | Improved on steroids. When tapered Sx relapsed. Eventually better |
| Borodic G et al (2011) (98)  Borodic G et al(2014) † (99) | Melanoma  (n=1) | 51/F  PHx, FHx of ThyD & AutoD NR | Surgery then Ipi/dose NR.  Continued on Ipi | 2011: Proptosis, diplopia, keratitis 6 wks  Drug D/C: NR  2014: weight loss, anxiety | 2011: NL TSH, FT4, sl↓FT3. +ve TRAb  2014: FT3 ↑↑ ↑ FT4↑. TPO + TG Ab +ve. | MRI; enlarged extraocular muscles | NR | 2011: HD Steroids & cantholysis  2014: Antithyroid Rx NR | Improved |
| Krull I et al (2014)* (100) | Melanoma  (n=1) | 66/M  No PHx of ThyD. FHx of ThyD, AutoD NR | Ipi / dose NR. | Hyperthyroidism Sx/ 6 wks  Drug D/C?: NR | ↑FT4, +veTRAb, | ↑thyroid perfusion | NR | Carbimazole  10 mg TID, tapered to 5 mg OD, then D/C. | Progressed to hypothyroidism. Needed LT4 8 weeks after presentation to stay euthyroid |
| Azmat U et al (2016) (101) | Melanoma  (n=1) | 67/M  No PHx of ThyD. FHx of ThyD, AutoD NR. | Ipi 3mg/kg q3w x4doses | Hyperthyroidism Sx/ 6 wks  Drug D/C?: Yes | ↓TSH, FT4,↑↑FT3  +ve TSI | Diffuse ↑RAI uptake | NR | Methimazole 15 mg BID.  IPILI 2 more doses. | During nodes resection, papillary microcarcinoma after left thyroidectomy.  Started on LT4. |
| Lowe JR et al (2016) (76) | Melanoma  (n=1) | 54/M  PHx, FHx of ThyD & AutoD NR. | Nivo 1mg/kg q3wks+Ipi 3mg/kg/ (Doses of  Check-Mate 069 protocol. | Tachycardia + hot flashes./2weeks after 1^st^ dose  No hypothyroid Sx/ 6 wks later 2^nd^ dose.  Weakness, nausea, vomiting/ 2wks after 3^rd^ dose, (DKA Sx/16 weeks)  Drug D/C ?: Yes (also had hepatitis & colitis) | 2 wk s post 1^st^ dose, ↓TSH, ↑FT4,FT3., +ve TRAb.  +ve anti-microsomal Ab  Then ↑TSH, ↓FT4 +FT3  2 wks post 3^rd^ dose,  Glu, A1c NR. β-OHB↑, ↓ C-pep., +ve GAD65 Ab.  ↓ACTH, cortisol, T  ACTH stimulation test abn | MRI pituitary: NL | NR | HD steroids + β blocker. When ↑TSH, Lt4 started  Insulin for DKA.  HD steroids for hypopituitarism | Insulin-dependent  LT4 NR |
| Gan EH et al (2017) (102) | Melanoma (n=1) | 55/M  No PHx, FHx of ThyD & AutoD. | Surgery, chemo, then Tremeli dose NR q3m x8doses. | Weight loss/After 8 years (CT rollover TREME q6m x8 years)  Drug D/C: Yes. | ↓TSH, ↑FT4, ↑↑FT3,  +ve TRAb, +ve TPO Ab | NR | NR | Carbimazole 40 mg OD + LT4 x12 m. | Continued on LT4. |
| **Thyroiditis** |  |  |  |  |  |  |  |  |  |
| Min L et al (2011) (97) | Melanoma  (n=2) | C1: 48/MNo Hx of ThyD. FHx of ThyD & AutoD NR  C2:28/F  PHx, FHx of ThyD & AutoD NR | C1: Ipi 10mg/ kg x2 doses + bevacizumab (7.5 mg/kg)  C2: Ipi 10mg/kg + bevacizumab 7.5 mg/kg | C1: Hand tremor/ 6wks  Drug D/C?: NR  C2: Tachycardia/ 9wks  Drug D/C?: NR | C1:↓TSH, FT4index↑  ↑↑TPO + ↑TG Ab  3m later ↑TSH  C2:↓TSH, low FT4  ↑TPO + TG Ab  3m later ↑TSH, then normalized. | C1: I^123^ uptake↓  C2: Pet scan enlarged thyroid | NR  NR | C1: No LT4 used  C2: No LT4 used | Both pts recovered with regards to thyroid |
| Thompson BM et al (2013) *  (31) | Melanoma (n=1) | 52/M  PHx, FHx of EndoD & AutoD NR | Ipi dose NR x3 doses | Headache, fatigue, anorexia/12 wks  Drug D/C?: No | ↓TSH, ↑FT4. –ve TSI & TPO Ab.  ↓cortisol.-ve 21-hydroxylase Ab. ↓LH, FSH low NL T | Brain MRI: No metastasis | NR | Steroids & methimazole | Steroids  THyroiditis resolved. |
| Orlov S et al (2015) (103) | Melanoma (n=6) | C1: 51/M  C2: 57/F  C3: 81/F  C4: 42/F  C5: 45/M  C6: 48/F  PHx, FHx of ThyD &AutoD NR in all | Anti-PD-1 2-10mg/kg q3wks. | Transient hyperthyroidism-fatigue +palpitations/3-6 wks  Drug D/C?: NR in all | C1,4: ↓ TSH, ↑FT4 & FT3.  –ve TPO +TG Ab  C2,3,5,6: ↓ TSH, ↑FT4, FT3. +ve TPO +TG Ab.  After 4 wks all became hypothyroid. | NR | NR | β-blockers for 4 wks. Then LT4 for hypothyroidism for all. | Required LT4 replacement |
| Verma I et al (2015) (104) | Lung Ca  (n=1) | 55/F  PHx, FHx of ThyD & AutoD NR | Nivo dose NR q3wks x3 doses | Palpitation, dyspnea,/9 wks  Drug D/C?: Yes | ↓TSH, ↑FT4 & FT3  -ve TSI, TPO Ab  ↑ TG Ab. | Thyroid US NL gland | NR | β-blocker | D/C β-blocker |
| Yu C et al  (2015) (105) | Melanoma  (n=1) | 88/F  PHx, FHx of ThyD & AutoD NR | Surgery, Ipi q3wks x3 doses | Tachycardia, fatigue 6 wks  Drug D/C?: Yes | ↓TSH, ↑FT4 & FT3  -ve TSI, TRAb, TPO & Tg Ab. | Thyroid US small nodules | NR | Iopanoic acid + methimazole  STORM? | Methimazole D/C 3 wks. |
| Ferdousy F et al (2016)* (106) | Melanoma  (n=1) | 49/M  PHx, FHx of ThyD & AutoD NR | Ipi dose NR | Palpitation, diarrhea + fever/3wks after most recent dose  Drug D/C?: NR | ↓TSH ↑FT4, ↑↑FT3 | NR | NR | HD steroids,  PTU, Lugol’s solution, β-blocker  STORM | Recovered |
| Panach K et al (2016)* (107) | Renal cell carcinoma (n=1) | 58/M  PHx, FHx of ThyD & AutoD NR | Nivo dose NR. | Hyperthyroidism symptoms/4 wks  Then hypothyroid symptoms.  Drug D/C?: NR | Baseline: NL TSH & FT4  4 wks: ↓TSH, ↑FT4.  Shortly after,, ↑TSH, ↓FT4 | 4wks: ↓RAI uptake | NR | LT4 | LT4 NR |
| Pena JMM et al (2016)*(108) | AdenoCa of lung (n=1) | 83/F  PHx, FHx of ThyD & AutoD NR | Pembro dose NR q3wks. | Fatigue/3 wks  Then, hypothyroid/5 wks  Drug D/C?: NR | Baseline: NL TSH  3 wks: ↓TSH, NL FT3, FT4.  5 wks: ↑TSH, undetectable FT4.  +ve TG & TPO Ab. | NR | NR | 5 wks: LT4 | LT4 |
| Somasundaram A et al (2016)* (109) | Melanoma (n=1) | 45/F  PHx, FHx of ThyD & AutoD NR | Ipi dose NR x3 doses, then Pembro dose NR x5 doses | Fatigue, weight loss, nausea, vomiting, diarrhea/25 wks  Drug D/C?: NR | 25 wks: ↓TSH, ↑FT3, FT4  -ve TPO & TG Ab & TSI | ThyrUS NL | NR | HD steroids (Thickening of colon wall) | Prednisone taper. |
| McMillen B et al (2016) (110) | Melanoma (n=1) | 24/F  PHx, FHx of ThyD & AutoD NR | I yr interferon + surgery. Then Nivo + Ipi dose NR q3wk | Nausea, vomiting, anxiety goiter proptosis /4 wks  Dug D/C?: Likely Yes | ↓TSH, ↑FT4 & FT3.  -ve TSI,TPO Ab - | Thyroid US Diffuse heterogeneous gland | NR | HD steroid, methimazole, β-blockers  STORM | Recovered.  Required steroids (to be tapered) & methimazole |
| Narita T et al (2016) (111) | Melanoma (n=1) | 65/F  PHx, FHx of ThyD & AutoD NR | Nivo 2m/kg q3wks | Asymptomatic / 6wks  Drug D/C?: NR | ↓TSH, ↑FT4, FT3  TRAb NL. ↑TPO Ab. | Thyroid US Swollen gland | NR | Potassium Iodide | NR |
| Tanaka R et al (2016) (112) | Melanoma (n=2) | C1: 70/F  Hashimoto’s thyroiditis. FHX of ThyD & AutoD NR.  C2:80/M  PHx, FHx of ThyD & AutoD NR | C1:Nivo dose NR | C1: No Sx/ 3wks.  Drug D/C?: No. Hypothyroid at 9 wks;  C2: Tachycardia/6 wks.  Drug D/C?:Yes | C1: ↓TSH, ↑FT4 & FT3. At 9 wks, ↑TSH, ↓FT4 & FT3.  -ve TRAb. +ve TPO & TG Ab  C2: ↓TSH, ↑FT4 & FT3  -ve TRAb, TPO & TG Ab | C1: Thyroid US: Diffuse enlargement & ↑vascularity.  C2;NR | NR | C1: LT4  C2: NR | C1: LT4  C2:NR |
| Besemer B et al (2016) (113) | Melanoma (n=1) | 54/F  PHx, FHx of ThyD & AutoD NR | Ipi 3mg/kg q3 wks | Weakness, heat intolerance/ 6 wks  Drug D/C?: Yes  Then Ipili re-started | ↓TSH, ↑FT4  +ve TPO + TG Ab.  15 days after 3^rd^ dose, pt became hypothyroid. | Thyroid US:  NL thyroid | NR | LT4 | LT4 |
| Yu, D et al (2016)* (114) | Lung adeno Ca (n=1) | 71/M  PHx, FHx of ThyD & AutoD NR | Chemoo D/C AE. Then, Nivo dose NR | Symptoms NR/ 8 wks  Hypothyroid symptoms/16 wks. | Baseline: NL TSH  8 wks: ↓TSH. +ve TPO Ab. TSI –ve.  16 wks: ↑TSH, ↓FT4.  . | NR | NR | 16 wks: LT4 | NR |
| Delivanis DA  et al (2017) (115) | Melanoma (n=12) + Non-small cell lung cancer (n=1)  (n=7/13) | Median age 59yrs  PHx, FHx of ThyD & AutoD NR | Ipi 4mg/kg q4wks x4 doses then Pembro 2 mg/kg q3wks | Sx NR/  C1: 12 wks, C2: 28 wks, C3: 12 wks, C4: 12 wks, C5: 24 wks, C6: 12 wks,  C7: NR  Drug D/C: NR | ↓TSH in all 7.  C4, 5 & 7: ↑FT4  C3: ↑FT3  --ve TRAb all 7  C5-7: Became hypothyroid | NR | NR | 3 cases (C5-7) progressed to hypothyroidism. Needed LT4 | These 3 required LT4. |
| Diamantopoulos PT et al (2017) (116) | Melanoma (n=1) | 62/F  No PHx of ThyrD,  FHx of ThyD & AutoD NR | Nivo 3 mg/kg q3wks | Asymptomatic/8wks  Drug D/C?: No.  At 16 wks, became hypothyroid.  Drug D/C?: Yes | 8^th^ wks:↓ TSH, ↑FT4 & FT3  +ve TPO & TG Ab before Rx.  16 wks: ↑TSH, ↓FT3 & FT4. | NR | 2 | 8 wks: Methimazole  16 wks: LT4 | LT4 replacement |
| Morganstein DL et al (2017) (117) | Melanoma  (n=5) | C1: 71/M  C2:59/M  C3:60/M  C4:61/F  C5:86/M  No PHx of ThyrD, FHx of ThyD & AutoD NR | C1: Nivo  C2: Pembro  C3, 4,: Ipi+Nivo  C5: Ipi | C1: Hyperthyroid/ 10wks  C2: Hyperthyroid/4wks  C3: Hyperthyroid/3 wks  C4: Hyperthyroid/4 wks  C5: Hyperthyroid/6 wks  Drug D/C?: NR | C1: ↓TSH, ↑FT4, FT3  C2: ↓TSH, ↑FT4, FT3  C3: ↓TSH, ↑FT4, FT3  C4: ↓TSH, ↑FT4, FT3  C5: ↓TSH, ↑FT4, FT3  C2&4 became hypothyroid (↑↑TSH) C1,3-5 subclinical hypothyroid (↑TSH)  C1+C5: +ve TPO Ab | All had ↓uptake in Tc^99^ scan & uptake. | NR | No anti-thyroid drugs needed.  C1,2,4,6 needed β-blockers.  C1,2,4,5 needed LT4 | C1,2,4,5 needed LT4 79-466 days after onset. |
| Nandavaram S et al (2017) † (118) | Melanoma (n=1) | 54/F  PHx, FHx of ThyD & AutoD NR | Ipi/dose NR | Asymptomatic/6 wks  Drug D/C?: NR | ↓ TSH, ↑FT4 & FT3.  +ve TPO and TG Ab | Abnormal thyroid scan | NR | NR | NR |
| Osorio JC et al (2017) (119) | Non-small cell lung Ca (n=6) | Age/gender NR  PHx, FHx of ThyD & AutoD NR | Pembro /dose NR | Transient hyperthyroidism/ Onset NR. 6/8 thyroiditis pts became hypothyroid.  Drug D/C?: No | ↓TSH. FT4, FT3 NR,  -ve Thyr Ab | NR | NR | No β-blockers or methimazole needed. | 6 of 8 put on LT4 for hypothyroidism |
| Sweeting AN et al (2017)* (120) | Melanoma (n=5) | C1: 57/F  C2:58/M  C3:73/M  C4:82/M  C5:65/F  PHx, FHx of ThyD & AutoD NR | Pembro / 2mg/kg q3wks | Asymtomatic /  C1: 3wks/Hypothy 9 wks  C2: 3 wks/hypothy 9wks  C3: NR/ hypothyroid 9 wks  C4: 6wks/hypothy 15 wks  C5: NR/hypothyroid 9 wks  Drug D/C?: NR in all | ↓TSH, ↑FT4 + FT3  +ve TPO Ab in C1.  +ve TG Ab in C4. | NR | NR | LT4 9-15 wks later when hypothyroidism developed | LT4. |
| Van Kooten MJ et al (2017) (121) | Non Small cell Lung Ca (n=2) | C1:63/F  No PHx of ThyD & AutoD. Has FHx of ThyD.  C2:71/F  No Hx & FHx of ThyD.  Hx of AutoD NR | Nivo; dose NR | C1: Sweating, weight loss + tachycardia/4wks  Drug D/C?: No  C2: Sweating, weight loss + tachycardia/2wks  Drug D/C?: No | C1: ↓TSH, ↑FT4 and FT3  -ve TPO & TBII Ab.  8 wks after Nivo hypothyroid  C2: ↓TSH, ↑FT4 and FT3  -ve TPO & TBII Ab.  8 wks after Nivo hypothyroid | FDG-PET scan ↑ uptake in both | Nr | Β-blocker and methimazole in both pts | C1 & C2: LT4 |
| Win MA et al (2017) (122) | Melanoma (n=1) | 73/M  PHx, FHx of ThyD NR  No Hx of AutoD | Ipi + Nivo dose NR. | Anxious, tachycardia/ 8 wks  Drug D/C?: NR | ↓TSH, ↑FT4  Undetectable TSI and TRAb  -ve TPO Ab. & wks after ER visit,: ↑TSH,↓FT4 & FT3 | NR | NR | No antithyroid drugs. Put on LT4 when hypothyroid | Put on LT4 in hypothyroid phase 7 weeks after onset. |
| Yamauchi I et al (2017) (123) | C1-3: Melanoma  C4: Breast CA  C5: Lung Ca  (n=5) | C1: 61/F  C2: 72/M  C3: 79/F  C4: 66/F  C5: 63/M  PHx, FHx of ThyD & AutoD NR | C 1-4: Nivo 2mg/kg q3w.  C 5:Nivo 3mg/kg q2w. | Transient hyperthyroidism/  C1: 3 wks. C2:9 wks. C3: 3 wks. C4: 2 wks. C5: 17 wks  Drug D/C: No C1, 4 +5.  Yes - C2 + C3. | C1-5: ↓TSH, ↑FT4, FT3.  -ve TRAb in C1-C4. Not done in C5.  C2: Also had ACTH def and admitted.  Hypothyroid C1: 6 wks, C3 6 wks, C4 3 wks & C5 4 wks. | NR | NR | C2: HD steroids. Other pts needed LT4. | C 1, 3-5 required LT4. |
| Konda B et al (2017) (80) | Renal Cell Carcinoma  (n=1) | 54/M  Hx, FHx of ThyD & AutoD NR | Nivo/ 3mg/kg q2wks | Fatigue/ 12 wks  Drug D/C: NR | ↓TSH; NL FT4 .  5 wks later ↑↑TSH,↓FT4;  ACTH + cortisol NL | NR | NR | LT4 when hypothyroid | LT4 replacement |
| Okiyama N et al (2017) (124) | Melanoma (n=2) | C1:70/F  Hashimoto’s thyroiditis.  FHx of ThyD & AutoD NR  C2:80/M  Hx, FHx of ThyD & AutoD NR | Nivo/dose NR in both cases | C1: fatigue/ 3 wks.  Had +ve TPO Ab at start  Drug D/C?: Yes  C2: Myalgia, hyperthyroid symptoms/6 wks..  Drug D/C?: NR | C1: ↓TSH, ↑FT4 + FT3.  9 wks later ↑TSH, ↓FT4 + FT3.  C2: ↓TSH, ↑FT4 + FT3 | C1: Thyroid US: Goiter + findings consistent with Hashimoto’ s thyroiditis.  C2: NR | NR | C1: LT4  C2: HD steroids | C1: LT4  C2: No LT4 mentioned |
| O’Malley G et al (2017) (125) | C1: Renal cell Carcinoma  C2:Multiple myeloma  C3:Melanoma  C4:Melanoma  C5: Esophageal cancer  (n=5) | C1: 64/M  C2: 71/M  C3: 76/M  C4: 66/M  C5: 50/F  No PHx of ThyD in all. FHX of ThyD & AutoD NR | C1&2: Nivo 3mg/kg  C3: Nivo 1mg/kg + ipi dose NR  C4: Nivo1mg/kg x3m, then 3mg/kg + ipi dose NR  C5: Nivo 3m/kg | C1: Sx NR/6 wks  C2: Sx NR/5 wks  C3: Sx NR/3 wks  C4: Sx NR/12 wks  C5: Sx NR/4 wks  Drug D/C?: NR in all | ↓TSH in C1-5. ↑FT4 in C 1-3. FT4 NR in C4 & 5.  +ve TPO Ab in C2.  +ve TG AB in C2 & C4.  -ve TRAb in all.  ↑ TSH, ↓FT4 in C1: 7 wks;  C2: 8 wks;, C3: 6 wks.  NL TSH in C4.  ↓TSH in C5 lost to FU. | Thyroid US  C1: Hyperemia  C2-4: Heterogeneous echostructure  C5:NL  C3,9: ↑uptake on PET scan. | NR | LT4 for C1-C3.  C4: No Rx  C5: Lost to follow-up | LT4 for C1-C3. |
| Paepegaey AC et al  (2017) (126) | Melanoma  (n=1) | 55/F  PHx, FHx of EndoD & AutoD NR | Surgery, chemo x2, Pembro 2mg/kg q3 wks x10 doses | Palpitations, ↓weight/16 wks  Drug D/C?: Yes at 30 wks.  Hypotension, Hyppoglycemia/34 wks | 16 wks: ↓TSH, ↑FT4 & FT3. -ve TPO, TG & TR Ab.  18 wks: Hypothyroid  34 wks: ↓cortisol, ↑ACTH, Synactin test abn, +ve adrenal Ab. Nl, FSH, LH, PRL, TSH (on LT4), IGF-1 | Thyroid scan no uptake.  42 wks: CT abd: atrophied adrenals. | NR | 18wks: LT4  34 wks: iv HD steroids, then HCT & Florinef | HCT, Fludrocortisone & LT4 |
| Lupu J et al  (2017) (127) | Melanoma  (n=1) | 53/F  PHx, FHx of EndoD & AutoD NR | Ipi 1mg/kg +Nivo 3mg/ kg q3wks | Thyrotoxicosis/6wks  Then hypothyroid.  Weight ↑, polyuria, depression/9wks.  Drug D/C?: Yes at 12 wks.  Anorexia, weakness/16 wks  Wk 22: Drugs re-started. | 6 wks: ↓TSH, ↑FT4 & FT3. -ve thyroid Ab.  Then ↓TSH, ↓FT4 & FT3.  9wks: Sl ↑cortisol.  12 wks: ↑ACTH, ↑cortisol. Abnormal low dose Suppression test. ↑24 hr [cortisol] _u_  ↓LH, estradiol. NL PRL  16 wks: ↓ACTH, cortisol | Thyroid US: Hypoechogenic gland.  MRI: NL pituitary before Rx. At 12 wks: Pituitary enlarged. | NR | Wk6: LT4  Wk 16: HCT & LT4 | HCT & LT4 |
| Alhusseini M et al (2017) † (128) | Adenocarcinoma of lung (n=1) | 65/M  Has Hx of T2DM. FHx of DM & Hx of AutoD NR. | Ipi+Pembro dose NR | DKA Sx/ 3 wks  Drug D/C?: Yes for Ipili & No for Pembro | ↑A1c, ↑Glu, ↓pH, ↓ C-peptide.+veGAD65, islet, insulin Ab.  ↓TSH, ↑FT4. -ve TSI, +ve TPO Ab | Thyroid scan: ↓ uptake. | NR | DKA Rx, insulin | Insulin + LT4 |
| Reddy SC et al (2017)*  (129) | Non-small cell Lung cancer (n=1) | 66/M  PHx of DM. FHx of DM and AutoD NR | Nivo dose NR. | DKA Sx/4wks  Drug D/C –Yes and gemcitadine started | 4 wks: ↑Glu, ↓pH, ↓C-pep. +ve GAD 65 Ab.  ↓TSH, ↑FT4, FT3. +ve TPO Ab; TSI –ve. | ThyUS: heterogeneous gland | NR | Insulin & fluids.  Methimazole & β-blockers, then high dose steroids | Insulin |
| Sakurai K et al  2018 (130) | Renal cell carcinoma (n=1) | 68/M  No PHx ,FHx of DM. Had Hashimoto’s thyroiditis | Surgery, chemo- & radiation therapy. Then Nivo dose NR. | Palpitationa & fatigue/3 wks  Hyperglycemia Symptoms / 14 wks  Drug D/C?: No | 3wks: ↓TSH, ↑FT$ & FT3.  +ve TPO & TG Ab, -ve TRAb.  9wks: Hypothyroid  14 wks: ↑A1c, 3↑Glu, NL pH, ↑β-OH butyr, ↓ C-peptide. - ve GAD65, IA-2, IAA, ZnT18 Ab.  HLADRB1*09:01-DQB1*03.03  NL adrenal tests. | Thyroid scan: No uptake.  Abdomina CT:Pancreas NL | NR | LT4 & Insulin | Lt4 & insulin |

**(b) Hypothyroidism**

| **Authors** | **Cancer/**  **# of patients** | **Age/Gender**  **Pertinent Med History** | **Drug / dose** | **Clinical Sx /onset after 1^st^ dose**  **Drug D/C?:** | **Laboratory investigations** | **Imaging** | **CTCAE Grade** | **Treatment** | **Outcome** |
| --- | --- | --- | --- | --- | --- | --- | --- | --- | --- |
| **Primary hypothyroidism** |  |  |  |  |  |  |  |  |  |
| Min L et al  (2014) (131) | Melanoma (n=1) | 46/M  PHx, FHx of ThyD and AutoD NR | Surgery, Ipi 10 mg/kg q3wks | Fatigue, myalgia, tender muscles/15 wks  Drug D/C?: NR | ↑↑↑TSH, undetectable FT4. CK and SGLT ↑  Cortisol NL | NR | NR | LT4 | LT4 |
| Yun S et al  (2015) (60) | Melanoma (n=1) | 59/M  No Hx of AutoD.  PHx, FHx of Thy D NR. | Ipi 3 mg/kg q3wk x4 doses | Chest pain, dyspnea/24  wks.  Drug D/C: NR | ↑TSH ↓FT4 (slight)  ↓cortisol, ↓ACTH | NR | NR | HD steroids  No LT4 | Recovered. No LT4.  Subclinical hypothyroidism |
| Mellati M  et al (2015) (132) | Sarcomatoid Squamous cell Ca of jaw  (n=1) | 66/F  PHx, FHx of ThyD & AutoD  NR | PDL-1 Ab q2 wks x 3 doses | DKA Symptoms after 7 wks  Drug D/C ?: NR . | ↑TSH, ↓FT3,  +ve TPO Ab  ↑ A1c,↑Glu, ↓ C-pep,  +ve GAD65 Ab,  DR3-DR2/DR4-DQ8 | NR | NR | Insulin  LT4 NR | Insulin dependent  LT4 NR  Subclinical hypothyroidism |
| Narita T  et al (2016) (111) | Melanoma (n=1) | 70/M  PHx, FHx of ThyD & AutoD NR | Chemo, Surgery, Chemo again, then Nivo 2mg/kg x4 doses | Asymptomatic/7 weeks.  Drug D/C?: NR | ↑TSH, ↓FT4  ↑↑TPO & TG Ab | Thyroid US swollen gland | NR | LT4 | Discharged to hospice on LT4. |
| Tanaka R  et al (2016) (112) | Melanoma (n=1) | 64/M  PHx, FHx of ThyD and AutoD NR | Nivo/dose NR | Clinical symptoms NR/ 15 wks changed from subclinical to overt hypothyroid  Drug D/C?: NR | Before NL FT3 & FT4. After 9 wks, TSH ↑ and ↓FT4 & FT3.  TPO Ab +ve. | NR | NR | LT4 | LT4 |
| Khan U et al  (2017) (133) | Lung Squamous cell carcinoma  (n=1) | 53/F  No PHx of thyD & AutoD.  FHx of thyD NR. | Chemo, Surgery,  then Nivo dose NR x10 doses | Swollen tongue, weakness, periorbital edema / onset NR.  Drug D/C?: Yes | ↑↑TSH,  ↓↓FT4 | NR | NR | LT4 iv x3 days then PO | Recovered on LT4 |
| Li L et al  (2017) (134) | Lung Squamous cell carcinoma  (n=1) | 63/M.  PHx, FHx of ThyD & AutoD NR | Chemo, radiation; then  Nivo dose NR | Presented with DKA 4 wks after Nivo initiation. Then 12 wks after Nivo hypothyroid  Drug D/C?: NR | ↑ A1c,↑Glu, ↓ C-pep,  +ve GAD65 Ab,  TSH NL but ↑TPO Ab 27 days after NIVO. 12 wks later ↑TSH. | NR | NR | DKA Rx, then insulin.  When hypothyroid, LT4 started | Deceased after 5^th^ dose of Nivo. |
| Oda T et al  (2017) † (82) | Melanoma  (n=1) | 85/M  PHx, FHx of ThyD & AutoD NR | Nivo 2mg/kg q3 wks | AV block/12 wks  Fatigue, hypotension /60wks  Drug D/C?: Yes | At 20 wks ↓FT4, ↑TSH. ↓ACTH & cortisol. CRF test abnormal. Other pituitary hormones NL | NR | NR | Oral HCT | Oral HCT  Subclinical hypothyroidism |
| Imafuku K  et al (2017) † (135) | Melanoma (n=1) | 78/M  PHx + FHx of ThyD & AutoD NR. | Nivo 2mg/kg q3wks x3 doses | Asymptomatic/ 23 wks.  Drug D/C?: Nivo D/C on day 61 because of CHF. | Day 102 after NIVO D/C ↑TSH and ↓FT4 & FT3. | NR | NR | LT4 | LT4 |
| O’Malley G  et al (2017) (125) | C1:Lung Ca (n=1)  C2:Lung cancer (n=1)  C3: Bladder cancer (n=1)  C4: Multiple Myeloma(n=1) | C1: 73/F  C2: 67/F  C3: 71/F  C4: 60/F  No PHx of ThyD in all. FHX of ThyD & AutoD NR | C1-C3: Nivo 3mg/kg  C4: Nivo 1mg/kg + Ipi | Clinical symptoms NR  C1:16 wks  C2: 12wks  C3: 7 wks  C4: 36 wks  Drug D/C?: NR in all. | ↑TSH, ↓FT4 in all  C3 & C4: +ve TPO Ab  C3: +ve TG Ab | Thyroid US  C1-C3: heterogeneous echostructure | NR | LT4 for all | LT4 for all. |
| Delivanis DA e  t al (2017) (115) | Melanoma 12 + NSCLA 1  (n=6/13) | Median age 59yrs | Ipi 4mg/kg q4wks x4 doses then Pembro 2 mg/kg q3wks | Clinical symptoms NR.  C1: NR  C2: 12 wks  C3: 8 wks  C4: NR  C5: 20 wks  C6: 12 wks (subclin hypothyroid) | ↑TSH in C1-C6)  ↓FT4 in C1,2, 4-6.  +ve TPO Ab C1,2 & 6. | ↑^18^FDG-PET/CT in C2,5 &^. | NR | LT4 for C1-5. | LT4 for C1-5. |
| Guaraldi F et al  (2017) (136) | Melanoma  (n=5) | Mean age 64 yrs  C1: F  C2: M  C3: F  C4: M  C5: F | Ipi 3mg/kg q3wks x4, then Pembro 2mg/kg q3wks (C:1,2,4)or Nivo 2mg/kg q3wks (C; 3,5) | Hypothyroidism Sx/  13 wks  Drug D/C?: No in all. | C1: ↑TSH, NL FT4  C2-5: ↑TSH, ↓FT4 | NR | NR | LT4 | LT4 |
| DiLucca G et al (2017) *  (137) | Renal cell carcinoma (n=1)  Squamous cell lung cancer (n=1) | C1: 75/F  C2:69/M  PHx + FHx of ThyD & AutoD NR.in both | C1: Chemo, then nivo 3mg/kg q2wks  C2: Chemo, then nivo 3mg/ kg q2wks | C1: Symptoms NR/12 wks  C2: Edema, asthenia, somnolence/? wks.  Drug D/C?: Yes in both. | C1: ↑TSH, ↓FT4. +ve TPO Ab.  C2: ↑TSH, ↓FT4. +ve TPO & TG Ab. | NR | NR | Both: LT4 | Both LT4 |
| Torimoto K et al  (2017) † (138) | Lung cancer (n=1) | 63/M  PHx, FHx of ThyD & AutoD NR. | Nivo dose NR x2 doses, then D/C because of no effect | Hypothyroid symptoms/ 12 weeks  DrugD/C: After 2 doses because of no effect | Baseline: NL TSH & FT4 .+ve TPO/TG Ab.  12 wks: ↑TSH,↓FT4.  ↑↑TPO/TG Ab.  TSI & TRAb –ve. | Baseline Thy US NL  12 wks: ThyUS atrophy & ↓  echogenicity | NR | LT4 at 12 wks | LT4 |
| Bhalla S et al (2017) (90) | Melanoma  (n=1) | 70/F  PHx, FHx of EndoD & AutoD NR | Nivo + Ipi dose NR x6 doses. followed by Nivo dose NR. | Fatigue, nausea, vomiting/14 wks.↓BP, syncope/18 wks  Drug D/C? : NR | 17wks: ↑TSH, ↓FT4  18 wks:↓ACTH, cortisol,LH,FSH  [Na+] | MRI pituitasry: Enhancement | NR | 17 wks:LT4  18wks: HD steroids | NR |
| Zeng MF et al (2017) (93) | Renal cell carcinoma  (n=1) | 54/M  Has T2DM  FHx of EndoD & AutoD NR | Chemotherapy and Radiotherapy. Then,  Nivo 2mg/kg q2wk | No hypothyroid symptoms/  8 wks  Hypoglycemia/ 24 wks  Drug D/C?: Yes | 8 wks:↑TSH, ↓FT4, ↓FT3,  ↑TPO, ↑TG Ab  24 wks: ↓cortisol, low ACTH, ↓DHEAS.  NL LH, FSH, T & PRL | Not done | NR | LT4, HCT | LT4, HCT |
| **Thyrotoxicosis**  **progressing to hypothyroidism** |  |  |  |  |  |  |  |  |  |
| Orlov S et al  (2015) (103) | Melanoma (n=6) | C1: 51/M  C2: 57/F  C3: 81/F  C4: 42/F  C5: 45/M  C6: 48/F  PHx, FHx of ThyD & AutoD NR in all | Anti-PD-1  2-10mg/kg q3wks. | Transient fatigue +palpitations/3-6 wks  Drug D/C?: NR in all | C1,4: ↓ TSH, ↑FT4 & FT3.  -ve TPO +TG Ab  C2,3,5,6: ↓ TSH, ↑FT4 & FT3.  +ve TPO +TG Ab.  After 4 wks all became hypothyroid. | NR | NR | β-blockers for 4 wks. Then LT4 for hypothyroidism for all. | Required LT4 replacement |
| Lowe JR  et al (2016) (76) | Melanoma  (n=1) | 54/M  PHx, FHx of ThyD & AutoD NR. | Surgery, Nivo 1mg/kg q3wks+Ipi 3mg/kg/  (Doses of  Check-Mate 069 CT) | Tachycardia + hot flashes./ 5 wks  (6 wks later 2^nd^ dose given). 2wks after 3^rd^ dose, DKA Sx/16 weeks  Hypotensive  Drug D/C ?: Yes | 5 wks: ↓TSH, ↑FT4, FT3 & +ve TRAb.  8 wks: ↑TSH &↓FT4 +FT3  2 wks post 3^rd^ dose,  Glu, A1c NR. Β-OHB↑, ↓ C-pep., +ve GAD65 Ab.  ↓ACTH, cortisol & T  ACTH stim test abn | MRI pituitary: NL | NR | HD steroids + β blocker. When ↑TSH, Lt4 started  Insulin for DKA.  HD steroids for hypopituitarism | Insulin-dependent  LT4 NR |
| Besemer B  et al (2016) (113) | Melanoma (n=1) | 54/F  PHx, FHx of ThyD & AutoD NR | Ipi/ 3mg/kg q3 wks | Weakness, heat intolerance/ 6 wks  Drug D/C?: Yes  Then Ipili re-started | ↓TSH, ↑FT4  +ve TPO + TG Ab.  8 wks: Patient became hypothyroid. | Thyroid US:  NL thyroid | NR | LT4 | LT4 |
| Panach K et al *  (2016) (107) | Renal cell carcinoma (n=1) | 58/M  PHx, FHx of ThyD & AutoD NR | Nivo dose NR. | Hyperthyroidism symptoms/4 wks  Then hypothyroid symptoms.  Drug D/C?: NR | Baseline: NL TSH & FT4  4 wks: ↓TSH, ↑FT4.  Shortly after, ↑TSH, ↓FT4 | 4wks: ↓RAI uptake | NR | LT4 | LT4 NR |
| Pena JMM et al (2016) *  (108) | Lung adeno- Carcinoma (n=1) | 83/F  PHx, FHx of ThyD & AutoD NR | Pembro dose NR q3wks. | Fatigue/3 wks. Then, hypothyroid/5 wks  Drug D/C?: NR | Baseline: NL TSH  3 wks: ↓TSH, NL FT3, FT4.  5 wks: ↑TSH, undetectable FT4.  +ve TG & TPO Ab. | NR | NR | 5 wks: FT4 | FT4 |
| Yu, D et al (2016) *  (114) | Lung adeno Ca (n=1) | 71/M  PHx, FHx of ThyD & AutoD NR | Chemo. Then, Nivo dose NR | Symptoms NR/ 8 wks  Hypothyroid symptoms/16 wks. | Baseline: NL TSH  8 wks: ↓TSH. +ve TPO Ab. TSI –ve.  16 wks: ↑TSH, ↓FT4.  . | NR | NR | 16 wks: LT4 | NR |
| Delivanis DA  et al (2017)  (115) | Melanoma (n=12) + Non-small cell lung cancer (n=1)  (n=7/13 thyroiditis; 3/7 hypothyroid) | Median age 59yrs  PHx, FHx of ThyD & AutoD NR | Ipi 4mg/kg q4wks x4 doses then Pembro 2 mg/kg q3wks | Sx NR/  C1: 3m, C2: 7m, C3: 3m,  C4: 3m, C5: 6m, C6: 3 m,  C7: NR  Drug D/C: NR | ↓TSH in all 7.  C4, 5 & 7: ↑FT4  C3: ↑FT3  --ve TRAb all 7  C5-7: Became hypothyroid | NR | NR | 3 cases (C5-7) progressed to hypothyroidism. Needed LT4 | These 3 required LT4. |
| Diamantopoulos  PT et al (2017)  (116) | Melanoma (n=1) | 62/F  No PHx of ThyrD, FHx of ThyD & AutoD NR | Nivo 3 mg/kg q3wks | Asymptomatic/8wks  Drug D/C ?: No.  At 16 wks, became hypothyroid.  Drug D/C ?: Yes | 8^th^ wks:↓ TSH, ↑FT4 & FT3  +ve TPO & TG Ab before Rx.  16 wks: ↑TSH, ↓FT3 & FT4. | NR | 2 | 8 wks: Methimazole  16 wks: LT4 | LT4 replacement |
| Morganstein DL  et al (2017) (117) | Melanoma  (n=6)  2 hypothyroid  3 subclinical hypothyroid | C1: 71/M  C2:59/M  C3:60/M  C4:61/F  C5:86/M  C6:49/F  No PHx of ThyrD, FHx of ThyD & AutoD NR | C1: Nivo  C2: Pembro  C3, 4,6: Ipi+Nivo  C5: Ipi | C1:Hyperthyroid/ 10wks  C2: Hyperthyroid/4wks  C3: Hyperthyroid/3 wks  C4: Hyperthyroid/4 wks  C5: Hyperthyroid/6 wks  C6: Hyperthyroid /7 wks  Hypothyroid onset NR  Drug D/C?: NR | C1: ↓TSH, ↑FT4, FT3  C2: ↓TSH, ↑FT4, FT3  C3: ↓TSH, ↑FT4, FT3  C4: ↓TSH, ↑FT4, FT3  C5: ↓TSH, ↑FT4, FT3  C6: ↓TSH, ↑FT4, FT3  C2,4: became hypothyroid  (↑TSH); C1,3-5 subclinical hypothyroid.  C1+C5: +ve TPO Ab | All had ↓uptake in Tc^99^ scan & uptake. | NR | No anti-thyroid drugs needed.  C1,2,4,6 needed β-blockers.  C1,2,4,5 needed LT4 | C1,2,4,5 needed LT4 79-466 days after onset. |
| Osorio JC  et al (2017) (119) | Non-small cell lung Carcinoma (n=8) | Age/gender NR  PHx, FHx of ThyD & AutoD NR | Pembro /dose NR | Transient hyperthyroidism/ Onset NR.  6/8 thyroiditis pts became hypothyroid. Onset NR  Drug D/C?: No | ↓TSH, FT4, FT3 NR,  -ve Thyr Ab | NR | NR | No β-blockers or methimazole needed. | 6 of 8 put on LT4 for hypothyroidism |
| Sweeting AN  et al (2017)* (120) | Melanoma (n=5) | C1: 57/F  C2:58/M  C3:73/M  C4:82/M  C5:65/F  PHx, FHx of ThyD & AutoD NR | Pembro / 2mg/kg q3wks | Asymtomatic /  C1: 3wks/Hypothy 9 wks  C2: 3 wks/hypothy 9wks  C3: NR/ hypothyroid 9 wks  C4: 6wks/hypothy 15 wks  C5: NR/hypothyroid 9 wks  Drug D/C?: NR in all | ↓TSH, ↑FT4 + FT3  +ve TPO Ab in C1.  +ve TG Ab in C4. | NR | NR | LT4 9-15 wks later when hypothyroid | LT4. |
| Van Kooten MJ  et al (2017) (121) | Non-Small cell Lung Carcinoma (n=2) | C1:63/F  No PHx of ThyD & AutoD. Has FHx of ThyD.  C2:71/F  No Hx & FHx of ThyD.  Hx of AutoD NR | Nivo; dose NR | C1: Sweating, weight loss + tachycardia/4wks  8 wks after Nivo hypothyroid  Drug D/C?: No  C2: Sweating, weight loss + tachycardia/2wks  8 wks after Nivo hypothyroid  Drug D/C?: No | C1: ↓TSH, ↑FT4 and FT3  -ve TPO & TBII Ab.  C2: ↓TSH, ↑FT4 and FT3  -ve TPO & TBII Ab. | FDG-PET scan ↑ uptake in both | NR | Β-blocker and methimazole in both pts | C1 & C2: LT4 |
| Win MA  et al (2017) (122) | Melanoma (n=1) | 73/M  PHx, FHx of ThyD & AutoD NR | Ipi + Nivo dose NR. | Anxious, tachycardia/ 8 wks  Drug D/C?: NR | ↓TSH, ↑FT4  Undetectable TSI and TRAb  -ve TPO Ab. & wks after ER visit,: ↑TSH,↓FT4 & FT3 | NR | NR | No anti-thyroid drugs. Put on LT4 when hypothyroid | Put on LT4 in hypothyroid phase 7 weeks after onset. |
| Yamauchi I  et al (2017) (123) | C1-3: Melanoma  C4: Breast CA  C5: Lung Ca  (n=5) | C1: 61/F  C2: 72/M  C3: 79/F  C4: 66/F  C5: 63/M  PHx, FHx of ThyD & AutoD NR | C 1-4: Nivo 2mg/kg q3w.  C 5:Nivo 3mg/kg q2w. | Transient hyperthyroidism/  C1: 3 wks. C2:9 wks. C3: 3 wks. C4: 2 wks. C5: 17 wks  Drug D/C: No -C1, 4 & 5.  Yes - C2 + C3. | C1-5: ↓TSH, ↑FT4, FT3.  -ve TRAb in C1-C4. Not done in C5.  C2: Also had ACTH deficiency; was admitted.  Hypothyroid C1: 6 wks, C3 6 wks, C4 3 wks & C5 4 wks. | NR | NR | C2: HD steroids. Other pts needed LT4. | C 1, 3-5 required LT4. |
| Konda B  et al (2017) (80) | Renal Cell Carcinoma  (n=1) | 54/M  PHx, FHx of ThyD & AutoD NR | Nivo/ 3mg/kg q2wks | Fatigue/ 12 wks  Drug D/C?: NR | 12 wks:↓TSH; NL FT4 .  17 wks::↑TSH,↓FT4;  ACTH + cortisol NL | NR | NR | LT4 when hypothyroid | LT4 replacement |
| Okiyama N  et al (2017) (124) | Melanoma (n=2) | C1:70/F  Has Hashimoto’s thyroiditis.  FHx of ThyD & AutoD NR  C2:80/M  PHx, FHx of ThyD & AutoD NR | Nivo/dose NR in both cases | C1: fatigue/ 3 wks.  Had +ve TPO Ab at start  Drug D/C ?: Yes  C2: Myalgia, Symp-toms hyperthyroidism/6 wks..  Drug/C ?: NR | C1: ↓TSH, ↑FT4 + FT3.  9 wks later ↑TSH, ↓FT4 + FT3.  C2: ↓TSH, ↑FT4 + FT3  Then ↓FT4 & FT3 | C1: Thyroid US: Goiter + findings consistent with Hashimoto’ thyroiditis.  C2: NR | NR | C1: LT4  C2: HD steroids | C1: LT4  C2: No LT4 mentioned |
| O’Malley G  et al (2017) (125) | C1: Renal cell Carcinoma  C2: Multiple myeloma  C3: Melanoma | C1: 64/M  C2: 71/M  C3: 76/M  No PHx of ThyD in all. FHX of ThyD & AutoD NR | C1&2: Nivo 3mg/kg  C3: Nivo 1mg/kg + ipi dose NR | C1: Symptoms NR/6 wks  C2: Symptoms NR/5 wks  C3: Symptoms NR/3 wks  Drug D/C ?: NR in all | ↓TSH,↑FT4 in C 1-3. .  +ve TPO Ab in C2.  +ve TG Ab in C2 & C3  -ve TRAb in all.  ↑ TSH, ↓FT4 in C1: 7 wks;  C2: 8 wks;, C3: 6 wks.. | Thyroid US  C1: Hyperemia  C2: Heterogeneous echostructure  C3: ↑uptake on PET scan | NR | LT4 for C1-C3. | LT4 for C1-C3. |
| Paepegaey AC et al  (2017) (126) | Melanoma  (n=1) | 55/F  PHx, FHx of EndoD & AutoD NR | Surgery, chemo x2, Pembro 2mg/kg q3 wks x10 doses | Palpitations, ↓weight/16 wks  Drug D/C ?: Yes at 30 wks.  Hypotension, Hyppoglycemia/34 wks | 16 wks: ↓TSH, ↑FT4 & FT3. -ve TPO, TG & TR Ab.  18 wks: Hypothyroid  34 wks: ↓cortisol, ↑ACTH, Synactin test abn, +ve adrenal Ab. Nl, FSH, LH, PRL, TSH (on LT4), IGF-1 | Thyroid scan no uptake.  42 wks: CT abd: atrophied adrenals. | NR | 18wks: LT4  34 wks: iv HD steroids, then HCT & Florinef | HCT, Fludrocortisone & LT4 |
| Lupu J et al  (2017) (127) | Melanoma  (n=1) | 53/F  PHx, FHx of EndoD & AutoD NR | Ipi 1mg/kg +Nivo 3mg/ kg q3wks | Thyrotoxicosis/6wks  Then hypothyroid.  Weight ↑, polyuria, depression/9wks.  Drug D/C ?: Yes at 12 wks.  Anorexia, weakness/16 wks  Wk 22: Drugs re-started. | 6 wks: ↓TSH, ↑FT4 & FT3. -ve thyroid Ab.  Then ↓TSH, ↓FT4 & FT3.  9wks: Sl ↑cortisol.  12 wks: ↑ACTH, ↑cortisol. Abnormal low dose Suppression test. ↑24 hr [cortisol] _u_  ↓LH, estradiol. NL PRL  16 wks: ↓ACTH, cortisol | Thyroid US: Hypoechogenic gland.  MRI: NL pituitary before Rx. At 12 wks: Pituitary enlarged. | NR | Wk6: LT4  Wk 16: HCT & LT4 | HCT & LT4 |
| Guaraldi F et al  (2017) (136) | Melanoma  (n=2) | Mean age 64 yrs  C1: M  C2: M | Ipi 3mg/kg q3wks x4, then Pembro 2mg/kg q3wks (C:2) or Nivo 2mg/kg q3wks (C:1) | Hypothyroidism Sx/  13 wks  Drug D/C?: No in both | ↓TSH, ↑FT4 then ↑TSH,↓FT4. | NR | NR | LT4 | LT4 |
| Sakurai K et al  2018 (130) | Renal cell carcinoma (n=1) | 68/M  No PHx ,FHx of DM. Had Hashimoto’s thyroiditis | Surgery, chemo- & radiation therapy. Then Nivo dose NR. | Palpitationa & fatigue/3 wks  Hyperglycemia Symptoms / 14 wks  Drug D/C?: No | 3wks: ↓TSH, ↑FT$ & FT3.  +ve TPO & TG Ab, -ve TRAb.  9wks: Hypothyroid  14 wks: ↑A1c, 3↑Glu, NL pH, ↑β-OH butyr, ↓ C-peptide. - ve GAD65, IA-2, IAA, ZnT18 Ab.  HLADRB1*09:01-DQB1*03.03  NL adrenal tests. | Thyroid scan: No uptake.  Abdomina CT:Pancreas NL | NR | LT4 & Insulin | Lt4 & insulin |

C: Case; *: Abstract; †: Letter to the editor; M: Male; F: Female; NR: Not reported; ND: Not done; NL: Normal;

PHx: Personal history; FHx: Family history; ThyD: Thyroid disease; AutoD: Autoimmune disease;

Ipi: Ipilimumab, Nivo: Nivolumab; Pembro: Pembrolizumab; Tremeli: Tremelimumab; PD-1: Programmed Cell Death-1; PDL-1: Programmed Death Ligand-1;

Sx: Symptoms; D/C: Discontinued; q:every; wks: weeks; NL: Normal; ↓: Decreased; ↑: Increased; Abn: Abnormal;

ACTH: Adrenocorticotrophic hormone; CRH: Corticotropin-releasing hormone; LH: Luteinizing hormone; FSH: Follicle stimulating hormone; PRL: Prolactin; GH: Growth hormone; IGF1: Insulin growth factor-1; TSH: Thyroid stimulating hormone; FT4: Free thyroxine; FT3: Free triiodothyronine; TPO: Thyroperoxidase antibodies (Ab); TG: Thyroglobulin Ab; TRAb: Thyroreceptor Ab; TSI: Thyroid stimulating immunoglobulin; Glu: Glucose; DKA: Diabetic ketoacidosis; C-pep: C-peptide; A1c: glycated hemoglobin; GAD 65: Glutamic acid decarboxylase 65; IA-2: Islet Antigen 2; IAA: Insulin auto-antibody; ZnT8: Zinc Transporter 8;

CTCAE: Common terminology criteria for adverse events; RAI: radioactive iodine; MRI: magnetic resonance imaging; CT: Computerized tomography; PET: Positron emission tomography; US: Ultrasound;

Rx: Treatment. LT4: levothyroxine; Chemo: Chemotherapy; HD steroids: High dose steroids.
