# Supplementary material for: Spectrum of immune checkpoint inhibitors-induced endocrinopathies in cancer patients: a scoping review of case reports
Source: Clin Diabetes Endocrinol. 2019 Jan 22;5:1. doi: 10.1186/s40842-018-0073-4 (PMC6343255; doi:10.1186/s40842-018-0073-4)
Supplement: Supplementary file 5 — Appendix 5. Cases of Immune Checkpoint Inhibitors-Induced Endocrinopathies –Diabetes Mellitus. (DOCX 41 kb) [file 40842_2018_73_MOESM5_ESM.docx]

**Appendix 5: Cases of Immune Checkpoint Inhibitors-Induced Endocrinopathies –**

**Diabetes Mellitus**

| **Authors**  **(Year)**  **(Refernece)** | **Cancer/# of patients** | **Age/Gender**  **Pertinent History** | **Drug/dose** | **Clinical Sx/onset after 1^st^ dose**  **Drug D/C ?:** | **Laboratory investigations** | **Imaging** | **CTCAE**  **Grade** | **Treatment** | **Outcome** |
| --- | --- | --- | --- | --- | --- | --- | --- | --- | --- |
| Gaudy C  et al (2015)  (139) | Melanoma  (n=1) | 44/F  No PHx of DM.  FHx of DM and AutoD NR. | Pembro /dose NR | DKA Sx /8 weeks  Drug D/C?: Yes | ↑A1c ; 5↑Glu, ↓ C-pep,↓pH, ↓HCO3,-ve GAD + IA2 Ab.  ↑lipase | Abd CT: NLpancreas ↓lesions | NR | Insulin  Fulminant T1D (FTID) | Insulin-dependent |
| Hughes J  et al (2015)  (140) | C1:: Melanoma  (n=1)  C2: Non-Small Cell lung cancer  (n=1)  C3: Renal Cell Carcinoma (n=1)  C4: Squamous Cell Carcinoma of lung (n=1)  C5: Melanoma (n=1) | C1: 55/F  C2: 83/F  C3: 63/M  C4: 58/M  C5: 64/F  C1,C5: AutoD (thyroid)  C4: T2DM  C1-3,5: No PHx of DM  All: No FHx of DM | C1: Nivo + Ipi/ doses NR + steroids  C2-4: Nivo/ dose NR + chemo  C5: Pembro/ Dose NR | C1: DKA / 20 wks  C2: DKA/ 4 wks  C3: DKA/ 16 wks  C4: DKA/ 1wk  C5: ketonuria/<4 wks  Drug D/C ?: C1-5 NR | C1: ↑A1c,3↑Glu,↓C-pep  C2: 2↑ A1c; 2↑Glu,↓C-pep  C3: 2↑ A1c, ↑Glu, NL C-pep;  ↑CD8-T cells  C4: 4↑A1c; 4↑Glu,↓C-pep, ↑CD8-T cells  C5: ↑A1c; 4↑Glu,↓C-pep  C2-4: +ve GAD65 Ab  C3 also +ve ICA512 + IAA Ab  HLA: C1-3: A2.1,DR4;  C4 A2.1;C5 DR4. | NR | NR | Insulin | NR |
| Martin-Liberal J  et al (2015)  (141) | Melanoma  (n=1) | 54/F  PHx of DM NR  FHx of DM: Yes  Hx of AutoD: NR | Ipi 3mg/kg x 4 doses,8 m later Pembro 2 mg/kg x3 weekly | DKA Sx/9 wks after Pembro  Drug D/C ?: NR | A1c + Glu levels NR  +ve GAD65 Ab  HLA A2 DR4 DQ8glu | Tumor response | NR | Insulin | Insulin- dependent |
| Mellati M  et al (2015) (132) | C1: Lung adenocarcinoma  (n=1)  C2: Sarcomatoid Squamous cell carcinoma of jaw (n=1) | C1: 70/M  No PHx of DM  FHx DM, AutoD: NR  C2: 66/F  No PHx, FHx of DM.  Hx AutoD NR | C1: PDL-1 Ab q3 wks x5 doses  C2: PDL-1 Ab q2 wks x 3 doses | C1:↑↑ Glu/ 15 wks. DKA 10 days later.  C2: DKA Sx after 7 wks  Drug D/C ?: NR in both. | C1: 4↑A1c,3↑ Glu, ↓C-pep, pH NR, ↑ anion gap.-ve GAD Ab. NL TSH, FT3  C2:4↑ A1c,4↑Glu, ↓ C-pep, ↓pH, ↑anion gap.+ve GAD65 Ab, ↑TSH,↓FT3, +ve TPO Ab  DR3-DR2/DR4-DQ8 | NR | NR | C1: initially Metformin;  10 days later insulin.  C2: insulin | C1:insulin dependent.  C2: insulin dependent, |
| Aleksova J  et al  (2016) (142) | Melanoma (n=1) | 61/M  No PHx & FHx of T1DM or AutoD. | Surgery, chemo, Ipi/ 3mg/kg x4 doses.  5 weeks later Pembro /2mg/kg q2wks x 6wks | DKA Sx/6 wks after start of Pembro  Drug D/C ?: Yes | DKA; ↑A1c, 2↑Glu, ↑(ketones)_s_,↓pH, ↓ C-pep, -ve GAD65 & IA2 Ab. ↓TSH ↑T4, NL T3, -ve Thy Ab  Pituitary & adrenal function NL | NR on pancreas. | NR | DKA Rx, then Insulin  HD steroids x10 days to reverse T1D failed. | Insulin dependent |
| Akturk H et al (2016)* (143) | Renal Cell Carcinoma  (n=1) | 70/M  No PHx & FHx of DM. Hx of AD NR. | Nivo 300 mg/kg  q 2 wks x3 doses | DKA Sx/6 weeks  Drug D/C ?: NR | 2↑A1c, 4↑GLU,↓pH, ↑anion gap,↓ C-pep,-ve GAD65 Ab.  Lipase + amylase NL | Abd CT:NL pancreas | NR | DKA Rx & Insulin | Insulin dependent |
| Hansen E  et al (2016)† (144) | Melanoma  (n=1) | 58/M  PHx & FHx of DM & AutoD NR. | Pembro/2 mg/kg q 3 weeks x 17 doses | ↑Glu Sx /51 weeks.  Drug D/C ?: Yes Then,deframenib and trametinib. | 4↑A1c, 4↑GLU, NL C-pep, +ve GAD65 Ab.  Day 55 after Pembro D/C Glu normalized & Day 81 Insulin D/C. | NR | NR | Insulin,  No steroids | Insulin discontinued 81 days after pembro stopped |
| Hoffman Let al (2016) (145) | Melanoma  (n=3)  Unknown  (n=1) | C1: 70/F  C2: 78/F T2DM diet  C3: 58/F  C4: 40/F  C1+3: No PHx DM  C2: 78/F T2DM diet  All no FHx DM & AutoD | C1: Nivo  C2: Ipi then Nivo  C3: Ipi then Pembro  C4: Ipi then Pembro | C1: ↑GluSx /6 wks. Drug D/C?: No  C2: DKA Sx/4 wks  C3: ↑Glu Sx /3 wks  C4: NR/6 wks  C2-4: Drug D/C?: NR | A1c + Glu NR in all.  C2-4: ↓ C-peptide  C2 & C3: +ve GAD65 & !A2 Ab  C4:DM tests NR | NR | C1: 3  C2: 3  C3: 3  C4: 3 | C1: Insulin  C2: insulin  C3: Insulin  C4: Insulin | NR in 1/4, other 3/4  insulin-dependent |
| Lowe JR et al (2016) (76) | Melanoma  (n=1) | 54/M  PHx, FHx of ThyD & AutoD NR. | Nivo 1mg/kg q3wks+Ipi 3mg/kg/ (Doses of  Check-Mate 069 protocol. | Tachycardia + hot flashes./2weeks after 1^st^ dose  No hypothyroid Sx/ 6 wks later 2^nd^ dose.  Weakness, nausea, vomiting/ 2wks after 3^rd^ dose, (DKA Sx/16 weeks)  Drug D/C ?: Yes (also had hepatitis & colitis) | 2 wk s post 1^st^ dose, ↓TSH, ↑FT4,FT3., +ve TRAb.  +ve anti-microsomal Ab  Then ↑TSH, ↓FT4 +FT3  2 wks post 3^rd^ dose,  Glu, A1c NR. β-OHB↑, ↓ C-pep., +ve GAD65 Ab.  ↓ACTH, cortisol, T  ACTH stimulation test abn | MRI pituitary: NL | NR | HD steroids + β blocker. When ↑TSH, Lt4 started  Insulin for DKA.  HD steroids for hypopituitarism | Insulin-dependent  LT4 NR |
| Miyoshi Y  et al (2016) (146) | Melanoma  (n=1) | 66/F  No PHx & FHx of DM.  Hx of AutoD NR | Nivo/2 mg/kg q 3 wks x 6 doses | DKA Sx/18weeks  Drug D/C ?: No | ↑A1c, 3↑ Glu, ↑β-OHB, ↓pH, ↑anion gap, ↓ C-pep, ↑lipase.-ve GAD 65, IA2 & ZNTr Ab. TSH NL | Abd US & MRI pancreas NL | NR | Insulin | insulin-dependent |
| Okamoto M et al (2016) (147) | Melanoma  (n=1) | 55/F  No PHx of DM  FHx DM & Hx AutoD NR | Nivo/2 mg/kg  q 3 wks | ↑Glu Sx/52 weeks  Drug D/C ?: Yes | ↑A1c, 3↑Glu,↓C-pep, -ve GAD65, IA2, Ins, ZnT8 Abs.  DRB1*04:05-DQB1*04:01  ↓TSH, +ve TPO, TG Ab | CT Abd: mild pancreatic atrophy | NR | Insulin  FTID | Insulin-dependent |
| Usui Y  et al (2016) (148) | Non-small cell lung cancer  (n=2) | C1: 31/M  C2: 62/W  PHx of DM in both. FHx of DM & AD NR. | C1: Nivo/2 mg/kg q3wks  C2: Nivo/2mg/kg | C1: DKA Sx/2 wks  C2: ↑Glu Sx/8 w ks .  Drug D/C ?: Yes | C1: NL HbA1c,4↑ Glu, ↓pH, +ve GAD65 Ab,  DRB1*04:05-DQB1*04:01.  C2: NL A1c, 2↑Glu, ↓ C-peptide,-ve GAD65 Ab. HLA DRB1*09:01-DQB1*03:03 | CT Abd: Shrinkage of metastatic lesions. Pancreas NR | NR | Insulin  FTID | Insulin-dependent |
| Kong SH  et al (2016) (149) | Squamous Cell carcinoma of lung  (n=1) | 68/M  No PHx, FHx of DM and AutoD. | Chemotherapy x2, then Pembro 10 mg/kg x7 wks. | Initially hyperglycemia, then in 5 days DKA symptoms /21 wks | ↑A1c, ↑Glu,↓pH, ↑anion gap, ↓C-pep,  -ve GAD65,IA2, Ins Ab.  DRB1*09:01-DQB1*03:03  ↓TSH, NL FT4. -ve TRAb | CT abd: pancreas-titis | NR | Glimepiride, then DKA Rx, Insulin | Insulin |
| Shah M et al (2016) (150) | Squamous cell lung cancer (n=1) | 77/F  No PHx, FHx of DM and AutoD | Nivo Dose NR | Fatigue, abd pain, weakness, polyuria, polydipsia/1.5 wks  Drug D/C? No | ↑A1c, ↑Glu, ↓pH, ↑ β-OHB , ↓ C-peptide,  -ve GAD65, Ins Ab., IA2,I slet peptide,  -ve GAD65 Ab cell Ab | NR | NR | Fluids, insulin | insulin |
| Zaied A, Lee A (2016) *  (151 ) | Renal cell carcinoma (n=1) | 70/M  No PHx, FHx of DM and AutoD. | Nivo 3mg/Kg  q2 wks | Dyspnea, abd pain, polyuria/10 wks  Drug D/C NR | ↑A1c, ↑Glu, ↓pH, ↑ β-OHB , ↓ C-peptide  -ve antiGAD 65 Ab | Chest X-ray: pleural effusions | NR | Fluids, insulin | insulin |
| Humayun MA (2016)† (75) | Melanoma (n=1) | 55/M  No PHx & FHx of DM and AutoD. | 1^st^ RX: dacarbazine. 2^nd^ Rx: Ipil Dose NR 4 doses over 2 wks  3^rd^ line Rx: Pembro Dose NR x 10 cycles | After Ipi: Fatigue & blurry vision. After Pembro: Fatigue, polydipsia, polyuria/27 wks (9 cycles)  Drug D/C? Yes | After Ipi: Had ↓TSH, FT4, Cortisol, LH, FSH, T  After Pembro:↑A1c, ↑Glu, ↓pH, ↑ β-OHB , ↓ C-peptide. -ve antiGAD 65 Ab | MRI of pelvis: Edema & degenerative changes | NR | After Ipi: HD steroids for hypopituitarism.  After Pembro: Fluids, insulin | Insulin, HCT |
| Tsiogka A et al (2017) (152)† | Melanoma (n=1) | 64/M  No FHx of DM,  Hx of AutoD NR. | Initially surgery & chemotherapy. Ipil 3mg/kg q3wks | Polydipsia, confusion,, vomiting, fever./11 wks  Drug D/C ?: NR | ↑A1c, ↑Glu,↓pH, ↑anion gap, ↓C-pep,  -ve GAD65,IA2, Ins Ab,.  DRB1*0708-DQB1*0204 | NR | NR | Fluids, insulin  FT1D | insulin |
| Abdul Aziz MHF et al (2017) (153) | Melanoma  (n=1) | 48/F  No PHx +FHx of DM.  Hx of AutoD NR | Pembo/2mg/kg  q 2 wks | DKA Sx/2 weeks  Drug D/C?: No | ↑A1c, ↑Glu, ↓pH, ↑anion gap, ↓ C-peptide,  +ve GAD65 & Ins Ab.  -ve Thyroid & Adrenal Ab | NR | NR | Insulin | Insulin-dependent |
| Chae YK  et al (2017) (154) | Lung adeno carcinoma  (n=1) | 76/M  No PHx of DM  FHx of DM and Hx of AutoD NR. | Chemo + Pembo q3wks/ Dose NR | ↑ Glu Sx/5 wks  Drug D/C ?: No | NR HbA1c, ↑ Glu,↓ C-pep, +ve GAD65 & IA2 Ab.  NL thyroid tests. | NR | NR | Insulin,  HD steroids x25 days to reverse T1DM failed | Insulin dependent |
| Li L et al (2017) (134) | Lung squamous cell carcinoma (n=1) | 63/M  No Hx of DM.  FHx of DM & AutoD NR | Nivo dose NR | DKA Sx / 4 weeks  Drug D/C ?: initially no, then yes when DKA recurred. | ↑A1c, ↑Glu, ↓pH, ↑anion gap,+ve GAD65 Ab.  TSH NL then 1 m later ↑↑.  +ve TPO Ab | NR for pancreas. | NR | Insulin  LT4 | Insulin-dependent  LT4 |
| Teramoto Y et al (2017) †  (155) | Melanoma  (n=1) | 63/F  PHx, FHx of DM & Hx AutoD NR | Nivo/2 mg/kg q3wks x8 doses, then D/C | DKA Sx/6 weeks after Nivo D/C.  Drug D/C ?: Yes | ↑A1c, ↑Glu, ↓pH, ↓HCO_3_, (ketones)_s_,↓ C-peptide, -ve  GAD65, IA2, Ins Ab.↑elastase. | NR | NR | Insulin  FTID | Insulin-dependent |
| Thoreau B et al (2017) (156) | Melanoma  (n=1) | 73/M  No PHx of DM.  FHx of DM & AutoD NR | Pembro 10mg/kg q 3 wks x8 doses | DKA Sx/26 weeks  Drug D/C ?: | ↑A1c, ↓pH, NR Glu and C-pep  -ve GAD 65 & IA-2 Abs. | Abd CT:NL pancreas. | NR | Insulin  FTID | Insulin-dependent |
| Araujo M et al (2017) (157) | Non-small cell lung cancer  (n=1) | 73/F  No PHx, FHx of DM, & Hx of AutoD. | Nivo 3 mg/kg q 2 wks | DKA Sx/ 5.5 wks  Drug D/C ?: NR | ↑A1c,↑Glu, ↓ C-pep, ↓pH, ↓HCO_3_, (ketones)_s_,  +ve GAD 65 Ab.HLA  DRB1*03:01–DQA1*05:01–DQB1*02:01/ DRB1*04:01–DQA1*03:01–DQB1*03:02 | Abd US:NL pancreas | NR | Insulin  FTID | Insulin-dependent +25 more doses of Nivo |
| Atkins PW & Thompson D (2017)† (158) | Tonsil Squamous cell carcinoma  (n=1) | 50/M  No PHx, FHx of DM, & Hx of AutoD. | Avelu 10mg/kg q2wks + Utomi dose NR | DKA Sx/4 weeks  Drug D/C ?:NR | ↑A1c, ↑Glu, ↓pH, ↑β-OHB, ↓ C-peptide, +ve GAD65 Ab,  HLA NR | NR | NR | Insulin | Insulin-dependent |
| Chan JTK et al (2017) (159) | Non-small cell lung cancer (n=1) | 78/F  PHx, FHx of DM & Hx AutoD NR. | Chemo before Nivo 3 mg/kg q 2 wks | DKA Sx/6 weeks  Drug D/C ?: Yes | A1c NR, ↑Glu, ↓pH, ↑(ketones)_s_, ↓ C-peptide,  +ve GAD65 Ab. | NR | N | Insulin | Insulin-dependent |
| Chan PY  et al (2017) (160) | Melanoma  (n=1) | 74/M  PHx,FHx of DM & Hx of Auto D NR. | Ipi 3m/kg +Nivo 1 mg/kg q 3 wks x 4 doses, then Nivo 3mg/kg q 2 wks. | DKA Sx /2 wks after maintenance dose of Nivo started  Drug D/C ?: Yes | A1c, Glu and C-peptide NR.  –ve GAD Ab. | CT Abd: ↓ in lesions. Pancreas NR. | NR | Insulin, HD steroids for anterior uveitis & GRADE 3 transaminiitis. | NR |
| Daltry S  et al (2017)* (161) | Renal Cell Carcinoma  (n=1 ) | 54/M  PHx of DM & AD NR  Has FHx of T2DM | Nivo/280 mg q 2 wks | DKA Sx /16 weeks  Drug D/C ?: NR | ↑A1c, ↑Glu, ↑ketone,  ↓ C-peptide, -ve GAD 65 Ab | NR | NR | Insulin | NR |
| Farrell CM et al (2017)* (162) | Melanoma  (n=1) | 30/M  No FHx of DM.  FHx of DM & Hx of AutoD NR | Pembro dose NR x3 doses | DKA Sx/10 weeks  Drug D/C ?: NR | ↑A1c, ↑Glu, ↓pH, ↓HCO_3_,  ↓ C-peptide. –ve GAD 65 & IA-2 Ab | Abd CT  NL pancreas | NR | Insulin | Insulin-dependent |
| Gauci ML  et al (2017) (163) | Melanoma  (n=1) | 73/M  No PHx, FHx of DM.  Has Hx of AutoD (Graves’ disease) | Was on interferon & chemo before starting Nivo/3 mg/kg q 2 wks | DKA Sx/6 weeks  Drug DC ?: Yes | ↑A1c, ↑ Glu, ↓pH, ↓HCO_3_,↓ C-peptide, +ve GAD65, IA-2 & ZnT8 Ab. Had these Ab prior to Nivo Rx when normoglycemic. | NR | NR | Insulin | NR |
| Hickmott L et al (2017) (164) | Urothelial cancer  (n=1) | 57/M  No PHx of DM.  FHx of DM & AutoD NR. | Atezo 1200 mg q3 wks | ↑ Glu Sx/15 wks  Drug D/C ?: Yes | ↑A1c, ↑Glu, ↓ C-pep, ↑anion gap. ↓pH  -ve IC & GAD65 Ab.  NL TSH, ↑FT4.  HLA DRB1*04 & DQB1*0  Cortisol: NL.. | MRI pituitary: NL | NR | Insulin | Insulin |
| Ishikawa K et al (2017)(165) | Melanoma  (n=1) | 54/M  PHx, FHx of DM & AutoD NR. | Nivo/2 mg/kg q 3 wks | ↑Glu Sx/ 48 wks  Drug DC ?: No | ↑A1c, ↑ Glu, ↓ C-peptide  -ve GAD 65 Ab. HLA: HLA-B*15:01, *40:06, DRB1*04:05, *04:06, DQB1 *03:02, & *04:01 | Abd CT: ↓↓ pancreatic volume | NR | Insulin  FTID | Insulin |
| Leonardi GC et al (2017) (166) | Non-small cell lung cancer  (n=1) | 66/M  No PHx of DM.  FHx of DM & AutoD NR | Pembro 2 mg/kg q 3 wks | DKA Sx/11 weeks  Drug D/C ?: No | ↑A1c, ↑Glu, ↓pH, ↑anion gap, ↑β-OHB, ↓ C-pep.  +ve GAD65 Ab. | ↓lesion in lung  Pancreas NR | NR | Insulin | Insulin-dependent |
| Matsumura K et al(2017) (167) | Lung adeno- squamous carcinoma  (n=1) | 68/M  6 yrs ago had DM due to partial pancreatectomy for Pancreatic Ca & steroid Rx.FHx of DM & AutoD NR | Surgery + Chemo; Then, Nivo 3mg/kg q 2 wks. | ↑Glu Sx/6 wks  Drug D/C ?:Yes | ↑A1c, ↑Glu, ↓ C-pep, -ve GAD 65, IA2 and Ins Ab.  HLA A*24:02 and DRB1*09:01 | Abd CT NL pancreas | NR | Insulin, steroids (part of chemo regimen) | NR |
| Mizab MC et al(2017)† (168) | Melanoma  (n=1) | 58/M  PHx, FHx of DM & Hx of AutoD NR. | Pembro 2 mg/kg q 3 wks | DKA Sx/12 weeks  Drug DC ?: NR | ↑A1c, ↑Glu, ↓pH,  ↓ C-peptide,  -ve GAD65 & IA-2 Ab. | NR | NR | NR; Presumed insulin.  FTID | NR |
| Munakata W et al (2017) (169) | Hodgkin lymphoma  (n=1) | 72/M  No PHx of DM.  FHx of DM & AutoD NR | Nivo/3 mg/kg q 2 wks | ↑Glu/12 wks  Drug D/C ?:Yes | ↑A1c,↑Glu, ↓ C-pep,  -ve GAD65, IA-2 & ZnT8 Ab.  ↑lipase. | Abd MRI: pancreatic inflammation | NR | Insulin  FTID | Insulin-dependent |
| Telo GH  et al (2017)* (170) | Renal Cell Carcinoma  (n=1) | 51/M  No PHx, FHx of DM. Hx of AutoD NR | Nivo + Ipi dose NR | DKA/6 weeks  Drug D/C ?:Yes | ↑A1c, ↑Glu, ↓pH, ↓HCO_3_, ↓ C-pep, Ketones 3+,  -ve GAD65 & IA-2 Ab.  ↑lipase | Abd CT:  NL pancreas | NR | Insulin  FTID | NR |
| Wright LAC et al (2017) (171) | C1: Lung Squamous cell carcinoma  (n=1)  C2: Melanoma (n=1) | C1: 78/M  Has T2D x30 years. No Hx of AutoD & FHx of DM  C2: 55/M  T2D x4 yrs & Hashimoto’s thyroiditis  No FHx of DM | C1: Chemo x 3m,then Nivo. Dose NR  C2: Ipi dose NR D/C because of hypophysitis. Then Pembro dose NR. | C1: ↑Glu Sx/ 5 wks  Drug D/C ?: NR  C2: ↑ Glu Sx/ 8 wks  Drug D/C?: NR | C1: A1c NR, ↑Glu, +ve GAD65 Ab.  C2: ↑A1c, ↑Glu, ↓C-peptide, +ve GAD65 Ab. | C1: NR  C2:NR | NR | C1: glyburide→glyburide + metformin →Insulin  C2: glipizide →glipizide+ metformin→sitagliptin →insulin | Insulin |
| Alzenaidi AA et al (2017)* (172) | Melanoma  (n=1) | 47/M  Had type 2 DM 2 yrs ago. FHx of DM &Hx of AutoD NR | Nivo+Ipi Rx Dose NR. | DKA Sx/7 wks  Drug D/C?: NR | ↑A1c, Glu NR, ↓pH, ↑β-OHB, ↓ C-peptide.  +ve GAD65 Ab.  ↑lipase. | CT head & abdomen: no metastasis | NR | DKA Rx; insulin | Insulin |
| Godwin JL  et al (2017)  (173) | Non-small cell lung cancer (n=1) | 34/F  No PHx & FHX of DM. Hx of AutoD NR | Chemo & radiation therapy. Nivo 3mg/kg q3 wks. | DKA Sx/ 8 wks  Drug D/C?: NR | ↑A1c, ↑Glu, ↓pH,↑anion gap, ↓C-pep. +ve GAD65, IA-2, IAA, ZnT18 Ab (All +ve before Nivo).  HLA homozygous for A30 &D9.  NL ACTH & cortisol,  ↓TSH NL FT4 & FT3. –ve TSI &TPO & TG Ab. | Chect Xray: lesion &metastasis. Brain MRI no metastasis. | NR | DKA Rx, insulin | Insulin |
| Changizzadeh PN et al (2017) (174) | Melanoma  (n=1) | 42/M  No Hx of DM.  FHx of DM and AutoD NR. | Surgery. Then, Nivo (1mg/kg) +Ipi (3mg/kg) q3wks x4doses. | DKA Sx, diarrhea/ 9 wks  Drug D/C?: NR | ↑A1c, ↑Glu, ↓pH,↑anion gap. C-pep NR.  NL GAD65, IA-2 & ZnT18 Ab. | CT abd + pelvis:Pan-colitis | NR | HD steroids & octreotide.  DKA Rx +insulin | Insulin |
| Smith-Cohn MA et al (2017) (175) | Cholangiocarcinoma (n=1) | 61/F  No PHx of DM .  +ve FHx of DM.  Hx of AutoD NR. | Surgery, chemo, brachytherapy. Then, chemo + Pembro. Dose NR. | DKA Sx/ 27 wks.  Drug D/C?: No | ↑A1c, ↑Glu, ↓pH. +veGAD65 Ab. ↓Na+.  C-pep NR. | CT abd: pancreas atrophy. | NR | HD steroids,  DKA Rx +insulin | Insulin |
| Alhusseini M et al (2017)† (128) | Adenocarcinoma of lung (n=1) | 65/M  Has Hx of T2DM. FHx of DM & Hx of AutoD NR. | Ipi+Pembro dose NR | DKA Sx/ 3 wks  Drug D/C?: Yes for Ipili & No for Pembro | ↑A1c, ↑Glu, ↓pH, ↓ C-peptide.+veGAD65, islet, insulin Ab.  ↓TSH, ↑FT4. -ve TSI, +ve TPO Ab | Thyroid scan: ↓ uptake. | N | DKA Rx, insulin | Insulin + LT4 |
| Kapke J  et al (2017)  (176) | Maxillary sinus squamous cell carcinoma(n=1)  Urothelial carcinoma (n=1) | C1: 83/M  Hypothyroid.  PHx, FHx of DM & AutoD NR  C2: 63/F  Hypothyroid.  PHx, FHs of DM & AutoD NR | C1:Radiation then Nivo240 mg q2wks.  C2: Surgery, chemotherapy,  Atezo 1200 mg q3wks | C1: DKA symptoms/12 wks  Drug D/C?: NR  C2: DKA symptoms/6 wks  Drug D/C?: NR | C1: A1c NR, ↑Glu, ↓HCO_3_. C-pep,+ve GAD65 Ab.HLA DRB1*08;DRB1*011; DRB!*03;DQB1*04;DQA1*04;DQA1*05  C2: A1c NR, ↑Glu, ↓ HCO_3_.C-pep,+ve GAD65 Ab. HLA DRB1*03;DRB1*04; DQB!*02;DQB1*03;DQA1*03;DQA1*05 | C1: PET scan maxillary sinus lesion  MRI brain: metastasis  C2: NR | NR | C1: Insulin; HD steroid for colitis  C2: Insulin | Both Insulin dependent. |
| Kumagai R et al(2017) †  (177) | Lung adenocarcinoma (n=1) | 73/M  No PHx of DM. FHx of DM & AutoD NR. | Nivo 3mg/kg q2wks. | Fatigue, anorexia, weight loss/22 wks  Drug D/C at 24 wks  Later pneumonitis and vitiligo | Baseline: NL A1c, Glu.  22 wks: ↑A1c,↑Glu,↓C-pep. NL GAD65, IA-2 & ZnT18 Ab. NL anion gap. No DKA  HLA: DRB1*09:01-  DQB1*03:03 and DRB1*01:01-DQB1*05:01. | NR | NR | Insulin & fluids | Insulin |
| Marchand L et al (2017)† (95) | Non-small cell lung cancer:leiomorphic lung carcinoma (n=1) | 55/M  PHx & FHx of DM and AutoD NR. | Chemo; then nivo dose NR x9 doses | DKA Sx / 19 wks.  Asthenia, nausea, ↓BP/32 wks  Drug D/C?: Yes | 19 wks: ↑A1c,↑Glu,↓C-pep.  NL GAD65, IA-2 & ZnT18 Ab.  32 wks: ↓ACTH, cortisol, [Na+]. ↑PRL. Other pituitary hormones NL. | MRI NL pituitary @ 36 wks. | NR | 28 wks: Insulin & fluids  32 wks: HCT | Insulin & HCT |
| Gambale E et al (2017)*  (178) | Melanoma (n=1) | 45/M  PHx &. FHx of DM and AutoD NR. | Pancretodude-nectomy for metastasis; then nivo 3mg/kg q2wks | Symptoms NR/6 wks  Drug D/C: No | Baseline: NL A1c, Glu, islet autoAb.  6 wks: ↑A1c, Glu.  NL C-pep & islet autoAb. | Abd CT scan: pancreas metastasis | NR | NR | NR |
| Lee JYM et al (2017)* (179) | Melanoma (n=1) | 84/M  PHx prediabetes.FHx of DM & AutoD NR. | Pembro 2mg/kg | DKA Sx/ 5 wks.  Drug D/C?: NR | Baseline: Prediabetes A1c  5 wks: ↑Glu, ↓pH, ↑β-OHB,↓C-pep. +ve GAD 65 Ab. | NR | NR | Insulin (800 u/hr) & fluids. HD steroids for insulin resistance | Insulin |
| Nieves CA et al (2017)* (180) | Non-small cell Lung cancer (n=1) | 75/F  PHx predDM & FHx DM. Hx of AutoD NR. | Chemo. Then, Nivo dose NR. | Nausea,polyuria, polydipsia/ 6 wks.  Drug D/C?: Held for 2 wks then restarted. | Baseline: prediabetes.  6 wks: ↑Glu,↑ A1c. No anion gap. NL C-pep then ↓. +ve GAD 65 and IAA Ab. | NR | NR | Insulin | Insulin |
| Reddy SC et al (2017)*  (129) | Non-small cell Lung cancer (n=1) | 66/M  PHx of DM. FHx of DM and AutoD NR | Nivo dose NR. | DKA Sx/4wks  Drug D/C –Yes and gemcitadine started | 4 wks: ↑Glu, ↓pH, ↓C-pep. +ve GAD 65 Ab.  ↓TSH, ↑FT4, FT3. +ve TPO Ab; TSI –ve. | ThyUS: heterogeneous gland | NR | Insulin & fluids.  Methimazole & β-blockers, then high dose steroids | Insulin |
| Reslan Z et al (2017)*  (181) | Melanoma (n=1) | 79/M  PHx & FHx of DM and AutoD NR. | Pembro dose NR x5 doses | Symptoms NR/15 wks.  Drug D/C?: NR | ↑Glu & ↑liver enzymes | NR | NR | Insulin & HD steroids | Insulin |
| Vodopivec DM (2017)*  (182) | Renal cell carcinoma (n=1) | 78/M  No PHx of DM.  FHx of DM and AutoD NR | Nivo 3mg/kg q2wks | Polyuria, polydipsia, fatigue/16 wks.  Drug D/C?: NR | Baseline: NL A1c & Glu  16 wks: ↑Glu, ↑A1c. NL C-pep & anion gap.+ve GAD 65 Ab. | NR | NR | Insulin + fluids | Insulin |
| Hao JB et al (2017) (183) | Melanoma (n=1) | 28/F  No FHx of DM and AutoD. | Initially Ipi, vemurafenib, trametinib. Then  Nivo dose NR x3 doses q2wks | Fatigue, blurry vision, nausea, vomiting/ 9 wks.  Drug D/C? NR | DKA. ↑Glu ..GAD 65 >250 U/mL.Anti insulin, Ia2 and TPO all –ve.  HLA DR3, DQ2. | NR | NR | Insulin + fluids | Insulin |
| Sakurai K  et al (2018)  (130) | Renal cell carcinoma (n=1) | 68/M  No PHx ,FHx of DM. Had subclin hypothyroidism due to Hashimoto’s thyroiditis | Surgery,chemo- & radiation therapy. Then, Nivo dose NR. | Palpitations & fatigue/2 wks  Hyperglycemia Symptoms / 14 wks  Drug D/C?: No | 3wks: ↓TSH, ↑FT$ & FT3.  +ve TPO & TG Ab, -ve TRAb.  9wks: Hypothyroid  14 wks: ↑A1c, ↑Glu, NL pH, ↑β-OHB, ↓ C-peptide. - ve GAD65, IA-2, IAA, ZnT18 Ab. ↑lipase  HLADRB1*09:01-DQB1*03.03  NL adrenal tests. | Thyroid scan: No uptake.  Abdomina CT:Pancreas NL | NR | LT4 & Insulin  FT!D | Insulin + LT4 |
| Villarreal J et al (2018) (184) | Melanoma (n=1) | 66/M  No PHx ,FHx of DM  Had hypothyroidism | Nivo + Ipi Dose NR | Polyuria, polydipsia, light-headeness, fatigue /3 wks.  On HD steroids for colitis a wk before presentation  Drug D/C? Yes | ↑Glu, ↓C-pep  . +ve GAD 65 Ab, IA-2 Ab | NR | NR | Fluids, insulin | Insulin |
| Capitao R et al (2018) (185) | Lung adenoca (n=1) | 74/F  No PHx & FHx of DM and AutoD | Chemotherapy first. Nivo 3mg/kg x2 doses | Polyuria, polydipsia, weight loss, vomiting/3 wks  Drug D/C? No | DKA. ↑A1c, ↑Glu, ↓pH, ↑ anion gap, ↓ C-peptide.+veGAD 65 Ab.  NL ACTH, cortisol, TSH, FT4, LH, FSH for age & sex. | Abd CT no metastatic disease of pancreas | NR | Fluids, insulin | Insulin |

C: Case; *: Abstract; †: Letter to the editor; M: Male; F: Female; Chemo: Chemotherapy; PHx: Personal history; FHx: Family history; AutoD: Autoimmune disease; DM: Diabetes mellitus; T2DM: Type 2 diabetes mellitus; T1DM Type 1 DM; FTID: Fulminant TID; Ipi: Ipilimumab; Nivo: Nivolumab; Pembro: Pembrolizumab; Avelu: Avelumumab; Atezo: Atezolizumab; utomi:Utomilumab; PD-L1: Programmed death ligand-1; Sx: Symptoms; D/C: Discontinued; q:every; wks: weeks; NR: Not reported; NL: Normal; ↓: Decreased; ↑: Increased; abn: abnormal; Glucose: Glucose; DKA: Diabetic ketoacidosis; C-pep: C-peptide; A1c: glycated hemoglobin; GAD 65: Glutamic acid decarboxylase 65; IA-2: Islet Antigen 2; IAA: Insulin auto-antibody; ZnT8: Zinc Transporter 8; βOB: β-OH butyrate; HLA: Human leucocyte antigen; ACTH: Adrenocorticotrophic hormone; LH: Luteinizing hormone; FSH: Follicle stimulating hormone; PRL: Prolactin; Na: Sodium; TSH: Thyroid stimulating hormone; FT4: Free thyroxine; FT3: Free triiodothyronine; TPO: Thyroperoxidase antibodies (Ab); TRAb: Thyroid receptor antibody; Thyroid stimulating immunoglobulin; HCO_3_: Bicarbonate; CTCAW: Common terminology criteria for adverse events; US: Ultrasound; RAI: radioactive iodine; MRI: magnetic resonance imaging; CT: Computerized tomography; PET: Positron emission tomography; ND: Not done; HD=High dose; LT4: levothyroxine; HCT: Hydrocortisone.
