# Supplementary material for: Spectrum of immune checkpoint inhibitors-induced endocrinopathies in cancer patients: a scoping review of case reports
Source: Clin Diabetes Endocrinol. 2019 Jan 22;5:1. doi: 10.1186/s40842-018-0073-4 (PMC6343255; doi:10.1186/s40842-018-0073-4)
Supplement: Supplementary file 6 — Appendix 6. Cases of Immune Checkpoint Inhibitor-Induced Endocrinopathies – Primary Adrenal Insufficiency, Cushing’s Syndrome, Hypoparathyroidism, Diabetes Insipidus. (DOCX 22 kb) [file 40842_2018_73_MOESM6_ESM.docx]

**Appendix 6: Cases of Immune Checkpoint Inhibitor-Induced Endocrinopathies –**

**Primary Adrenal Insufficiency, Cushing’s Syndrome, Hypoparathyroidism, Diabetes Insipidus**

| **Authors**  **(Year)**  **(Reference)** | **Cancer/# of patients** | **Age/Gender**  **Pertinent Med History** | **Drug / dose** | **Clinical Sx/onset after 1^st^ dose**  **Drug D/C?:** | **Laboratory investigations** | **Imaging** | **CTCAE**  **Grade** | **Treatment** | **Outcome** |
| --- | --- | --- | --- | --- | --- | --- | --- | --- | --- |
| **Primary Adrenal Insufficiency** |  |  |  |  |  |  |  |  |  |
| Trainer H et al  (2016) (186) | Melanoma  (n=1) | 43/M  PHx, FHx of EndoD & AutoD NR | Nivo 3mg/kg q2w x 4 doses | Fatigue, wt loss, anorexia, etc/  8 wks.  Drug D/C?: NR | ↑ACTH ↓Cortisol,  ↓Abn ACTH stimulation test  ↓Na+ | MRI pituitary  NL | NR | Oral HCT &  Fludrocortisone | Discharged on HCT & Fludrocortisone |
| Yang JC et al  (2007) (16) | Renal Cell Carcinoma  (n=1) | Age/gender NR  PHx, FHx of EndoD & AutoD NR | Ipi 3mg/kg q3wks | Clinical Sx NR/ 36 wks  Drug D/C?: NR | ↑ACTH, ↓cortisol  NL TSH, T4.  ↓T | Enlarged | NR | HCT | HCT |
| Coskun N et al  (2016)* (187) | Lung Adeno- Carcinoma (n=1) | 50/M  PHx, FHx of EndoD & AutoD NR | Nivo dose NR | Nausea, emesis, hypotension, etc/ 1.5 wks  Drug D/C?: NR | ↑↑ACTH  ↓Cortisol  ↓Na+ | MRI brain  NL | NR | iv steroids | NR |
| Akarca FK et al  (2017) (188) | Non-small cell lung carcinoma (n=1) | 52/M  PHx, FHx of EndoD & AutoD NR | Nivo dose NR | Nausea,emesis, dehydrated, etc/  2 weeks  Drug D/C?: NR | ↑↑ACTH  ↓Cortisol  ↓Na+ | Abdominal CT –  few lymph nodes | NR | HD iv prednisolone | Discharged on oral steroids |
| Paepegaey AC et al  (2007) (126) | Melanoma  (n=1) | 55/F  PHx, FHx of EndoD & AutoD NR | Surgery, chemo x2, Pembro 2mg/kg q3 wks x10 doses | Palpitations, ↓weight/16 wks  Drug D/C?: Yes at 30 wks.  Hypotension, Hyppoglycemia/34 wks | 16 wks: ↓TSH, ↑FT4 & FT3. -ve TPO, TG & TR Ab.  18 wks: Hypothyroid  34 wks: ↓cortisol, ↑ACTH, Synactin test abn, +ve adrenal Ab. Nl, FSH, LH, PRL, TSH (on LT4), IGF-1 | Thyroid scan no uptake.  42 wks: CT abd: atrophied adrenals. | NR | 18wks: LT4  34 wks: iv HD steroids, then HCT & Florinef | HCT, Fludrocortisone & LT4 |
| Min L et al  (2013)* (34) | Melanoma  (n=1) | 56/F  PHx, FHx of EndoD & AutoD NR | Ipi dose NR | Fatigue & headache/12 wks  Drug D/C: NR | ↓ACTH & cortisol at onset (12 wks)  Four wks later,  Cosyntropin stimulation test – no cortisol & aldosterone response | MRI pituitary: enlarged at 12 wks.  Abd CT: at 16 wks? enlarged adrenal glands | NR | HCT | Discharged |
| **Cushing’s syndrome** |  |  |  |  |  |  |  |  |  |
| Lupu J et al  (2017) (127) | Melanoma  (n=1) | 53/F  PHx, FHx of EndoD & AutoD NR | Ipi 1mg/kg +Nivo 3mg/ kg q3wks | Thyrotoxicosis/6wks  Then hypothyroid.  Weight ↑, polyuria, depression/9wks.  Drug D/C?: Yes at 12 wks.  Anorexia, weakness/16 wks  Wk 22: Drugs re-started. | 6 wks: ↓TSH, ↑FT4 & FT3. -ve thyroid Ab.  Then ↓TSH, ↓FT4 & FT3.  9wks: Sl ↑cortisol.  12 wks: ↑ACTH, ↑cortisol. Abnormal low dose Suppression test. ↑24 hr [cortisol] _u_  ↓LH, estradiol. NL PRL  16 wks:↓ACTH,cortisol | Thyroid US: Hypoechogenic gland.  MRI: NL pituitary before Rx. At 12 wks: Pituitary enlarged. | NR | Wk6: LT4  Wk 16: HCT & LT4 | HCT & LT4 |
| **Hypoparathyroidism** |  |  |  |  |  |  |  |  |  |
| Win MA et al  (2017) (122) | Melanoma  (N=1) | 73/M  No Hx of AutoD  PHx, FHx of EndoD NR | Nivo + Ipi dose NR | Paresthesia, weakness, etc/ 6 wks | ↓↓ Ca++, undetectable PTH, ↓vitamin D, ↓Mg, ↑PO4.  EKG slightly prolonged QT interval | MRI brain: NL | NR | Iv Ca gluconate,  Vit D & Ca carbonate PO | Put on LT4 in hypothyroid phase 7 weeks after onset. |
| **Diabetes Insipidus** |  |  |  |  |  |  |  |  |  |
| Dillard T et al (2010)  (19) | Prostate Ca  (n=2) | C1: 67/M  C2: 50/M  Hx, FHx of EndoD & AutoD NR | Chemo, interferon,  then Ipi 10mg/kg q4w x4 | C1: Headache, anorexia, asthenia/C1: 4 wks,  C2:Polydipsia, polyuria/12 wks- Diabetes insipidus.  Drug D/C?: No in both | ↓ACTH + cortisol, TSH + FT4,  LH + FSH. Low PRL + IGF1  C 2 ↓ACTH + cortisol, TSH + FT4,LH + FSH. Also had abnormal ACTH stimulation test | C 1:  Pituitary Enhanced Case C2:NL | NR | HD steroids in both cases. | NR |
| Nallapaneni N et al (2014) (41) | Melanoma  (n=1) | 62/M  PHx, FHx of EndoD & AutoD NR | Ipi 3m/kg q3w | Nausea, rash, vomiting, polyuria, polydipsia. /3 wks.  Drug D/C?: NR | ↓TSH, FT4, ACTH, Cortisol, LH, FSH, PRL, IGF-1  Water deprivation = partial diabetes insipidus. | NL | NR | HCT, LT4  Desmopressin | Discharged on these 3 meds |
| Zhao C et al (2018) (189) | Merkel cell carcinoma (n=1) | 73/M  PHx, FHx of EndoD & AutoD NR | Avelumab 10mg/kg q2wks x8doses | Polydipsia, polyuria, nocturia / 16 wks  Drug D/C?: NR | ↑[Na+],[osmolality_]s_  ↓[osmolality]_u_  Nl Glu, TSH, FT4, ACTH, cortisol cosyntropin test, LH, FSH, PRL,IGF-1. | MRI pituitary: NL. | NR | Desmopressin | Six wks later Despopressin D/C |

C: Case; M; Male; F: Female; Chemo: Chemotherapy; NR: Not reported;

PHx: Personal history; FHx: Family history; EndoD: Endocrine disease; AutoD: Autoimmune disease;

Ipi: Ipilimumab, Nivo: Nivolumab; Pembro:: Pembrolizumab;

Sx: Symptoms; Wks: weeks;

ACTH: Adrenocorticotrophic hormone; PTH: Parahormone; TSH: Thyroid stimulating hormone; FT4: Free thyroxine; FT3: Free triiodothyronine; LH: Luteinizing hormone; FSH: Follicle stimulating hormone; T= Testosterone; PRL: Prolactin; GH: Growth hormone; IGF1: Insulin growth factor-1; Na: Sodium; Ca: calcium; Mg: Magnesium; PO_4_: Phosphate;

MRI: Magnetic resonance imaging; US: Ultrasound; CTCAE: Common terminology criteria for adverse events.

Rx: Treatment; HCT: Hydrocortisone; LT4: Levothyroxine; HD steroids: High dose steroids.
